# Calix[2]naphth[2]arene: A Class of Naphthalene–Phenol Hybrid Macrocyclic Hosts

**DOI:** 10.1021/acs.orglett.0c02247

**Published:** 2020-07-20

**Authors:** Rocco Del Regno, Paolo Della Sala, Aldo Spinella, Carmen Talotta, Dalila Iannone, Silvano Geremia, Neal Hickey, Placido Neri, Carmine Gaeta

**Affiliations:** † Laboratory of Supramolecular Chemistry, Department of Chemistry and Biology “A. Zambelli”, Università of Salerno, Via Giovanni Paolo II 132, Fisciano I-84084, Italy; ‡ Centro di Eccellenza in Biocristallografia, Dipartimento di Scienze Chimiche e Farmaceutiche, Università di Trieste, via L. Giorgieri 1, I-34127 Trieste, Italy

## Abstract

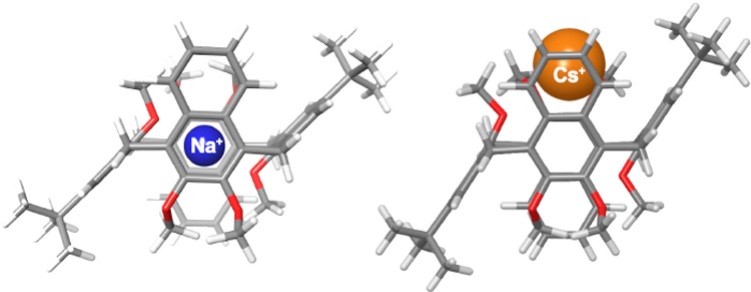

Calix[2]naphth[2]arenes make up a new class of phenol–naphthalene hybrid macrocycles. X-ray studies show that calix[2]naphth[2]arene **1** adopts a 1,2-alternate conformation. Alkali metal cations are complexed by the calixnaphtharenes in a 1,2-alternate conformation, by cation···π interactions with the naphthalene walls, and by RO···M^+^ ion–dipole interactions. In the presence of Cs^+^, chiral complexes of calixnaphtharenes **5** and **6** were observed in which the cation is nested on one of the two faces of the macrocycle.

Macrocycles play a pivotal role in molecular recognition phenomena in which they are considered as the ideal prototype of artificial receptors that can mimic the performance of natural systems.^1,2^ Among the macrocycles studied in supramolecular chemistry, calixarenes,^3^ resorcinarenes,^4^ and pillararenes^5^ are obtained via one-pot condensation between monomeric aromatic units (*p-tert*-butylphenol, resorcinol, and 1,4-dimethoxybenzene, respectively) and paraformaldehyde or aliphatic aldehydes in the presence of an acid catalyst. The calix[4]arene macrocycle can adopt, both in the solid state and in solution, four conformations, named cone, partial cone, 1,3-alternate, and 1,2-alternate.^3^ Among these, the 1,2-alternate conformation is considered a rare conformation in calixarene chemistry.^6^ Reinhoudt first observed the existence of the 1,2-alternate conformation in solution and in the solid state for the *anti*-1,3-diethyl-2,4-dimethyl-calix[4]arene.^6,7^

In macrocyclic chemistry, a growing interest has been devoted to the synthesis of macrocycles starting by naphthalene or anthracene monomers.^8−10^ Very recently, our group reported prism[*n*]arenes,^11^ based on methylene-bridged 1,5-naphthalene units.^11,12^ Naphthol-based macrocycles^13^ such as prismarenes,^11^ oxatubarenes,^9^ naphthotube,^14^ naphthocage,^15−17^ and zorbarenes^8^ can form complexes with ammonium guests by cation···π interactions.

In recent years, different reports have shown that the fragment coupling strategy is a useful synthetic route for building macrocycles constituted by different aromatic units. Following this strategy, Chen reported triptycene-based macrocycles that showed interesting conformational properties and peculiar recognition abilities.^18a^

These considerations prompted us to investigate the fragment coupling synthesis (FCS) of a hybrid naphthalene–phenol macrocycle. Our aim is to combine the conformational features of the calix[4]arene skeleton with the recognition abilities of naphthalene-based macrocycles.

The synthesis of hybrid macrocycle **1** is outlined in Scheme 1. The key step is the fragment coupling reaction between **2**^8^ and **4**. First, derivative **4** was obtained by reaction between derivative **2**^8^ and an excess of *p*-*tert*-butylphenol **3** in the presence of *p*-toluenesulfonic acid as a catalyst, in toluene at reflux (Supporting Information). Finally, derivative **1** was obtained by reaction between **4** and **2** in an equimolar ratio, in the presence of *p*-toluenesulfonic acid as the catalyst and *o*-dichlorobenzene as the solvent, for 6 h (Scheme 1). Macrocycle **1** was isolated in 26% yield after column chromatography. High-resolution FT ICR MALDI mass spectra (Supporting Information) indicate the presence of a molecular ion peak at *m*/*z* 724.3761 in accord with the molecular formula of **1** (calcd *m*/*z* 724.3764 for C_48_H_52_O_6_). We named derivative **1** as calix[*n*]naphth[*m*]arene, in which *n* and *m* indicate the number of phenol and naphthalene units, respectively.

**Scheme 1:**
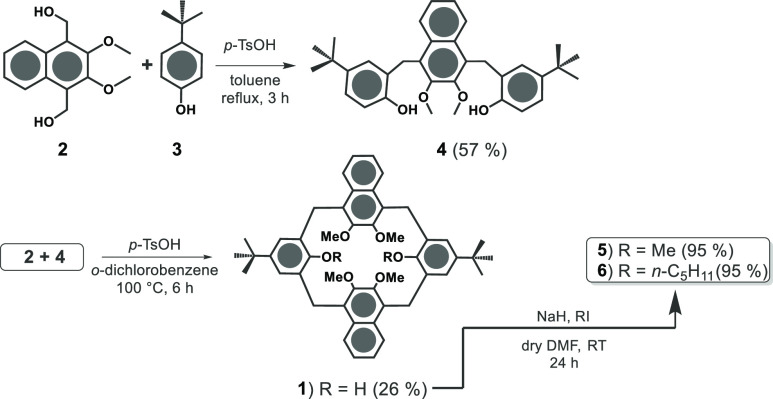
Fragment Coupling Synthesis (FCS) of **1**

To the best of our knowledge, this is the first example of a macrocycle bearing methylene-bridged 1,4-naphthalene units. The calix[2]naphth[2]arene **1** can adopt five possible conformations: cone, partial-cone-1 (paco1), partial-cone-2 (paco2), 1,3-alternate (1,3-alt), and 1,2-alternate (1,2-alt) (Figure 1).^19^ X-ray analysis of a single crystal of **1** obtained by slow evaporation from a CHCl_3_/*n*-hexane solution was performed using synchrotron radiation. In the solid state, derivative **1** adopts a 1,2-alternate conformation (Figure 2).

**Figure 1:**
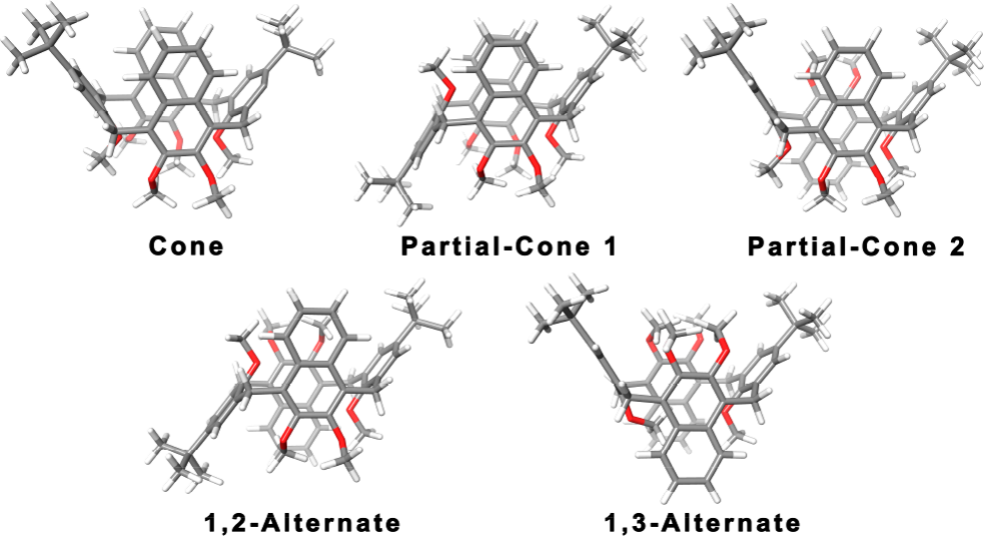
Five possible conformations for the calix[2]naphth[2]arenes.

**Figure 2:**
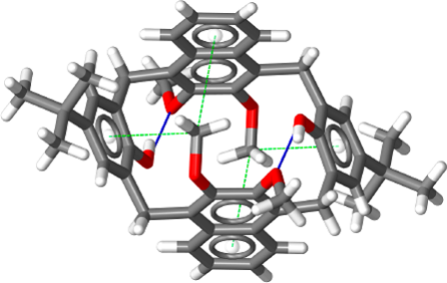
X-ray structure of calix[2]naphth[2]arene **1**.

The molecule crystallized in centrosymmetric triclinic space group *P*1̅. The cyclic molecules lie on crystallographic centers of inversion (*C_i_* molecular point symmetry), and the asymmetric unit contains a half-molecule of **1** and one CHCl_3_ solvent molecule located outside of the macrocycle (see the Supporting Information).

Structurally relevant intramolecular hydrogen bonding interactions are observed between the OH functions as donor groups and the adjacent methoxy oxygen atoms as acceptors, with O···O distances of 2.78 Å (Figure 2, blue). The ^1^H NMR spectrum of **1** at 213 K (Figure 3b) shows a broad ArCH_2_Ar signal indicative of its conformational mobility due to the *O-through-the-annulus* passage.

**Figure 3:**
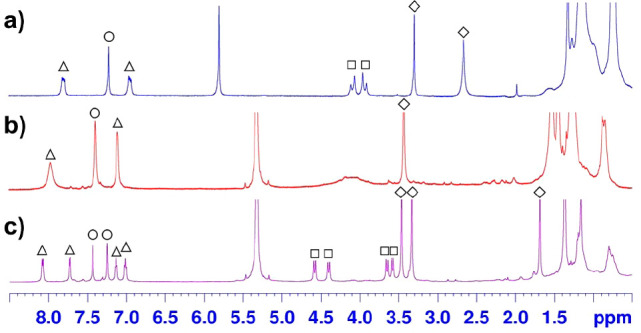
^1^H NMR spectra (600 MHz, CD_2_Cl_2_) of **1** at (a) 298 K, (b) 213 K, and (c) 193 K, marked with (△ and ○) the signals of the aromatic H atoms of naphthalene and phenol rings and (□ and ◇) the signals of the ArCH_2_Ar and OMe groups.

Interestingly, the ^1^H NMR spectrum at 193 K (Figure 3c) clearly shows that calix[2]naphth[2]arene **1** is frozen in the 1,2-alternate conformation. From these studies, an energy barrier of 9.7 kcal/mol was calculated for the *O-through-the-annulus* passage in **1**. The presence of a OMe singlet, shielded at 1.53 ppm (193 K), is in agreement with the solid-state 1,2-alternate structure of **1** (Figure 2), where one of the two naphthalene methoxy groups points inside the cavity of **1** with a OCH_3_···π^centroid^ distance of 3.55 Å (green dashed lines in Figure 2).

Calix[2]naphth[2]arene **1** was alkylated in the presence of MeI and NaH as the base, in dry DMF, for 24 h (Scheme 1). The hexamethoxy-calixnaphtharene **5** was isolated in 95% yield after column chromatography. The ^1^H NMR spectrum of **5** in CD_2_Cl_2_ at 298 K (Supporting Information) shows a broad ArCH_2_Ar signal at 4.00 ppm, indicative of the conformational mobility of the macrocycle, due to the *OMe*-*through-the-annulus* passage. Also in this case, variable-temperature ^1^H NMR experiments (Supporting Information and Figure 4) indicate that below 273 K the hexamethoxy-calixnaphtharene **5** is frozen in the 1,2-alternate conformation (Supporting Information).

**Figure 4:**
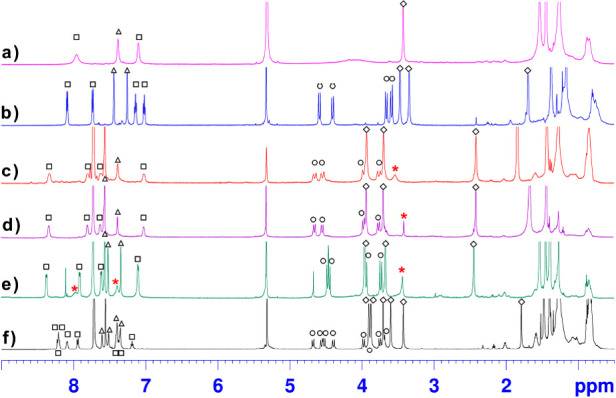
^1^H NMR spectra of **5** in CD_2_Cl_2_ at 600 MHz and (a) 298 K and (b) 193 K and at 298 K for 1:1 mixtures (5.3 mM) of **5** and (c) Li[B(Ar^F^)_4_]^−^, (d) Na[B(Ar^F^)_4_]^−^, (e) K[B(Ar^F^)_4_]^−^, and (f) Cs[B(Ar^F^)_4_]^−^. The signals of free **5** are marked with asterisks. The signals of the aromatic H atoms of the naphthalene and phenol rings are marked with □ and △, and the signals of the ArCH_2_Ar and OMe groups are marked with ○ and ◇.

From these data, an energy barrier of 12.3 kcal/mol (Supporting Information) was calculated for the *OMe-through-the-annulus* passage in **5**. A two-dimensional NOESY spectrum (Supporting Information) indicates the *anti* orientation of the couples of anisole and naphthalene rings, confirming the 1,2-alternate conformation of **5**. In fact, at 193 K, the NOESY spectrum shows the presence of a dipolar coupling between the anisole OMe singlet at 3.33 ppm and the naphthalene H signal at 8.09 ppm (see Supporting Information). Density functional theory (DFT) calculations at the B3LYP/6-31G(d,p) level of theory (see Supporting Information) indicate 1,2-alt is the most stable conformation of **5**. The cone conformation is predicted to be less stable than the experimentally observed 1,2-alt conformation by 2.1 kcal/mol. Analogously, the 1,2-alt is more stable than paco1, paco2, and 1,3-alt by 2.9, 2.5, and 10.9 kcal/mol, respectively (Supporting Information). On the basis of these relative energies, a Boltzmann population at 193 K of 99.4% (1,2-alt), 0.4% (cone), 0.15% (paco2), and 0.05% (paco1) was calculated, which is in agreement with the ^1^H NMR spectrum of **5** (Figure 4b). Interesting cation complexing abilities of **5** were clearly evidenced. In fact, when Na[B(Ar^F^)_4_] {[B(Ar^F^)_4_]^−^ = tetrakis[3,5-bis(trifluoromethyl)phenyl]borate}^20,21^ was added to the CD_2_Cl_2_ solution of **5**, the initial ^1^H NMR spectrum changed dramatically (Figure 4). In particular, the ^1^H NMR spectrum of a 1:1 mixture of **5** and Na[B(Ar^F^)_4_]^−^ at room temperature (Figure 4d) showed the typical features of the 1,2-alternate conformation of **5**, previously seen in Figure 4b. Upon addition of K[B(Ar^F^)_4_] or Li[B(Ar^F^)_4_], the ^1^H NMR spectrum of **5** in CD_2_Cl_2_ undergoes analogous changes (Figure 4b,c,e).

These results clearly indicate that in the presence of Na^+^, K^+^, or Li^+^ cations a conformational templation occurs, which blocks the 1,2-alt conformation of calix[2]naphth[2]arene **5** already at room temperature (with respect to the NMR time scale). The structure of the Na^+^**⊂5** complex was investigated by DFT calculations (see the Supporting Information and Figure 5a–c).

**Figure 5:**
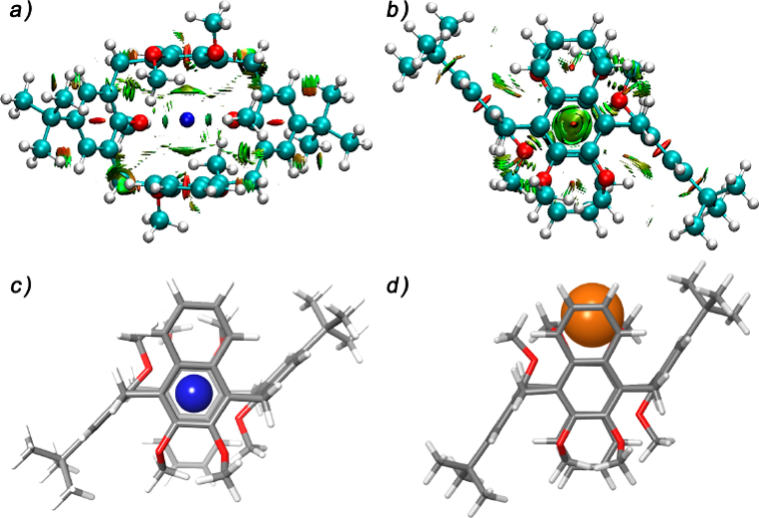
(a and b) Gradient RDG isosurfaces (0.5) for the noncovalent interaction regions in the Na^+^**⊂5** complex. DFT-optimized structures of the (c) Na^+^**⊂5** and (d) Cs^+^**⊂5** complexes at the B3LYP/6-31G(d,p) and B3LYP/SDD levels of theory.

Interestingly, the sodium cation is located inside the macrocycle cavity (Figure 5a–c) and is perfectly sandwiched between the two oxygenated rings of the naphthalene units, to give cation···π interactions (Na^+^···π^centroid^ distance of 2.77 Å), while the two *anti*-oriented anisole rings stabilize the complex by opposite MeO···Na^+^···OMe ion–dipole interactions (Na^+^···OMe distance of 2.35 Å).

Natural bond orbital (NBO)^22^ and noncovalent interaction (NCI)^23^ (see the Supporting Information and Figure 5a,b) analyses were performed on complexes Na^**+**^**⊂5** and K^**+**^**⊂5** using the B3LYP/6-31G(d,p) level of theory to identify the second-order interactions between the host and guest. Both of these studies indicate that the sandwiching cation···π interactions involving the two oxygenated naphthalene rings play a crucial role in the stabilization of the complexes. In fact, the cation···π interactions account for 42% and 33% of the total interaction energy for the Na^**+**^**⊂5** and K^**+**^**⊂5** complexes, respectively. Lone pair···cation interactions between the oxygen atoms of the *anti*-oriented anisole rings are stronger for K^+^ than for Na^+^, while a minor contribution was given by the OMe groups of 2,3-dimethoxynaphthalene units. The MeO^anisole^···cation interactions account for 6.8% and 16.7% of the total energy for the Na^**+**^**⊂5** and K^+^**⊂5** complexes, respectively. An association constant value of (2.2 ± 0.2) × 10^3^ M^–1^ was calculated^24^ for the Na^**+**^**⊂5** complex at 298 K in CD_2_Cl_2_ by integration of the slowly exchanging ^1^H NMR signals of the free host and complex. In a similar way, values of (2.5 ± 0.3) × 10^3^ and (2.0 ± 0.3) × 10^3^ M^–1^ were calculated for the K^+^ and Li^+^ complexes of **5**, respectively. The 1,2-alt structure of the calix[2]naphth[2]arene macrocycle was blocked by alkylation of the OH groups of **1** with 1-iodopentane, under the conditions reported in Scheme 1. The corresponding derivative **6** was obtained in 95% yield. The ^1^H NMR spectrum of **6** in CD_2_Cl_2_ at 298 K (Supporting Information) shows the typical features observed at low temperatures for the 1,2-alt conformation of calix[2]naphth[2]arenes **1** and **5**. Interestingly, with an increase in the temperature of a TCDE solution of **6**, no hint of coalescence or broadening was detected in its ^1^H NMR spectrum. Analogously, the ^1^H NMR spectrum of **6** in TCDE remained unchanged even after heating at 393 K for 12 h. These results clearly indicate that derivative **6** adopts a stable 1,2-alt structure in which the two pentyl groups prevent the *OR-through-the-annulus* passage. Finally, the formation of the Li^+^**⊂6**^1,2-alt^, Na^+^**⊂6**^1,2-alt^, and K^+^⊂**6**^1,2-alt^ complexes was ascertained by ^1^H NMR analysis in CD_2_Cl_2_ at 298 K (Supporting Information), with association constants of (1.5 ± 0.3) × 10^3^, (3.7 ± 0.3) × 10^3^, and (5.1 ± 0.6) × 10^3^ M^–1^, respectively, calculated by qNMR.^24^

Interestingly, when Cs[B(Ar^F^)_4_] was added to the CD_2_Cl_2_ solution of **5**, then the resulting ^1^H NMR spectrum of the mixture in Figure 4f was compatible with formation of a chiral Cs^+^**⊂5**^1,2-alt^ complex [*K*_ass_ = (3.0 ± 0.2) × 10^3^]. In fact, four AX systems (eight doublets, marked with **○** in Figure 4f), 12 aromatic signals, and six OMe singlets were present in the ^1^H NMR spectrum of the Cs^+^**⊂5**^1,2-alt^ complex (Figure 4f). Clearly, the chirality of the Cs^+^**⊂5**^1,2-alt^ complex is compatible only with the formation of a structure devoid of the inversion center maintained in the Li^+^**⊂5**^1,2-alt^, Na^+^**⊂5**^1,2-alt^, and K^+^**⊂5**^1,2-alt^ complexes. DFT calculations at the B3LYP/SDD^25^ level of theory are in agreement with this conclusion. The optimized structure of the Cs^**+**^**⊂5**^1,2-alt^ complex reported in Figure 5d shows that the Cs^+^ cation is nested on one side of macrocycle **5**, therefore establishing cation···π interactions with a pair of *syn*-oriented naphthalene and anisole rings, as well as MeO···Cs^+^ ion–dipole interactions with the methoxy groups of the other two oppositely oriented aromatic rings. An analogous behavior was observed for macrocycle **6** upon addition of Cs[B(Ar^F^)_4_]. The formation of the chiral Cs^**+**^**⊂6**^1,2-alt^ complex was observed, with a *K*_ass_ of (1.7 ± 0.2) × 10^3^.

In conclusion, here we report a novel class of hybrid macrocycles named calix[2]naphth[2]arenes. These hybrid macrocycles combine the conformational features of calix[4]arenes with the recognition abilities of the naphthalene-based macrocycles. In particular, by blocking the 1,2-alternate conformation, alkali metal cations are complexed by the calixnaphtharenes. In the presence of Cs^+^, a chiral complex of calixnaphtharenes **5** and **6** was observed in which the cation remains nesting on one of the two equivalent faces of the macrocycle. The cation···π interactions between cationic guests and naphthalene walls play a crucial role in the stabilization of the complexes.

## Supporting Information Available

Detailed synthetic procedures, one-dimensional–two-dimensional NMR spectra of calix[2]naphth[2]arenes and their complexes, HR mass spectra, details of DFT calculations, X-ray data, and tables of crystal data (PDF)

## Accession Codes

CCDC 1991495 contains the supplementary crystallographic data for this paper. These data can be obtained free of charge via www.ccdc.cam.ac.uk/data_request/cif, or by emailing data_request@ccdc.cam.ac.uk, or by contacting The Cambridge Crystallographic Data Centre, 12 Union Road, Cambridge CB2 1EZ, UK; fax: +44 1223 336033.
