# Supplementary material for: Calix[2]naphth[2]arene: A Class of Naphthalene–Phenol Hybrid Macrocyclic Hosts
Source: Org Lett. 2020 Jul 20;22(15):6166–70. doi: 10.1021/acs.orglett.0c02247 (PMC8009595; doi:10.1021/acs.orglett.0c02247)
Supplement: Supplementary file 1 — ol0c02247_si_001.pdf [file ol0c02247_si_001.pdf]

Supporting Information

Calix[2]naphth[2]arene: a Class of Naphthalene-Phenol Hybrid Macrocyclic Hosts

Rocco Del Regno,[†] *Paolo Della Sala,*[†] *Aldo Spinella,*[†] *Carmen Talotta,*[†] *Dalila Iannone,*[†] *Silvano Geremia,*[‡] *Neal Hickey,*[‡] *Placido Neri*[†] *and Carmine Gaeta*[†,*]

[†]Laboratory of Supramolecular Chemistry, Department of Chemistry and Biology "A. Zambelli", University of Salerno, Via Giovanni Paolo II, Fisciano, I-84084, Italy. [‡] Centro di Eccellenza in Biocristallografia, Dipartimento di Scienze Chimiche e Farmaceutiche, Università di Trieste, via L. Giorgieri 1, I-34127 Trieste, Italy

Table of Contents

	Pages
General experimental details	S2
Synthesis of derivatives 1, 4, 5, and 6	S2 – S4
Copies of 1D, 2D NMR and HR mass spectra	S5 – S30
Complexation studies and copies of NMR spectra of the complexes	S31 – S47
^{1}H NMR determination of K_{ass} values.	S48 – S56
X Ray Details of **1**	S57 – S59
Conformational studies by DFT calculations: cartesian coordinates and single point energies of the 5 conformers of the calix[2]naphtha[2]arene **5**	S60 – S71
DFT-optimized structures and cartesian coordinates of the complexes	S72 – S81
NBO and NCI analysis	S82 – S83
References	S84

General Experimental Details

HR MALDI mass spectra were recorded on a Bruker Solarix FT-ICR mass spectrometer equipped with a 7T magnet. The samples recorded in MALDI were prepared by mixing 10 μL of analyte in dichloromethane (1 mg/mL) with 10 μL of solution of 2,5-dihydroxybenzoic acid (10 mg/mL in Methanol). The mass spectra were calibrated externally, and a linear calibration was applied. All reaction solvents were dried by activated 3 Å molecular sieves.[1] All chemicals reagents grade was used without further purification and were used as purchased. Reaction temperatures were measured externally. Reactions were monitored by TLC silica gel plates (0.25 mm) and visualized by UV light 254 nm, or by spraying with H_2SO_4-$Ce(SO_4)_2$. NMR spectra were recorded on a Bruker Avance-600 [600 (^{1}H) and 150 MHz (^{13}C)], Avance-400 [400 (^{1}H) and 100 MHz (^{13}C)] or Avance-300 MHz [300 (^{1}H) and 75 MHz (^{13}C)] spectrometers. Chemical shifts are reported relative to the residual solvent peak[2]. Standard pulse programs, provided by the manufacturer, were used for 2D COSY-45, 2D HSQC and 2D NOESY experiments.

The derivative **2** was synthesized according to literature procedures.[3] The ^{1}H and ^{13}C NMR spectra of **2** are in accord with those reported in literature.[3]

Synthesis of derivatives 1, 4, 5, and 6

Synthesis of derivative 4

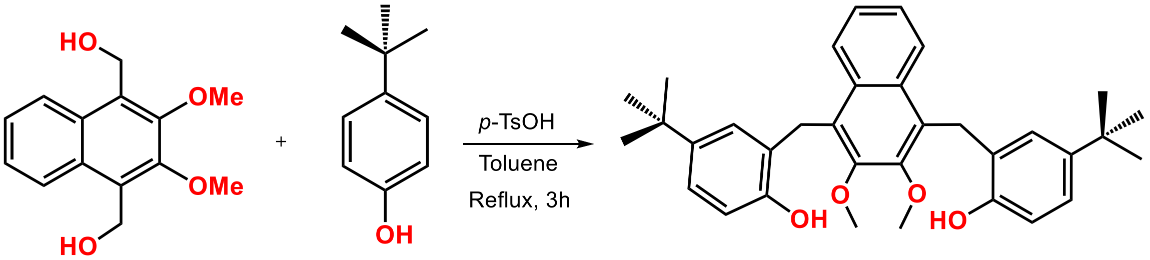

2 **3** **4**

A mixture of **2**[3] (3.00 g, 12.10 mmol), *p-tert*-butylphenol **3** (18.16 g, 120.90 mmol) and *p*-toluenesulfonic acid *p*-TsOH (2.30 g, 12.10 mmol) in toluene (1200 mL) was stirred at reflux, 110 °C, in an oil bath, for 3 h. After cooling at room temperature, a saturated solution of $NaHCO_3$ (500 mL) was added and the aqueous layer was extracted with $CHCl_3$ (3 x 150 mL). Then, the organic layer was washed with water (100 mL), dried over Na_2SO_4, filtered and the solvent was evaporated under vacuum. The crude product was purified through chromatographic column on silica gel (petroleum ether/ EtOAc = 9:1, v/v) to give the pure product **4** (3.50 g, 57 %) as a white solid.

Mp: 180.2-181.2 °C.

^{1}H NMR (600 MHz, $CDCl_3$, 298 K): δ 8.30 (m, ArH, 2H), 7.46 (m, ArH, 2H), 7.43 (d, J = 2.4 Hz, ArH ,2H), 7.34 (s, O*H*, 2H), 7.09 (m, ArH, 2H), 6.74 (d, J = 8.4 Hz, ArH, 2H), 4.33 (s, ArC*H₂*Ar, 4H), 4.08 (s, OC*H₃*, 6H), 1.25 (s, *t*-Bu,18H).

^{13}C-NMR (150 MHz, $CDCl_3$, 298 K): δ 152.6, 147.8, 142.7, 131.1, 128.2, 127.4, 125.6, 125.3, 124.9, 124.2, 116.1, 62.2, 34.1, 31.7, 27.5.

HRMS (FT-ICR MALDI) m/z $[M]^+$ calcd for $C_{34}H_{40}O_4$: 512.2927; found: 512.2926.

Synthesis of derivative 1

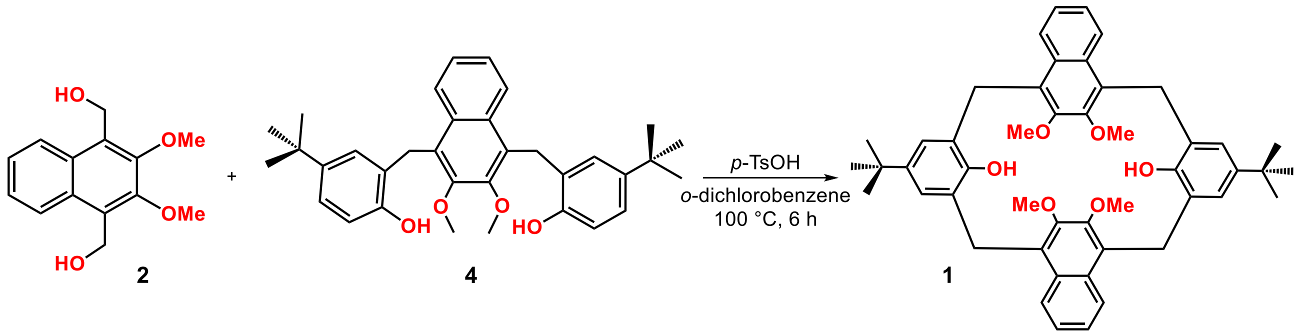

To a solution of *p*-TsOH (0.02 g, 0.11 mmol) in *o*-dichlorobenzene (32 mL) was added a solution of **2** (0.05 g, 0.21 mmol) and **4** (0.11 g, 0.21 mmol) in *o*-dichlorobenzene (64 mL) under nitrogen atmosphere at 100 °C (oil bath). The mixture was stirred at 100 °C for 6 h. The solution was evaporated in vacuum and then the mixture was separated by chromatographic column on silica gel (petroleum ether/ CH_2Cl_2 = 6:4, v/v) to give the pure product **1** (0.040 g, 26 %).

Mp: > 300 °C dec.

^{1}H NMR (400 MHz, $CDCl_3$, 298 K): δ 8.04 (m, ArH, 4H), 7.32 (s, ArH, 4H), 7.22 (m, ArH, 4H), 6.05 (s, O*H*, 2H), 4.25 and 4.06 (AB system, J = 14.8 Hz, ArC*H₂*Ar, 8H), 2.73 (s, OC*H₃*, 12H), 1.44 (s, *t*-Bu, 18H).

^{13}C-NMR (150 MHz, $CDCl_3$, 298 K): δ 151.9, 148.8, 141.3, 130.3, 127.2, 127.1, 126.5, 124.9, 123.9, 60.0, 34.1, 32.0, 29.4.

HRMS (FT-ICR MALDI) m/z $[M]^+$ calcd for $C_{48}H_{52}O_6$: 724.3764; found: 724.3761.

General procedure for the synthesis of derivatives 5 and 6

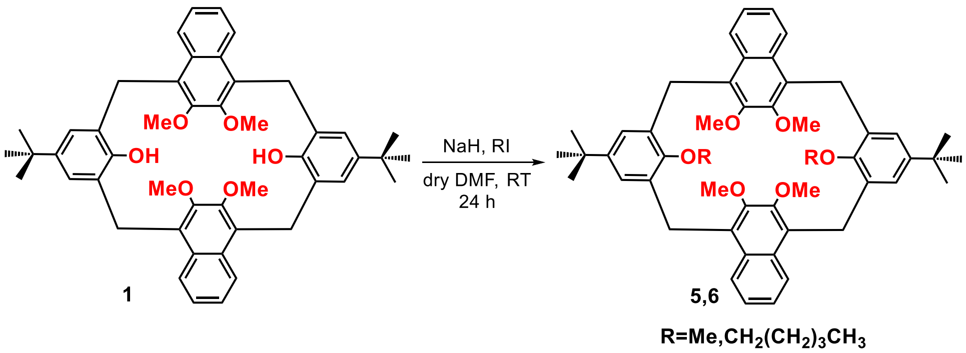

To a solution of **1** (15.00 mg, 0.02 mmol) in dry DMF (7 mL) was added NaH (8.30 mg, 60% dispersion in mineral oil, 0.21 mmol) under nitrogen atmosphere at 0 °C. The mixture was stirred for 1 h at room temperature. Then alkyl iodide (1.03 mmol) was slowly added and the resulting solution was stirred for 24 h at room temperature. After, 10 mL of 1 M solution of HCl was added. The mixture was extracted with $CHCl_3$ (3 x 20 mL), and the organic layer was dried over Na_2SO_4, filtered and concentrated under reduced pressure. The crude product was purified through chromatographic column on silica gel (petroleum ether/ CH_2Cl_2 = 6:4, v/v).

Derivative 5:

The macrocycle **5** was obtained in 95% yield (15 mg) as a white solid.

Mp: > 300°C dec.

^{1}H NMR (300 MHz, TCDE, 373 K): δ 7.80 (m, ArH, 4H), 7.22 (s, ArH, 4H), 6.94 (m, ArH, 4H), 4.06 and 3.96 (AB system *J* = 14.4 Hz, ArCH_2Ar, 8H), 3.30 (s, OCH_3, 6H), 2.67 (s, OCH_3, 12H), 1.33 (s, *t*-Bu,18H).

^{13}C-NMR (75 MHz, TCDE, 373 K): δ 157.0, 150.8, 145.2, 134.7, 130.2, 128.0, 127.1, 124.7, 122.7, 61.3, 59.5, 34.3, 31.9, 29.8.

HRMS (FT-ICR MALDI) m/z $[M]^+$ calcd for $C_{50}H_{56}O_6$: 752.4077; found: 752.4082.

Derivative 6:

The macrocycle **6** was obtained in 95 % yield (17 mg) as a white solid.

Mp: > 300°C dec.

^{1}H NMR (600 MHz, TCDE, 298 K): δ 7.87 (d, *J* = 8.4 Hz, ArH, 2H), 7.67 (d, *J* = 9.0 Hz, ArH, 2H), 7.25 (br s, ArH, 2H), 7.11 (br s, ArH, 2H), 7.00 (m, ArH, 2H), 6.90 (m, ArH, 2H), 4.44 (d, *J* = 13.8 Hz, ArCH_2Ar, 2H), 4.23 (d, *J* = 14.4 Hz, ArCH_2Ar, 2H), 3.52-3.41 (overlapped, ArCH_2Ar + OCH_2(CH_2)$_3$$CH_3$, 8H), 3.33 (s, OC$H_3$, 6H), 1.79 (s, OC$H_3$, 6H), 1.26 (s, *t*-Bu,18H), 1.09-1.11 (overlapped, OCH_2(CH_2)$_3$$CH_3$, 12H), 0.72 (m, OCH_2(CH_2)$_3$CH_3, 6H).

^{13}C-NMR (100 MHz, CD_2Cl_2, 298 K): δ 156.1, 150.8, 150.4, 144.8, 135.7, 134.1, 130.6, 129.6, 128.9, 128.4, 128.1, 126.5, 124.8, 124.8, 123.3, 122.8, 75.1, 60.1, 59.2, 34.4, 31.8, 30.2, 29.3, 23.1, 14.3.

HRMS (FT-ICR MALDI) m/z $[M]^+$ calcd for $C_{58}H_{72}O_6$: 864.5329; found: 864.5318.

Copies of 1D, 2D NMR and HR Mass Spectra

^{1}H NMR, ^{13}C NMR and HR mass spectra of derivative 4

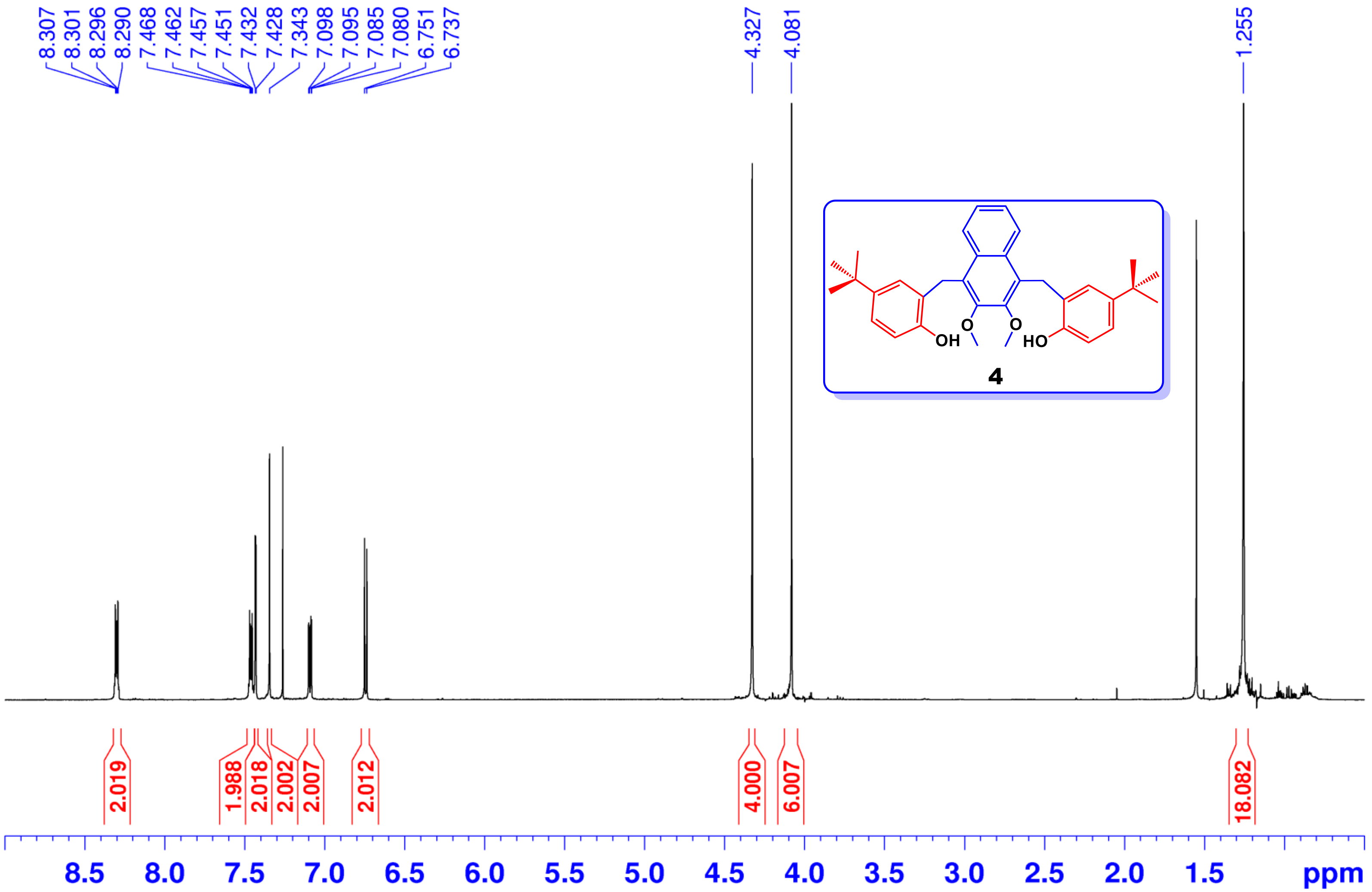

Figure S1: ^{1}H NMR spectrum of **4** ($CDCl_3$, 600 MHz, 298 K).

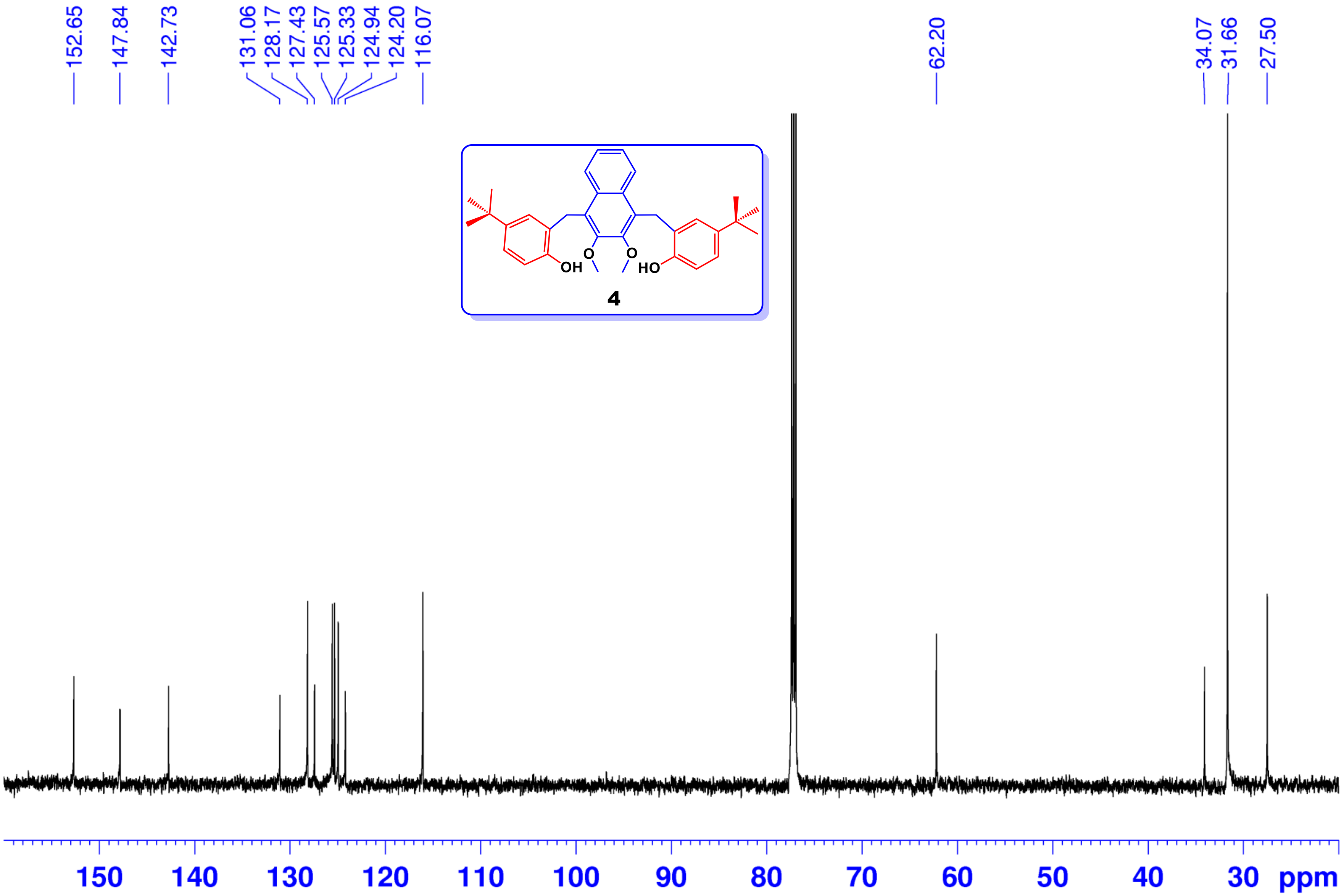

Figure S2: ^{13}C NMR spectrum of **4** ($CDCl_3$, 150 MHz, 298 K).

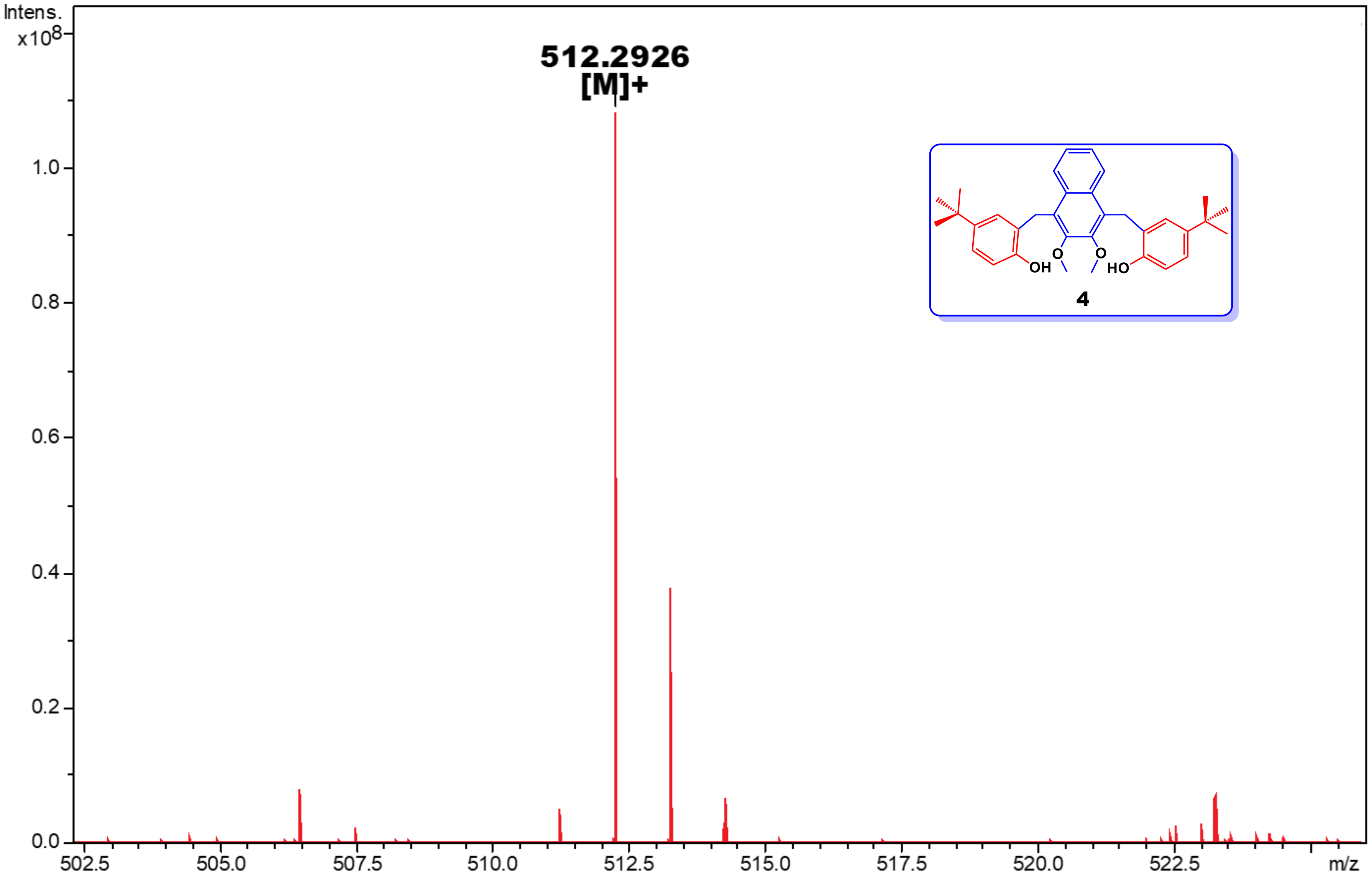

Figure S3: Significant portion of the HR MALDI FT-ICR mass spectrum of **4** $[M]^{+}$.

1D, 2D NMR and HR mass spectra of derivative 1

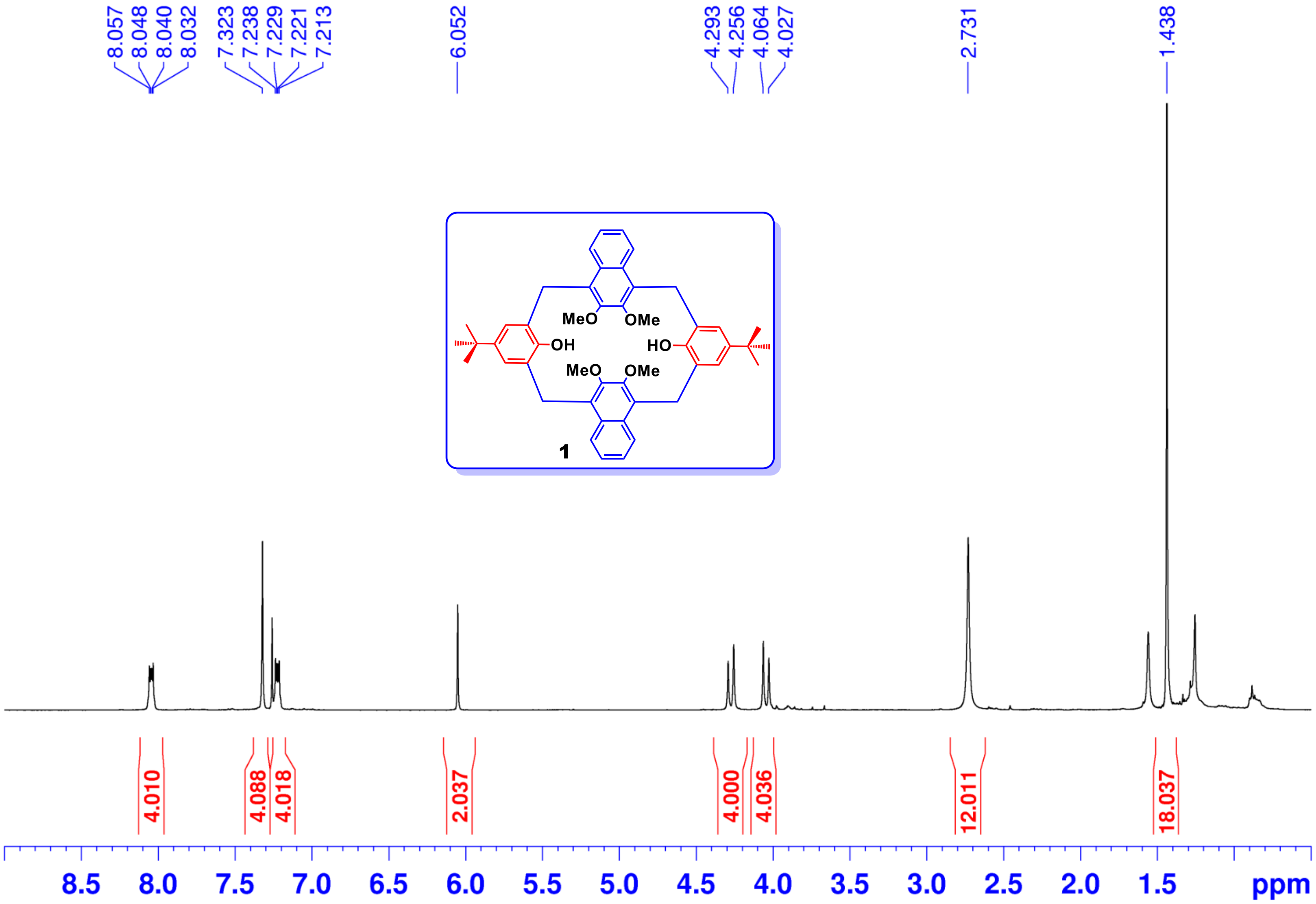

Figure S4: ^{1}H NMR spectrum of **1** ($CDCl_3$, 400 MHz, 298 K).

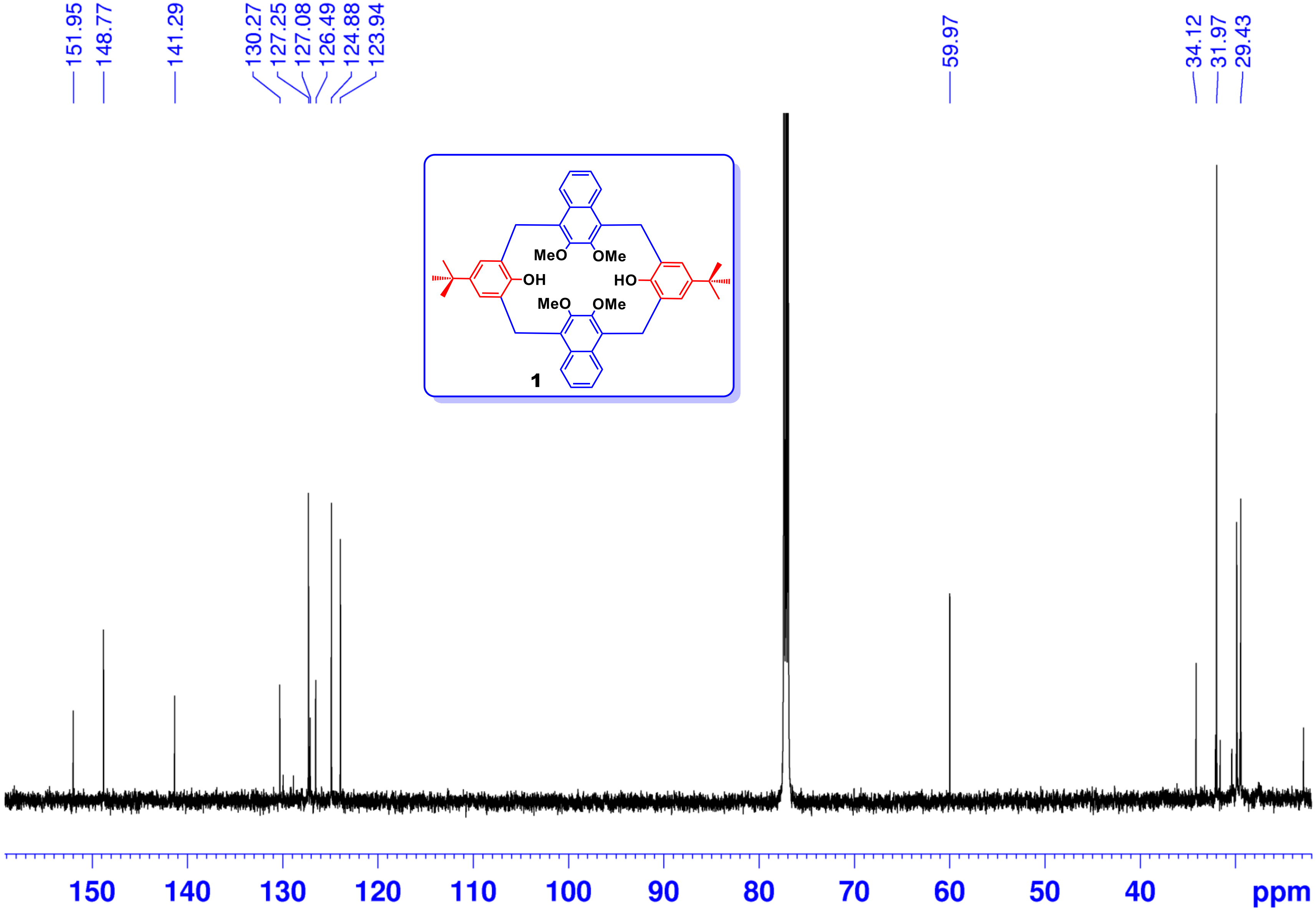

Figure S5: ^{13}C NMR spectrum of **1** ($CDCl_3$, 150 MHz, 298 K).

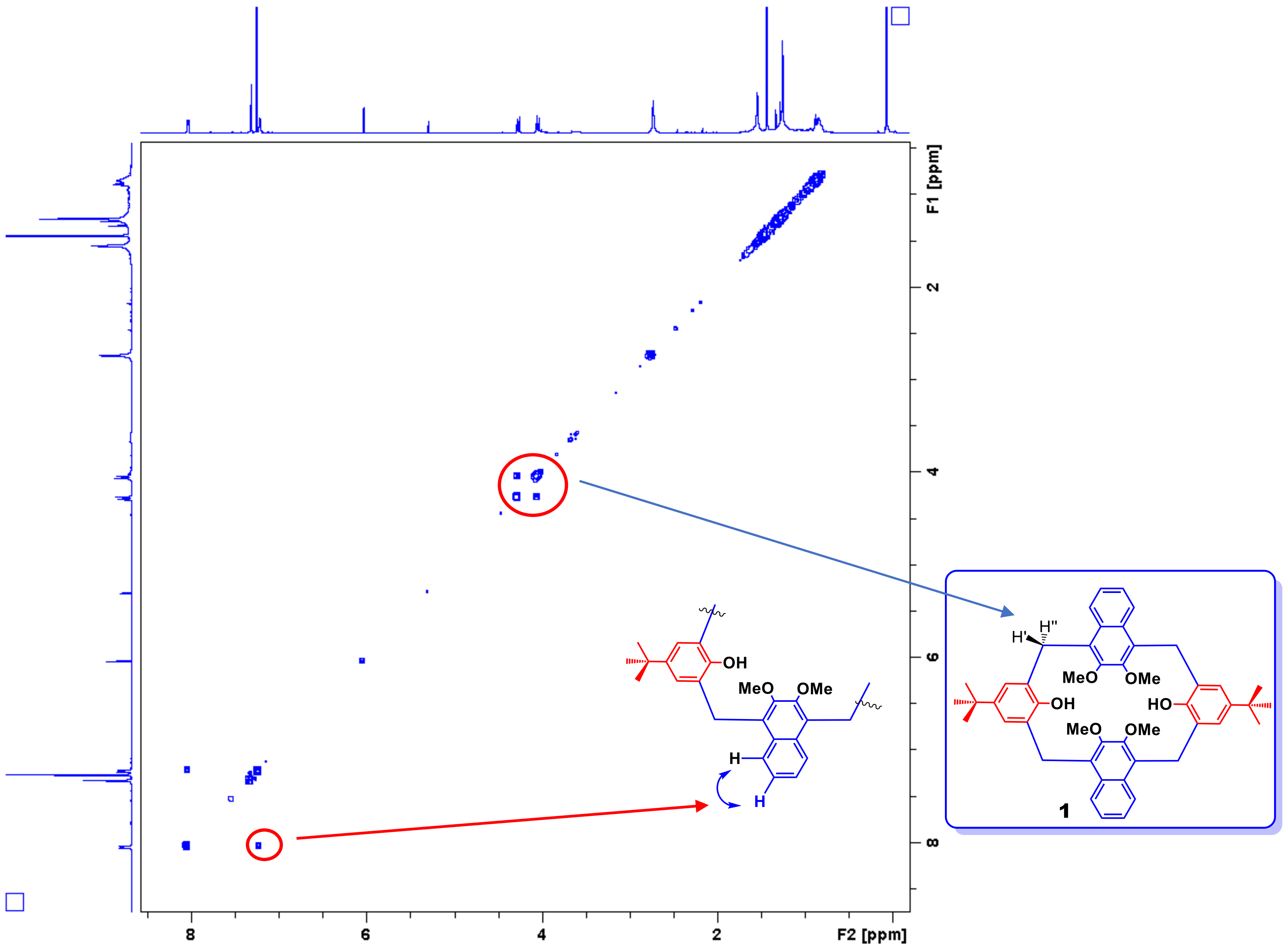

Figure S6: DQF COSY spectrum of **1** ($CDCl_3$, 600 MHz, 298 K).

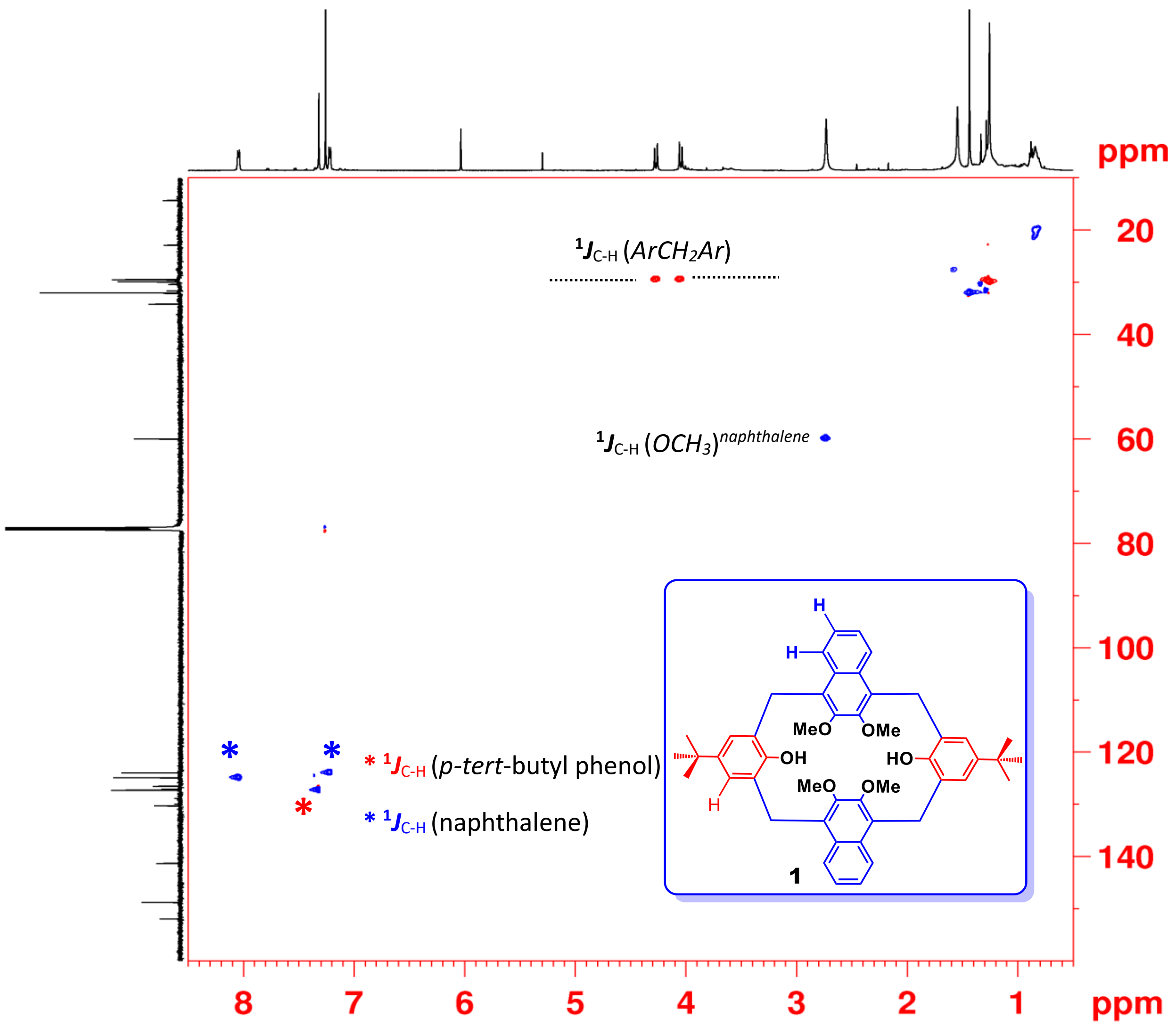

Figure S7: HSQC spectrum of **1** ($CDCl_3$, 600 MHz, 298 K).

VT NMR studies on derivative 1

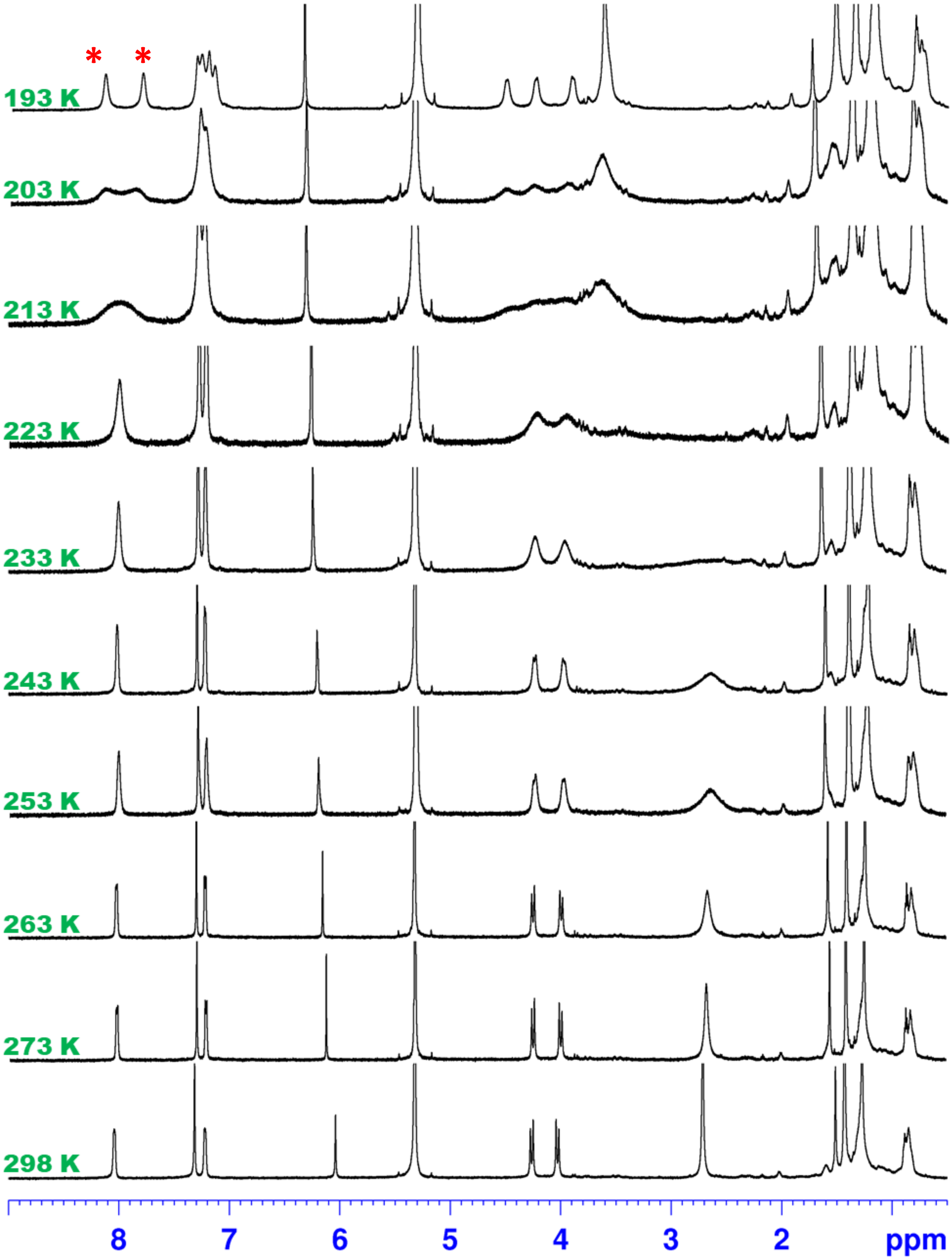

Figure S8: ^{1}H NMR spectrum of **1** (600 MHz, CD_2Cl_2) at (from bottom to top): 298, 273, 263, 253, 243, 233, 223, **213** (Tc), 203 and 193 K. Highlighted in the yellow box the NMR spectrum registered at the coalescence temperature of 213 K.

Energy barrier calculation of 1 by VT NMR studies

$$\Delta G^{\neq} = aTc\left[9.972 + \log\frac{T_c}{\Delta v}\right]$$

Kurland, R. J.; Rubin, M. B.; Wise, M. B. *J. Chem. Phys.* **1964**, *40*, 2426

TC = 213 K; Δv = 204 Hz calculated for naphthalene aromatic protons marked (*) in Figure S8 ;

a = $4.575 \cdot 10^{-3}$ ($\Delta G^{\neq}_c$ in Kcal/mol)

$\Delta G^{\neq}_c$ = 9.7 Kcal/mol

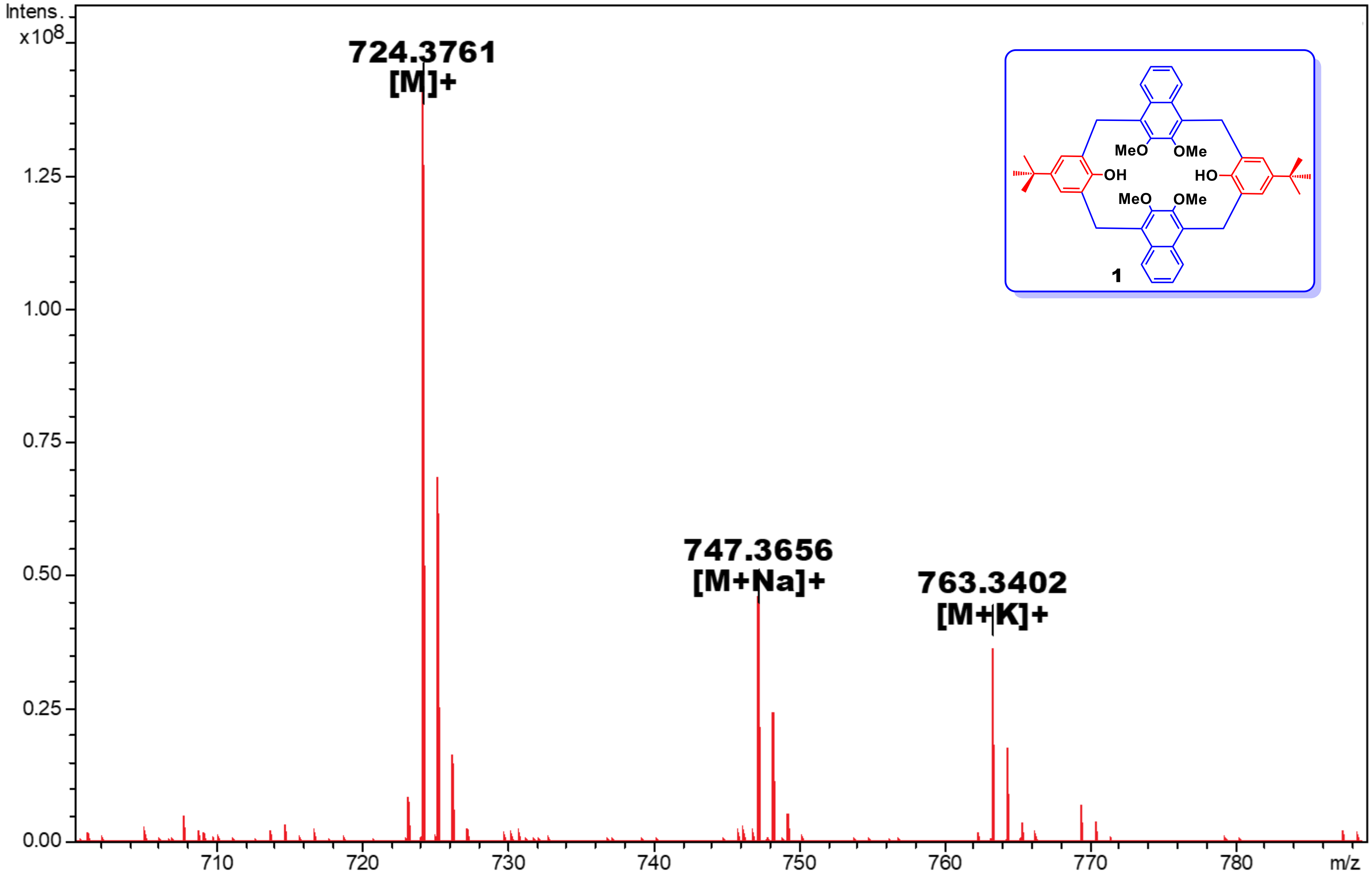

Figure S9: Significant portion of the HR MALDI FT-ICR mass spectrum of **1** $[M]^+$, $[M+Na]^+$ and $[M+K]^+$. Calculated 724.3764, for $C_{48}H_{52}O_6$.

1D, 2D NMR studies and HR mass spectrum of derivative 5

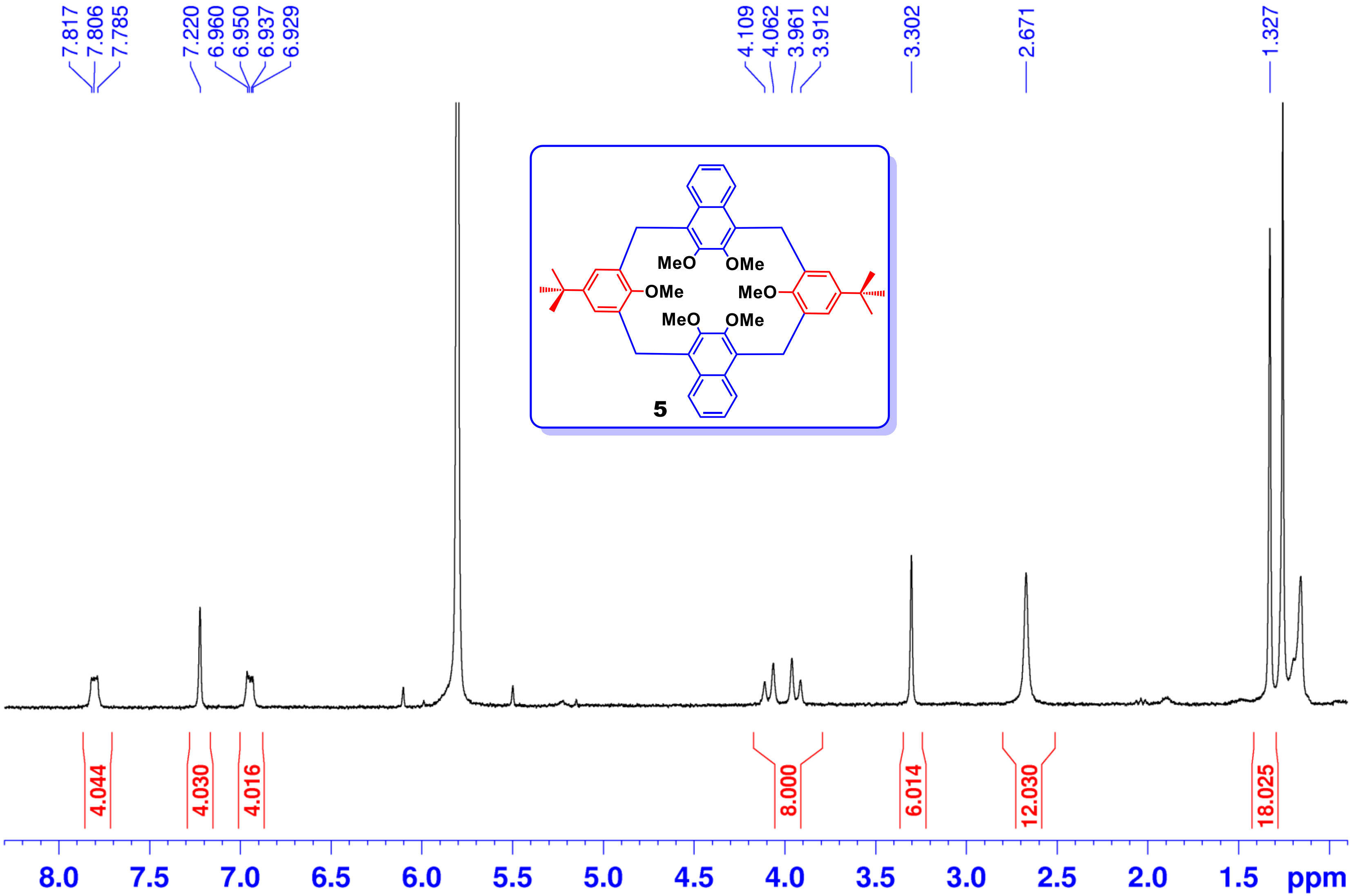

Figure S10: ^{1}H NMR spectrum of **5** (TCDE, 300 MHz, 373 K).

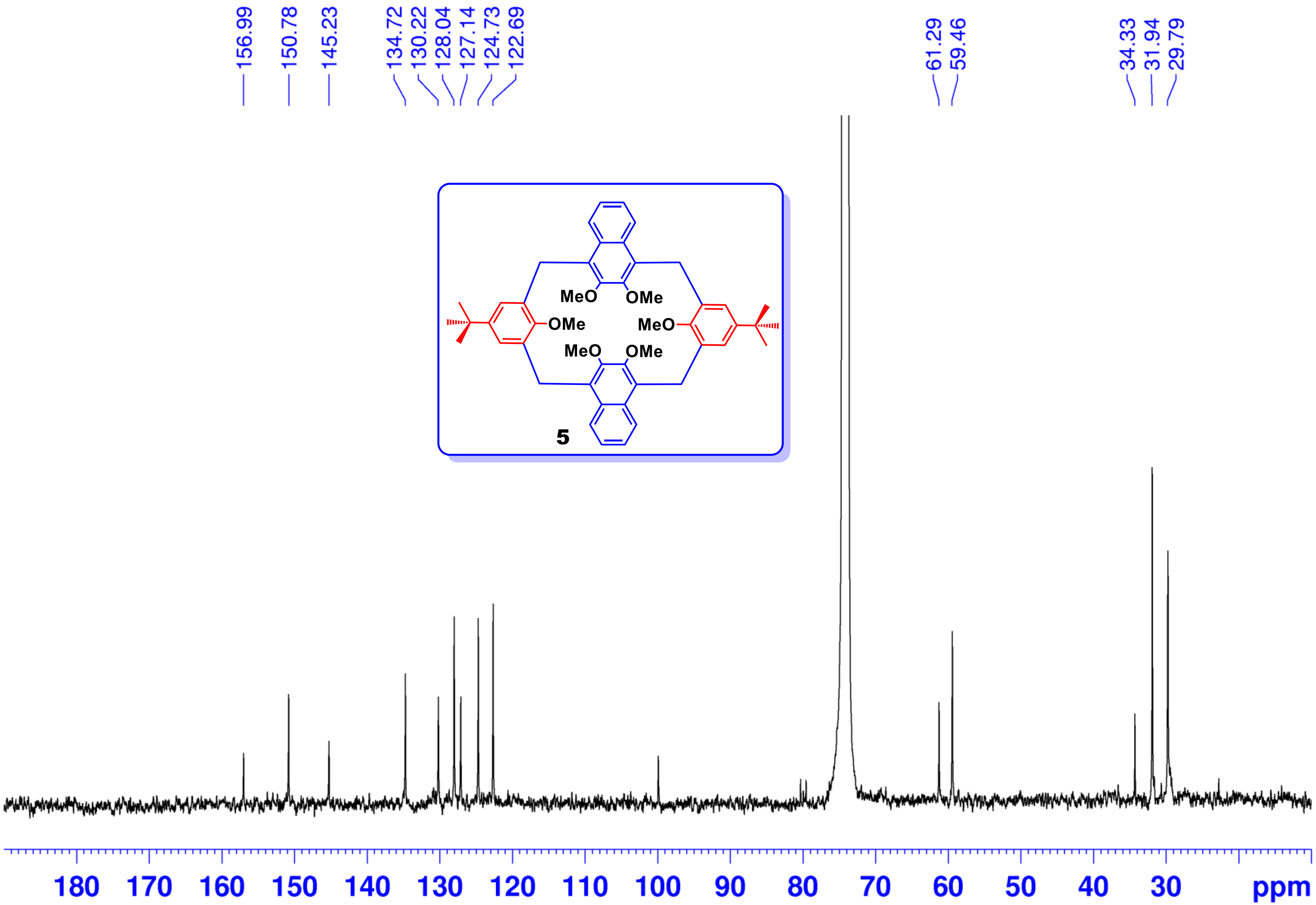

Figure S11: ^{13}C NMR spectrum of **5** (TCDE, 75 MHz, 373 K).

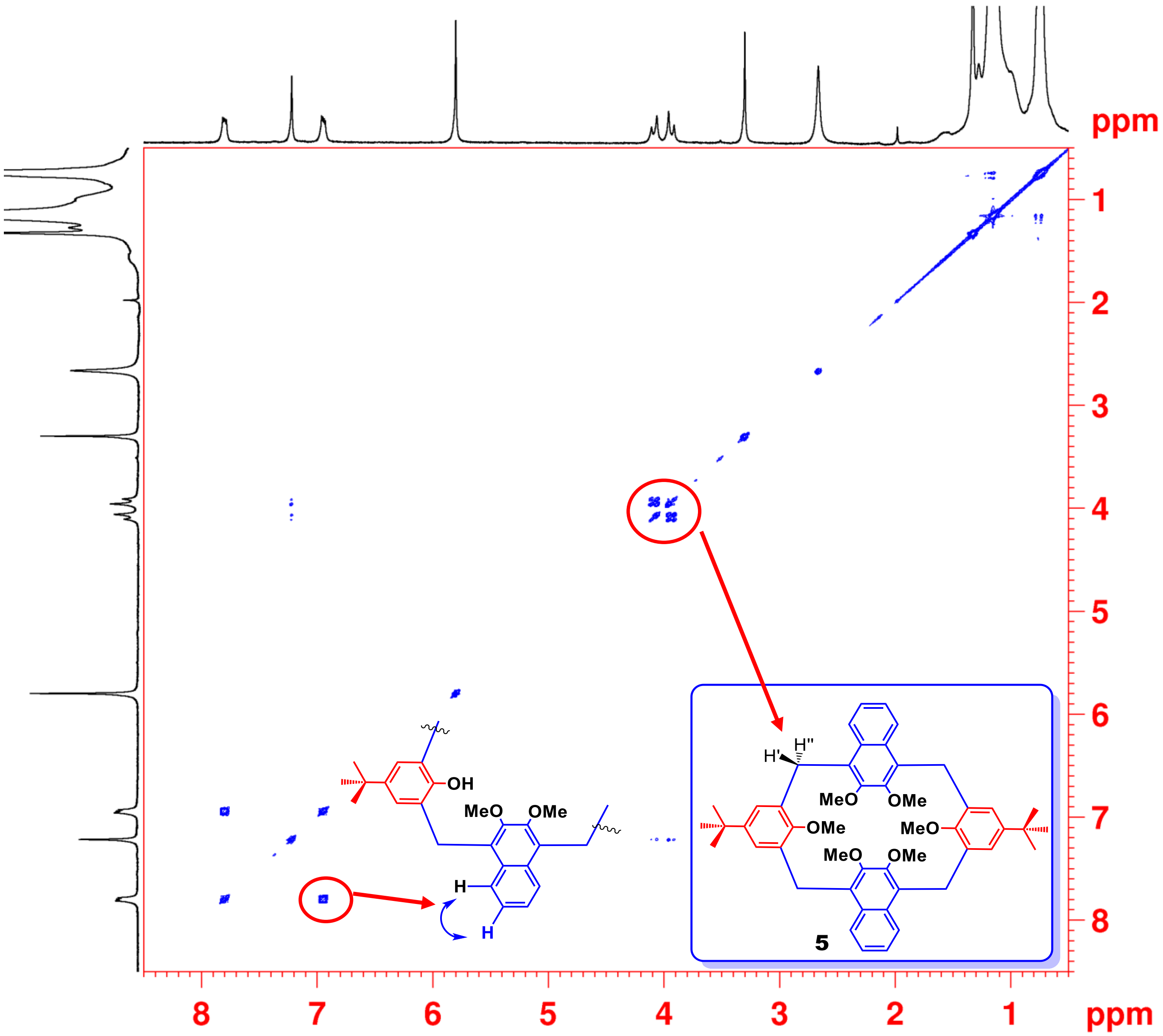

Figure S12: 2D-DQF COSY spectrum of **5** (TCDE, 300 MHz, 373 K).

VT NMR studies of derivative 5

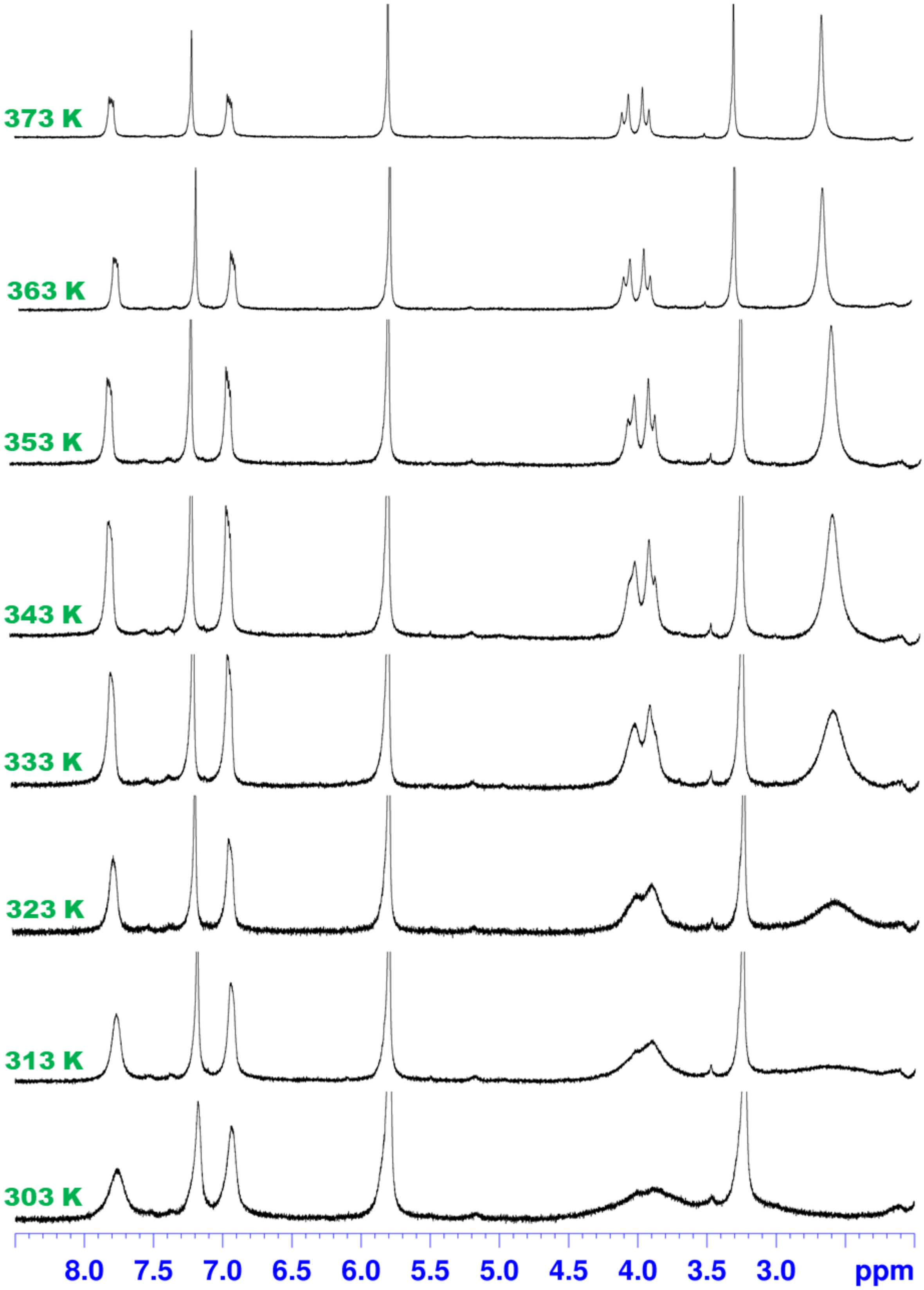

Figure S13: Relevant region of the ^{1}H NMR spectrum of **5** (300 MHz, TCDE) at (from bottom to top): 303, 313, 323, 333, 343, 353, 363 and 373 K.

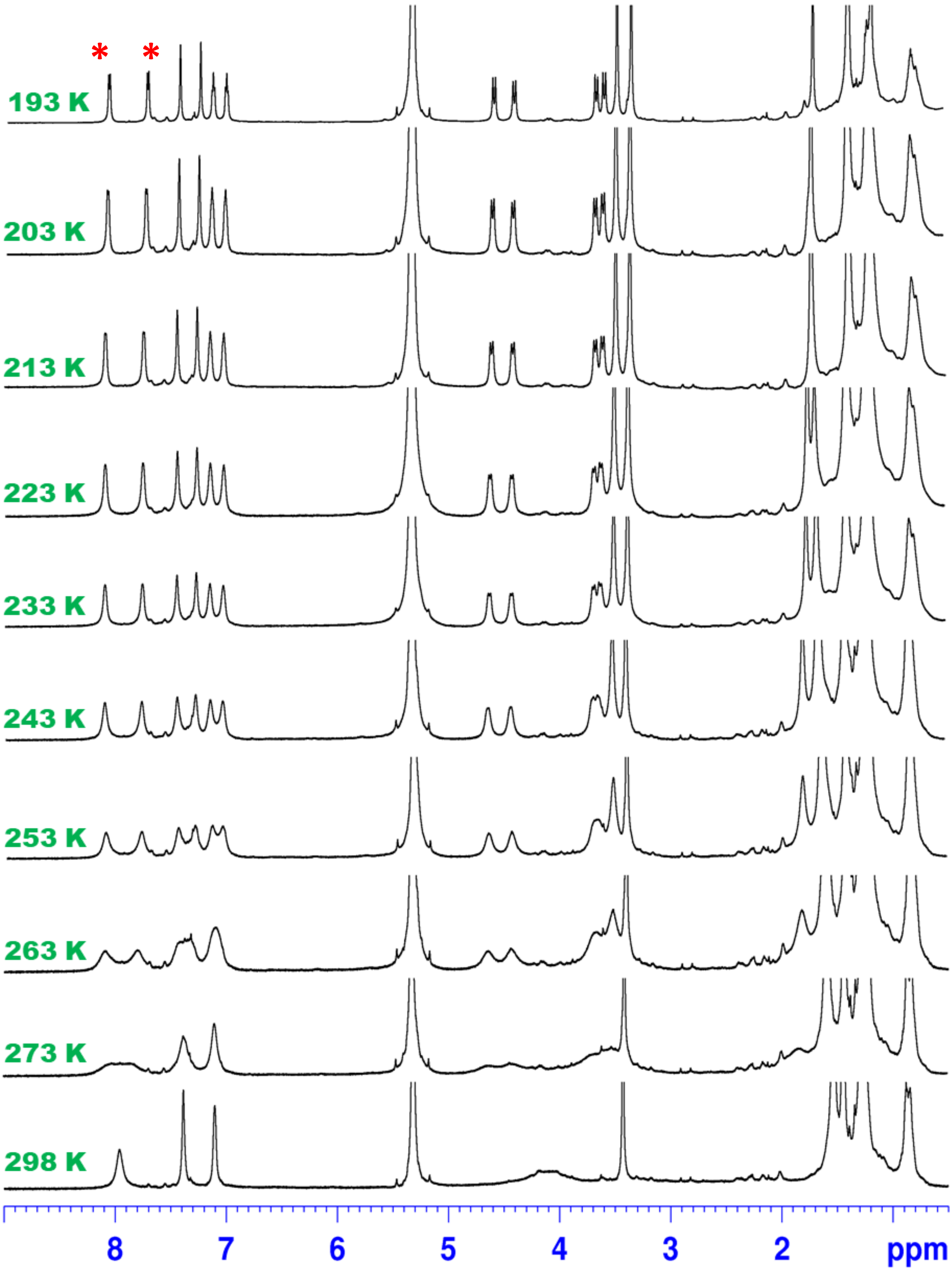

Figure S14: ^{1}H NMR spectrum of **5** (600 MHz, CD_2Cl_2) at (from bottom to top): 298, **273 (Tc)**, 263, 253, 243, 233, 223, 213, 203 and 193 K.

Energy barrier calculation of 5 by VT NMR studies

$$\Delta G^{\neq} = aTc\left[9.972 + \log\frac{T_c}{\Delta v}\right]$$

Kurland, R. J.; Rubin, M. B.; Wise, M. B. *J. Chem. Phys.* **1964**, *40*, 2426.

TC = 273 K; Δv = 209 Hz calculated for naphthalene aromatic protons marked (*) in Figure S19;

a = $4.575 \cdot 10^{-3}$ ($\Delta G^{\neq}_c$ in Kcal/mol)

$\Delta G^{\neq}_c$ = 12.3 Kcal/mol

2D NMR Studies of the calix[2]naphtha[2]arene **5** (Figures S15-S18)

1D and 2D NMR studies of **5** at 193 K show the presence of two AX systems (COSY) attributable to the aromatic naphthalene H-atoms at 8.09/7.12 and 7.73/7.02 ppm, while an AB system at 7.25/7.43 ppm was attributable to the anisole rings. In addition, two AX systems (COSY) at 4.59/3.64 and 4.40/3.58 ppm were detected, attributable to the methylene bridges. By NOESY and HSQC experiments the two singlets at 3.48 and 1.70 ppm were attributed to OMe groups of the naphthalene rings, while the singlet at 3.33 ppm was attributed to that of the anisole rings.

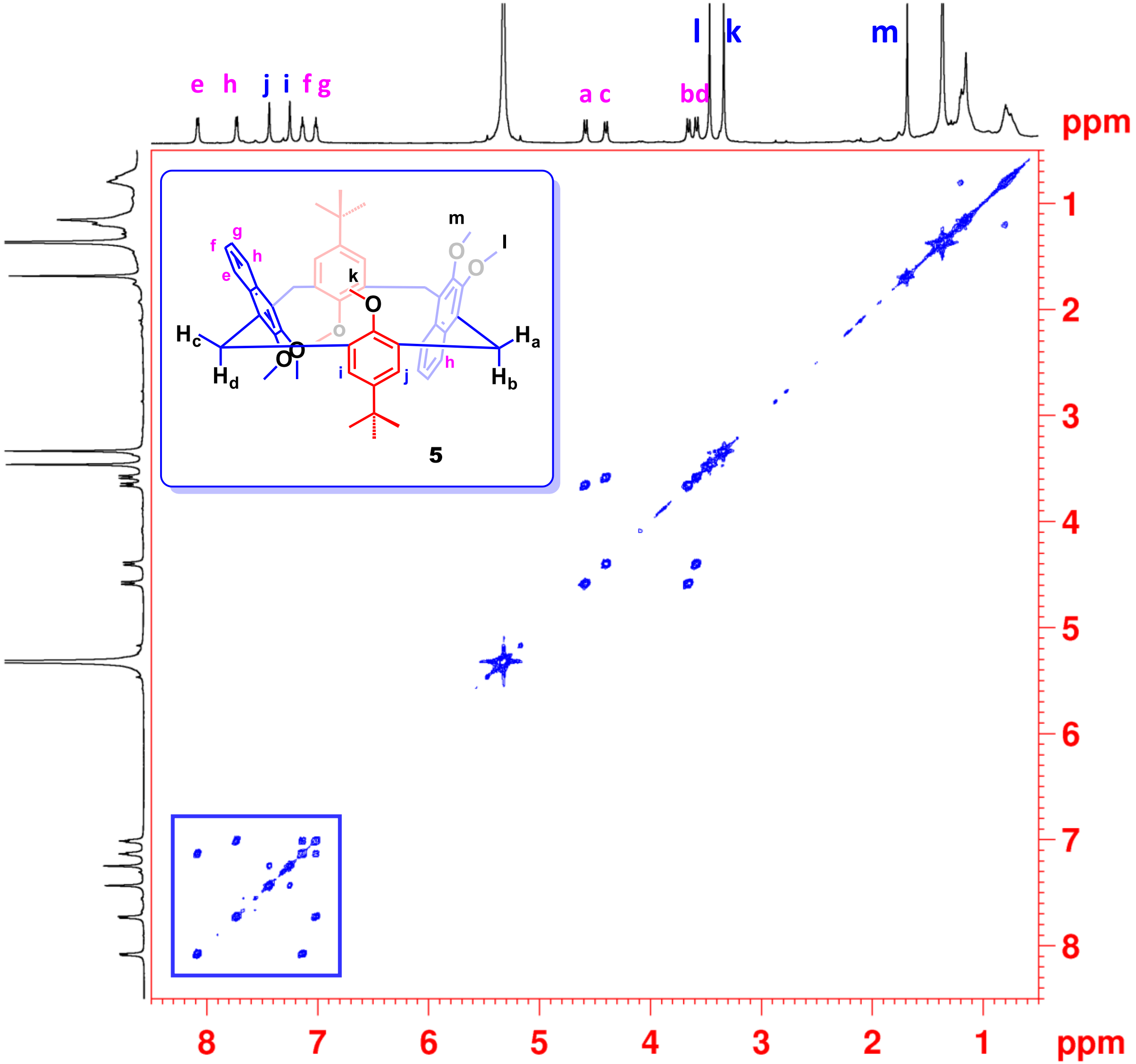

Low temperature 2D NMR studies for 5

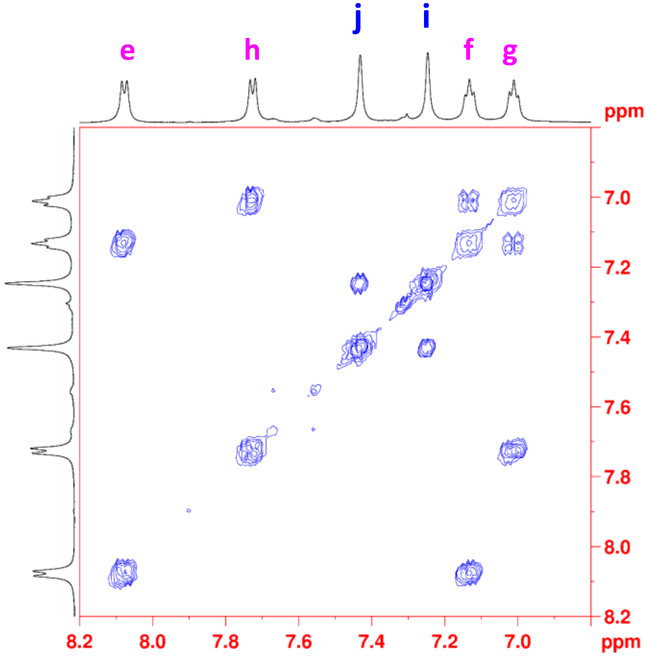

Figure S15: 2D-DQF COSY spectrum of **5** (CD_2Cl_2, 600 MHz, 193 K).

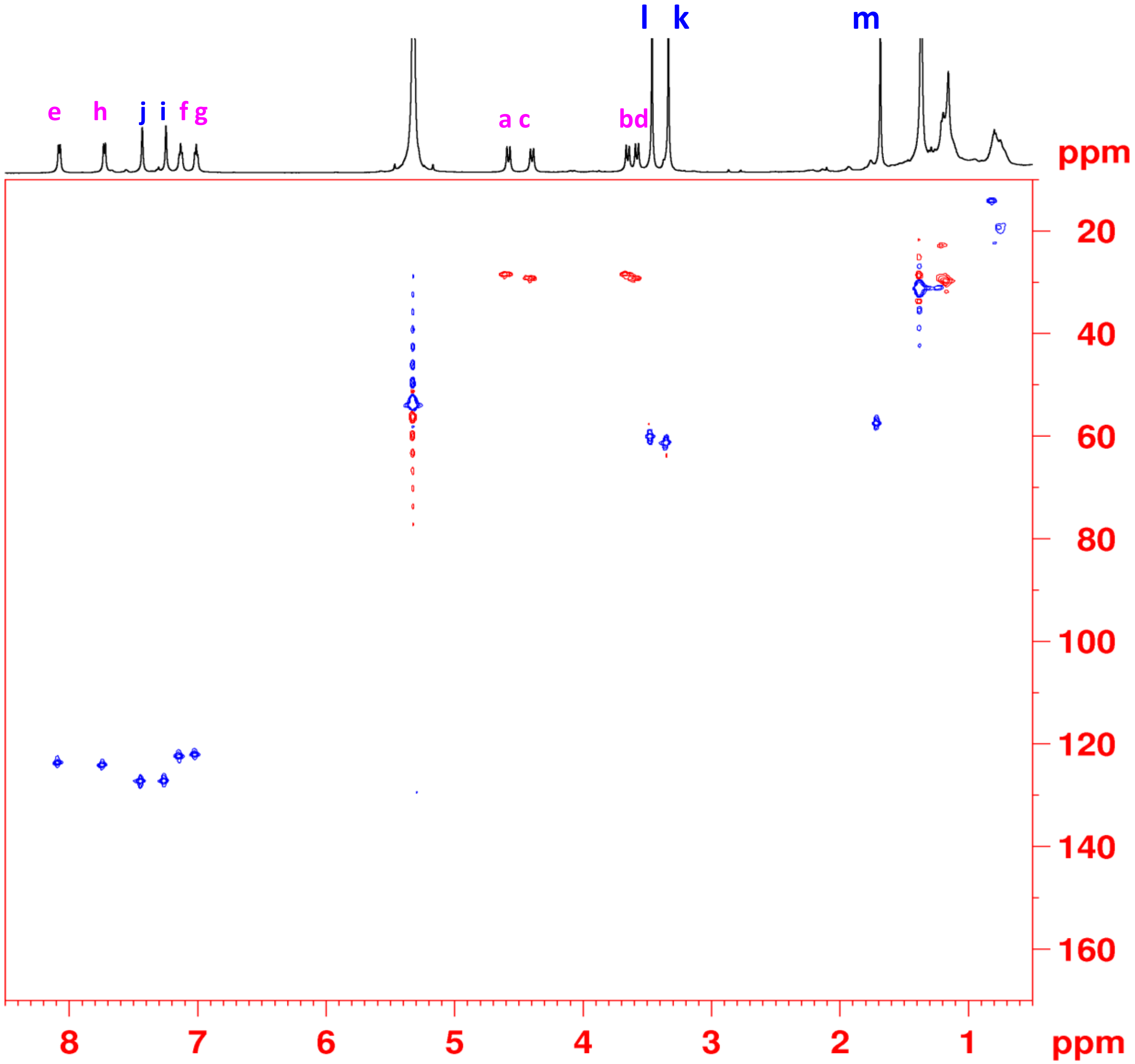

Figure S16: 2D-HSQC spectrum of **5** (CD_2Cl_2, 600 MHz, 193 K).

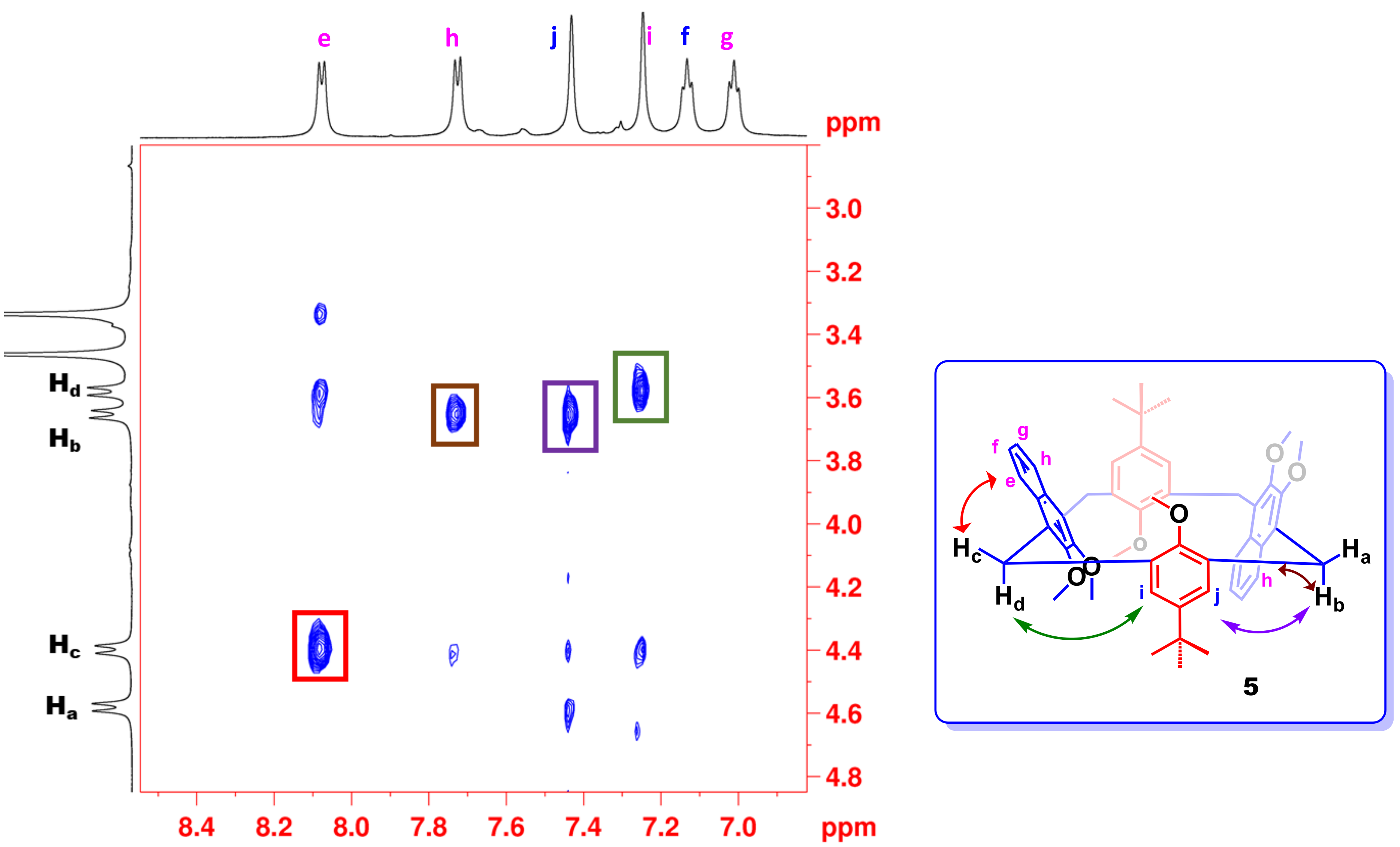

Figure S17: Significant portion of the NOESY spectrum of **5** (CD_2Cl_2, 600 MHz, 193 K).

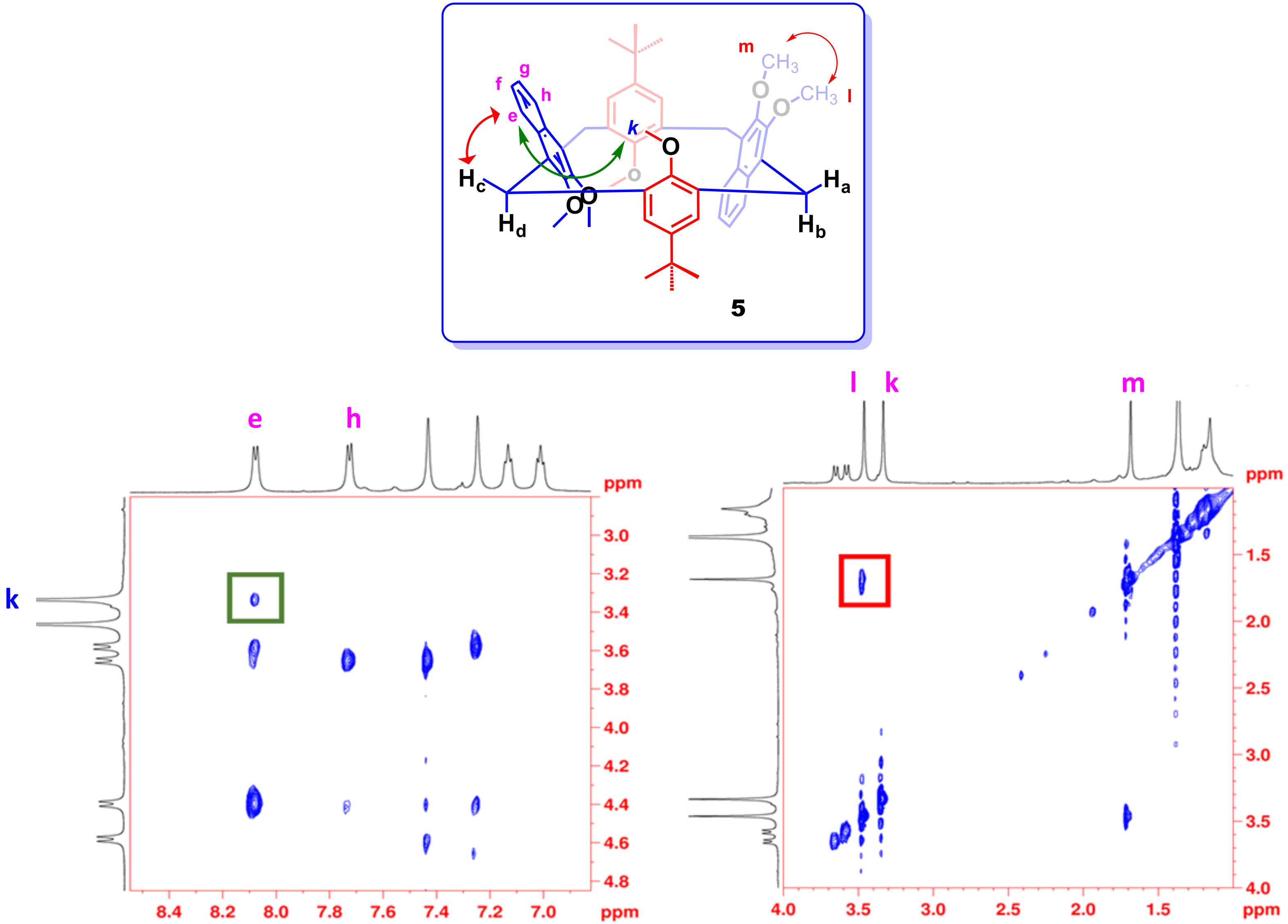

Figure S18: Significant portions of the NOESY spectrum of **5** (CD_2Cl_2, 600 MHz, 193 K).

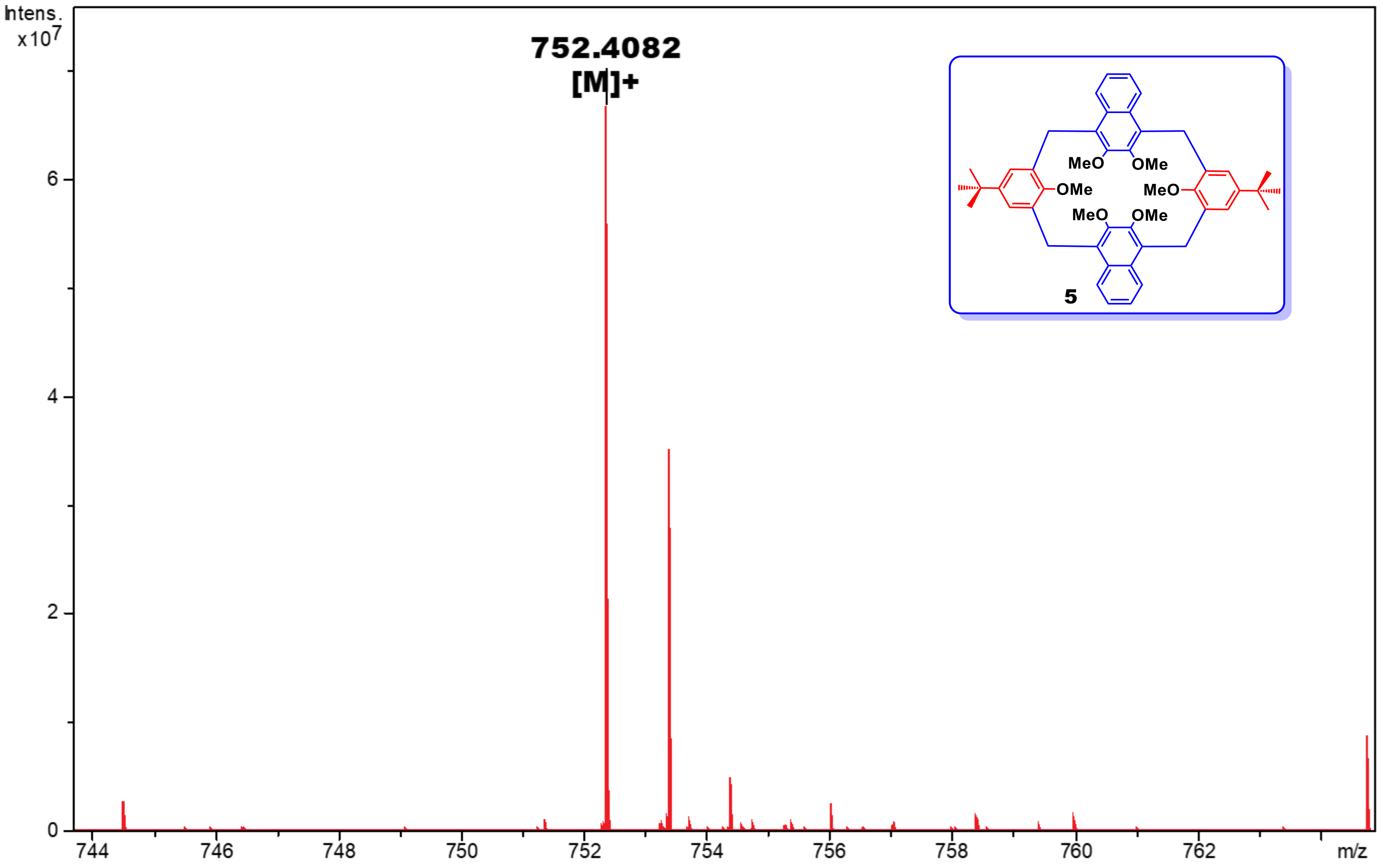

Figure S19: Significant portion of the HR MALDI FT-ICR mass spectrum of **5** $[M]^+$.

1D, 2D and HR mass spectrum of derivative 6

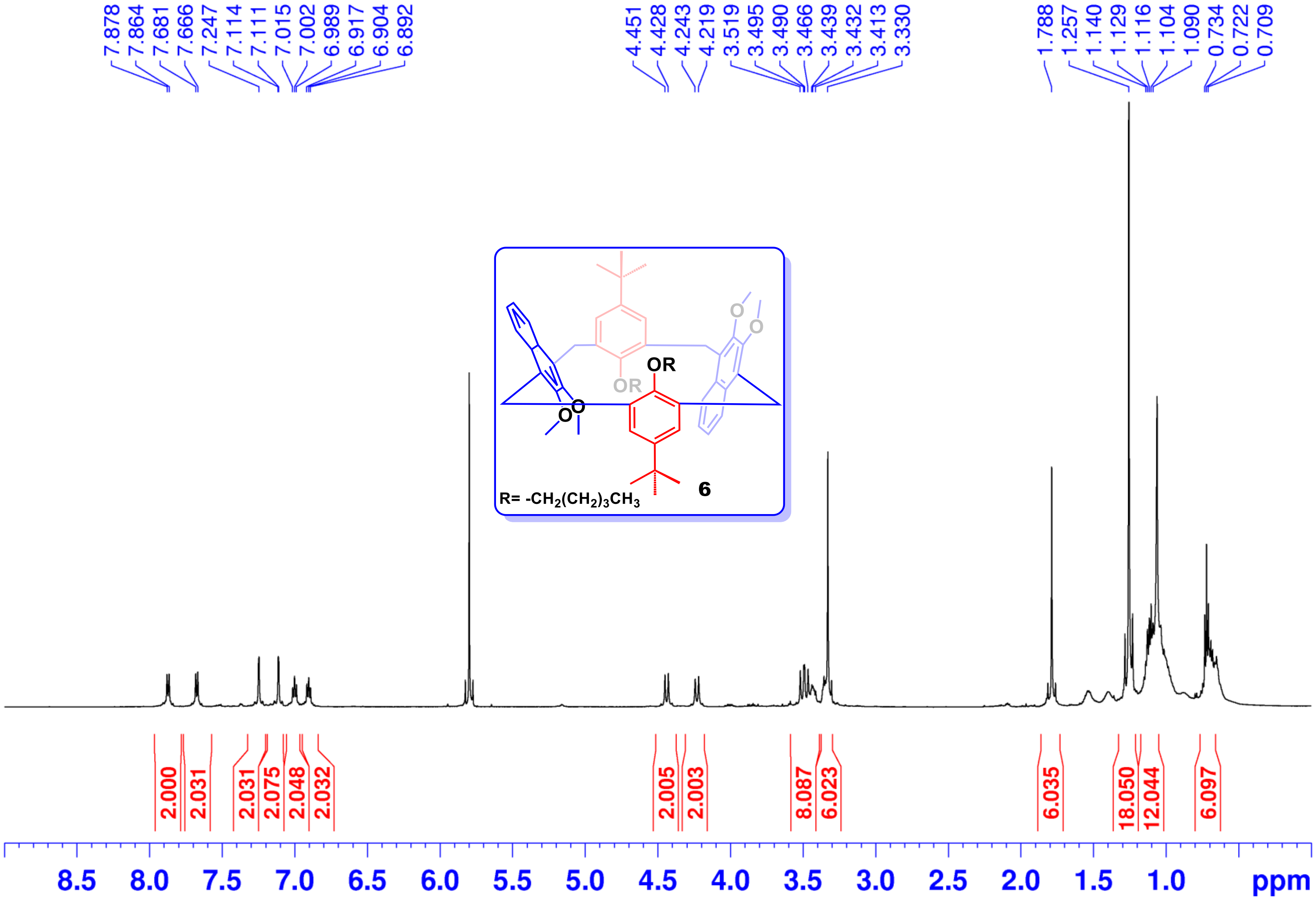

Figure S20: ^{1}H NMR spectrum of **6** (TCDE, 600 MHz, 298 K).

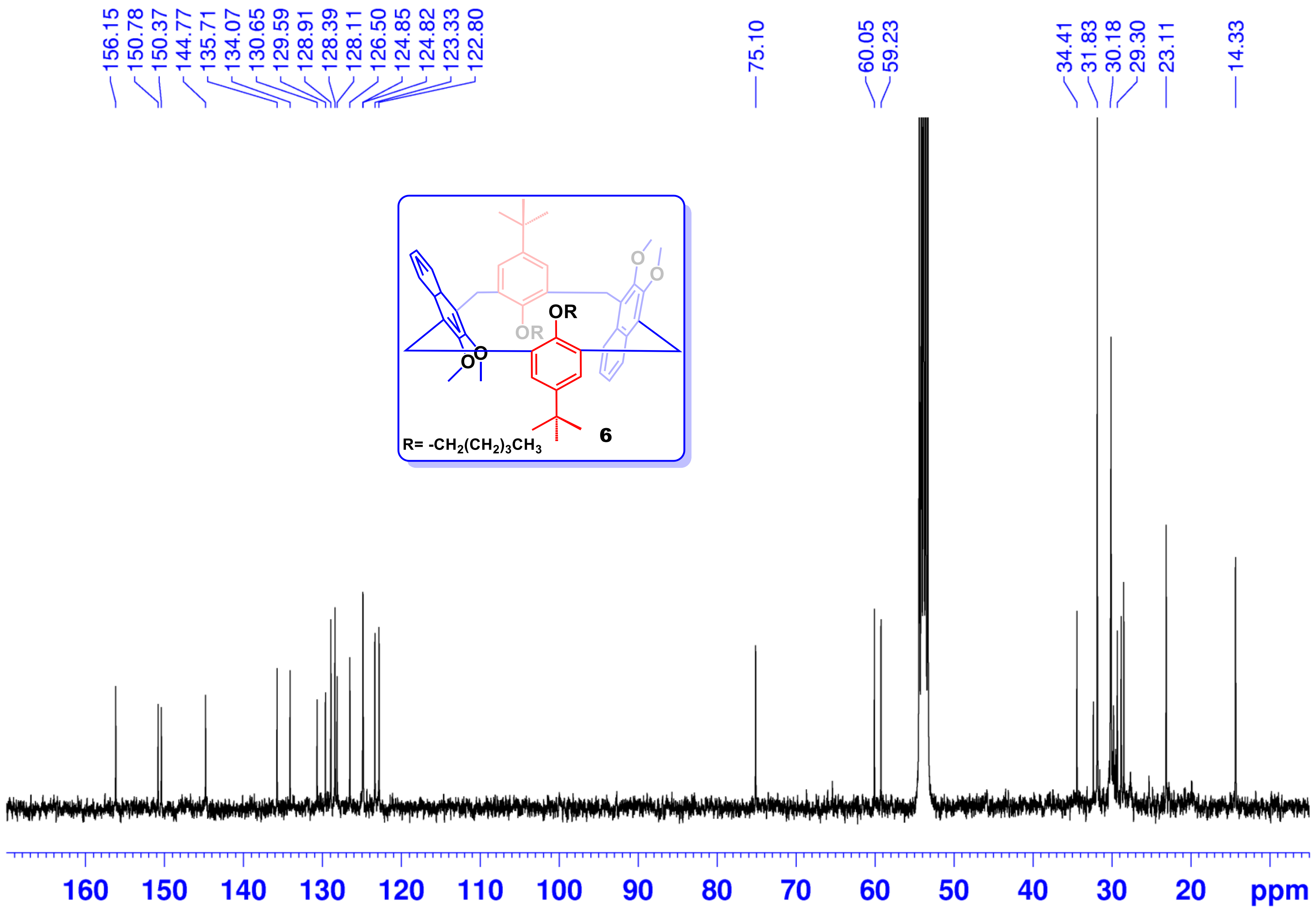

Figure S21: ^{13}C NMR spectrum of **6** (CD_2Cl_2, 100 MHz, 298 K).

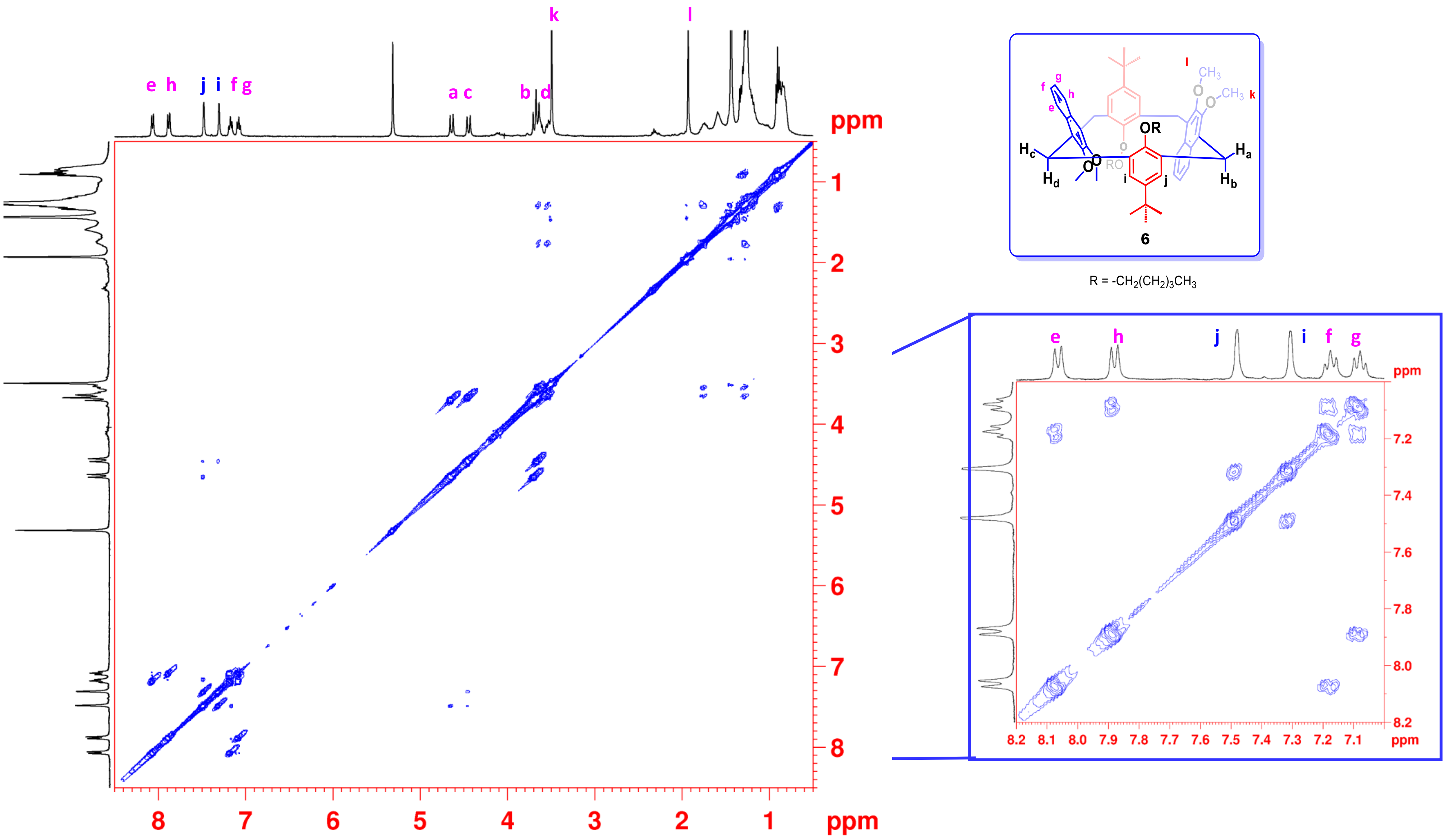

Figure S22: 2D-DQF COSY spectrum of **6** (CD_2Cl_2, 600 MHz, 298 K).

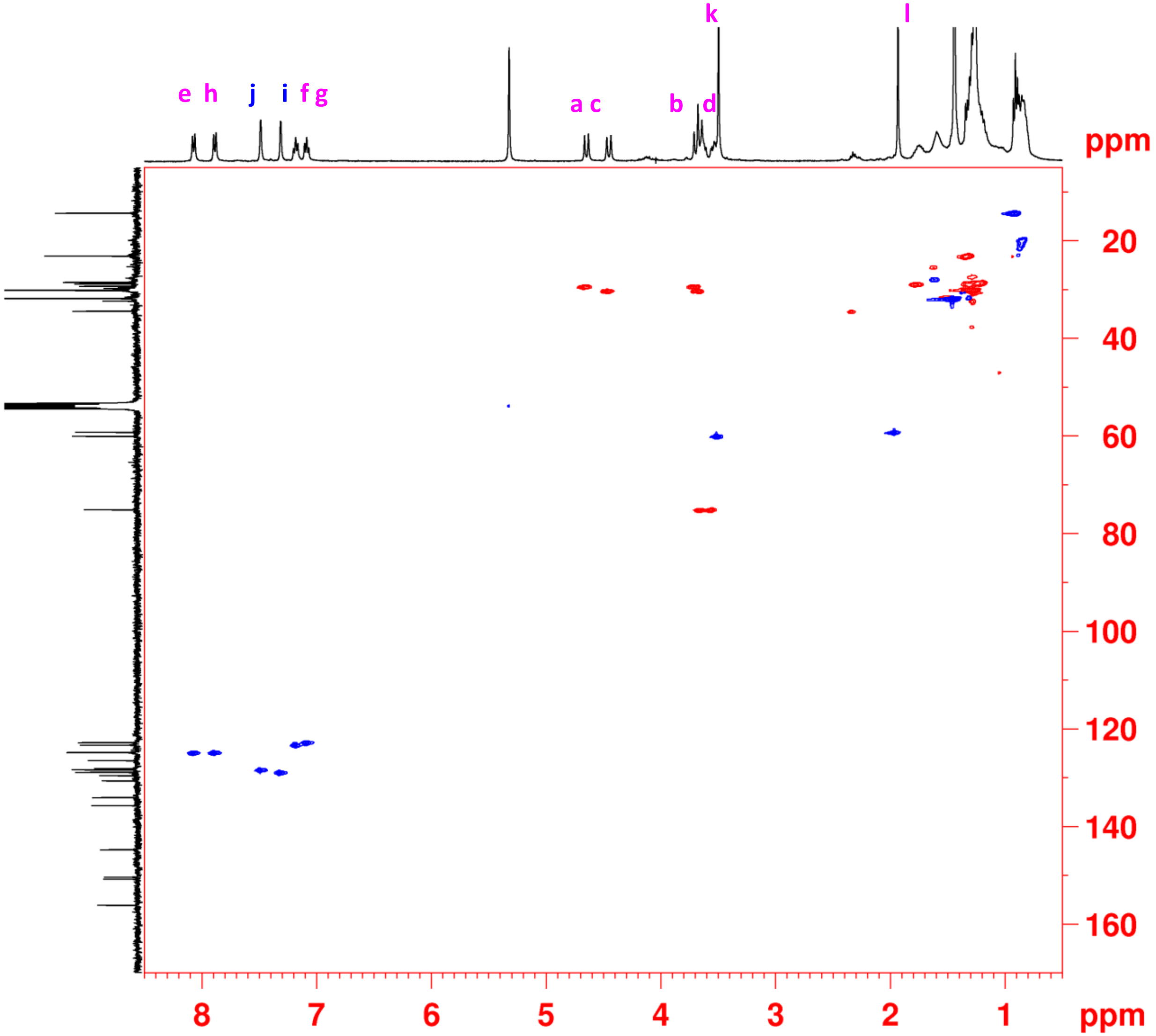

Figure S23: 2D-HSQC spectrum of **6** (CD_2Cl_2, 600 MHz, 298 K).

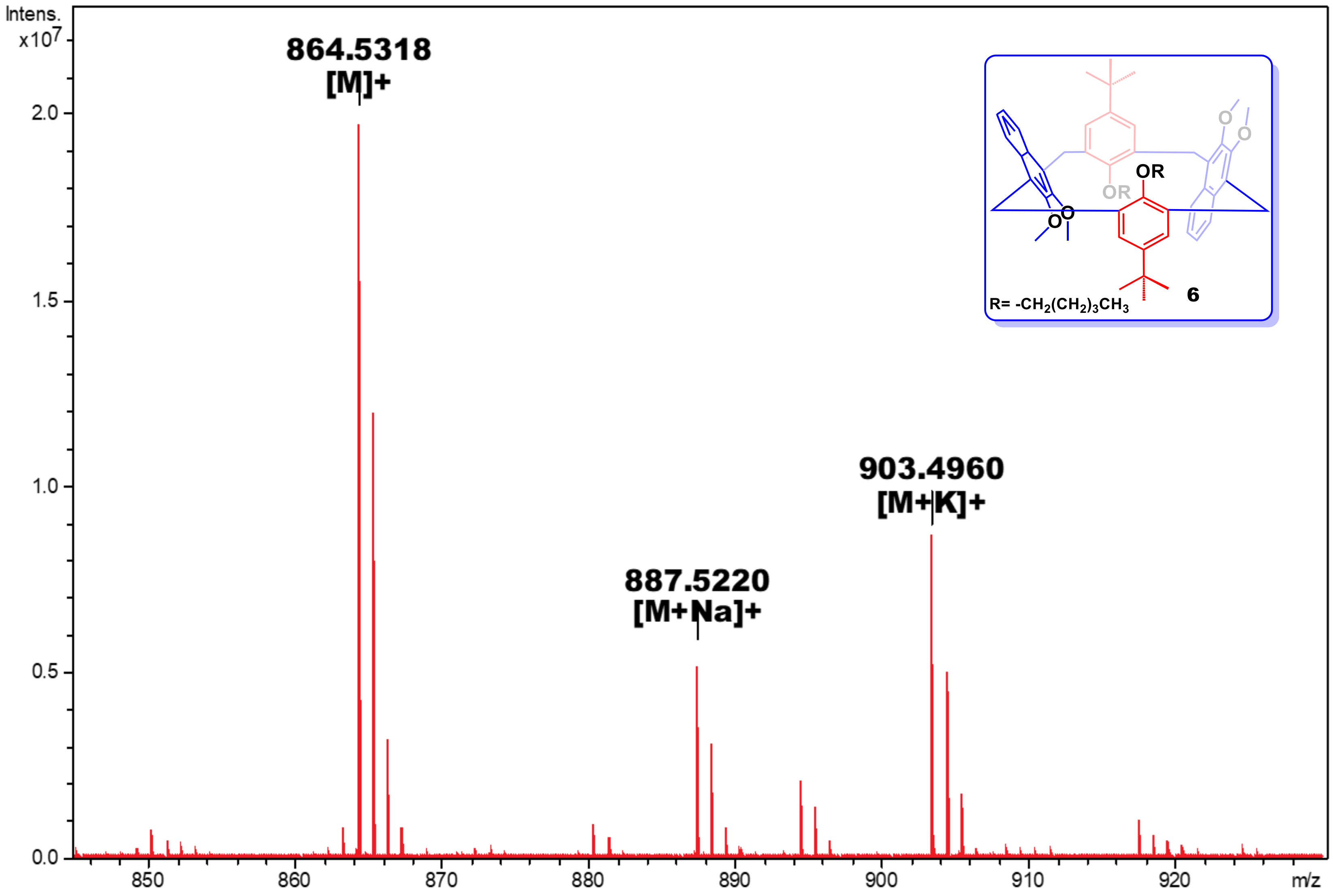

Figure S24: Significant portion of the HR MALDI FT-ICR mass spectrum of **6** $[M]^+$, $[M+Na]^+$ and $[M+K]^+$.

Complexation Studies

The complexes $M^+ \subset$ calix[2]naphth[2]arene were prepared by mixing an equimolar quantity of macrocyclic host and M[B(Ar^F)$_4$] salt in CD_2Cl_2.

Copies of 1D and 2D NMR Spectra of Complexes

Copies of NMR Spectra of $Na^+ \subset$ 5 complex

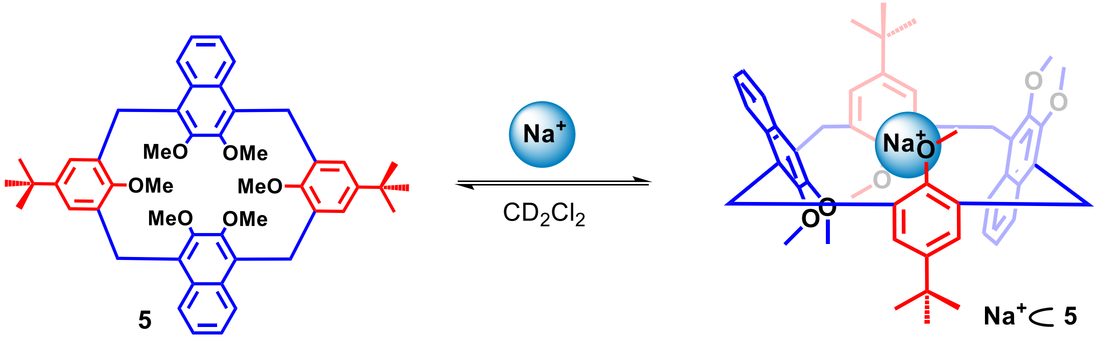

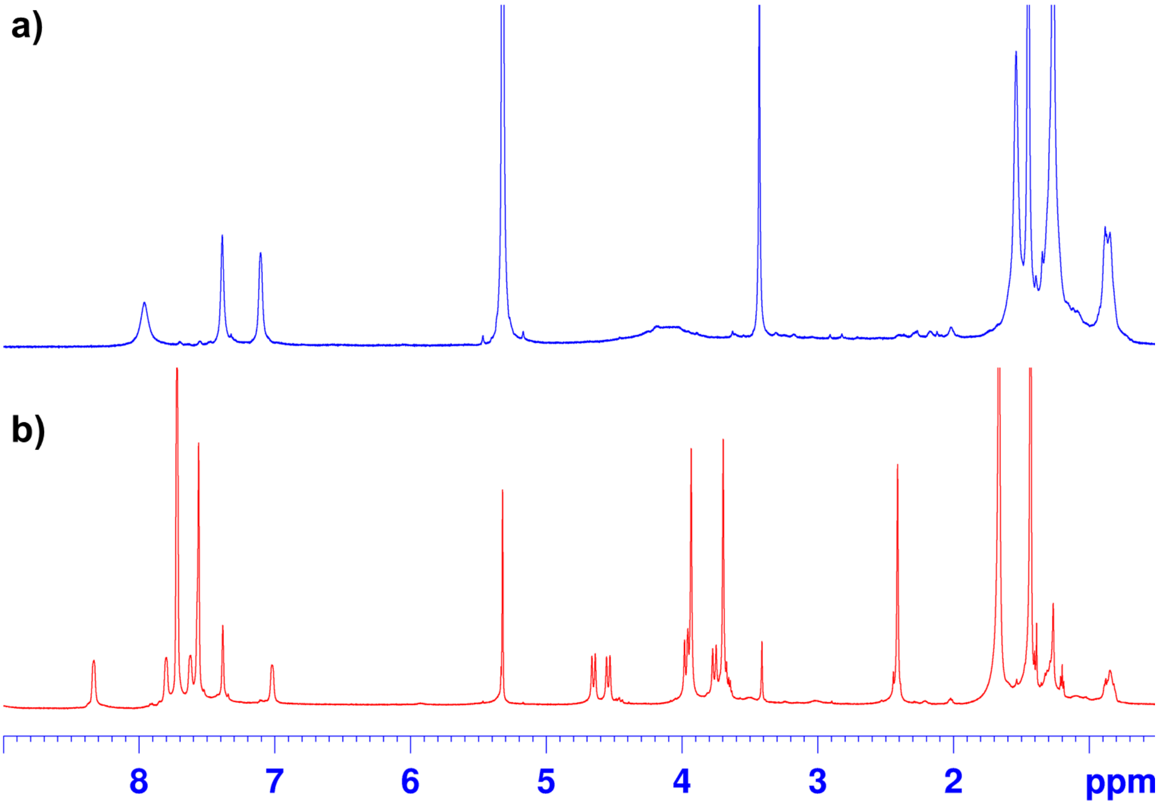

Figure S25: ^{1}H NMR spectra (600 MHz, CD_2Cl_2, 298 K) of: (a) a solution of **5** and (b) an equimolar solution (4.3 mM) of **5** and Na[B(Ar^F)$_4$] in 0.4 mL of CD_2Cl_2.

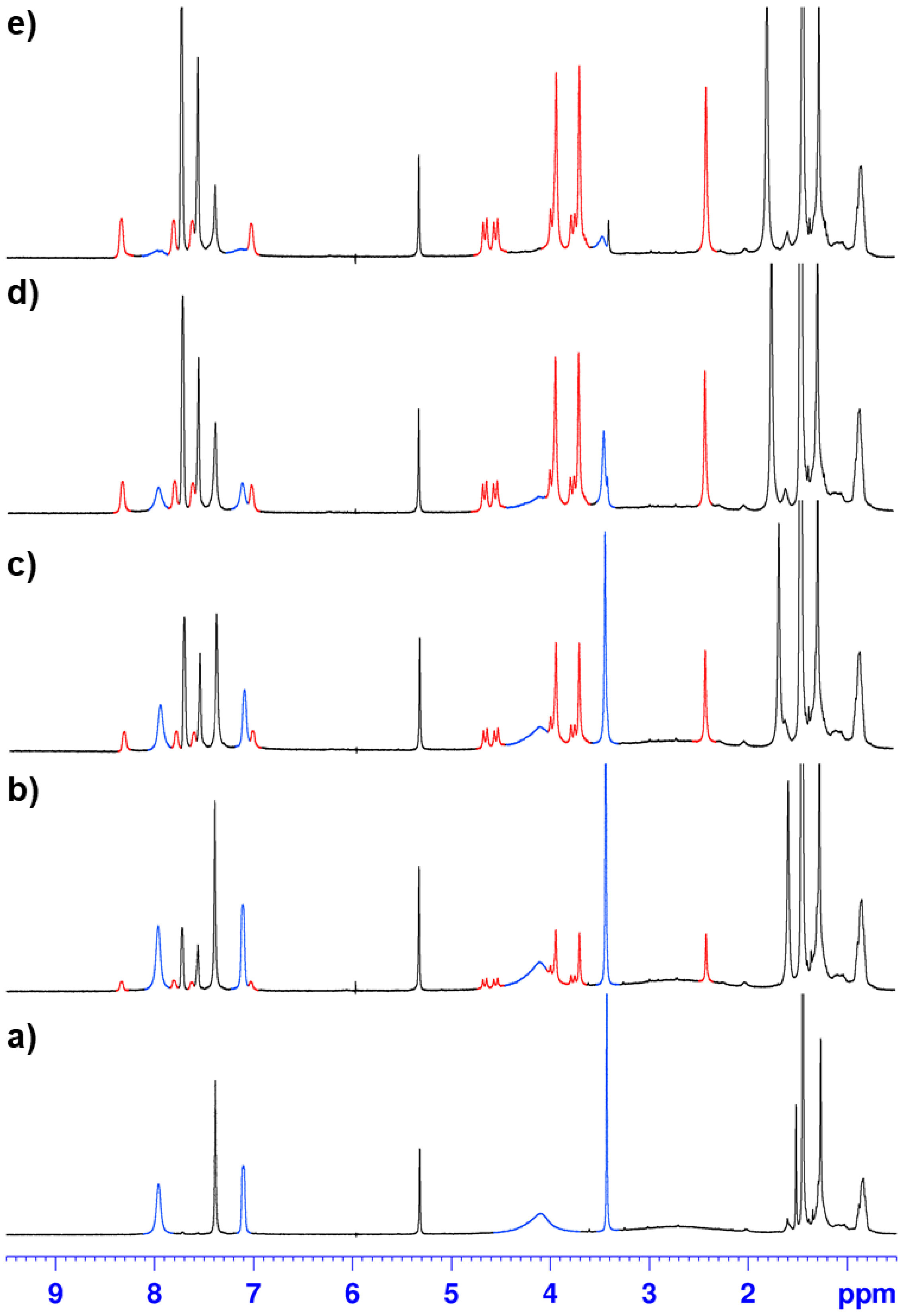

Figure S26: ^{1}H NMR spectrum (400 MHz, CD_2Cl_2, 298 K) of: (a) **5**; (b) **5** and 0.25 equiv of Na[B(Ar^F)$_4$]; (c) **5** and 0.50 equiv of Na[B(Ar^F)$_4$]; (d) **5** and 0.75 equiv of Na[B(Ar^F)$_4$]; (e) **5** and 1 equiv of Na[B(Ar^F)$_4$]; In **red** the signals of the complex **Na$^+$$\subset$5** and in **blue** the signals of **5**.

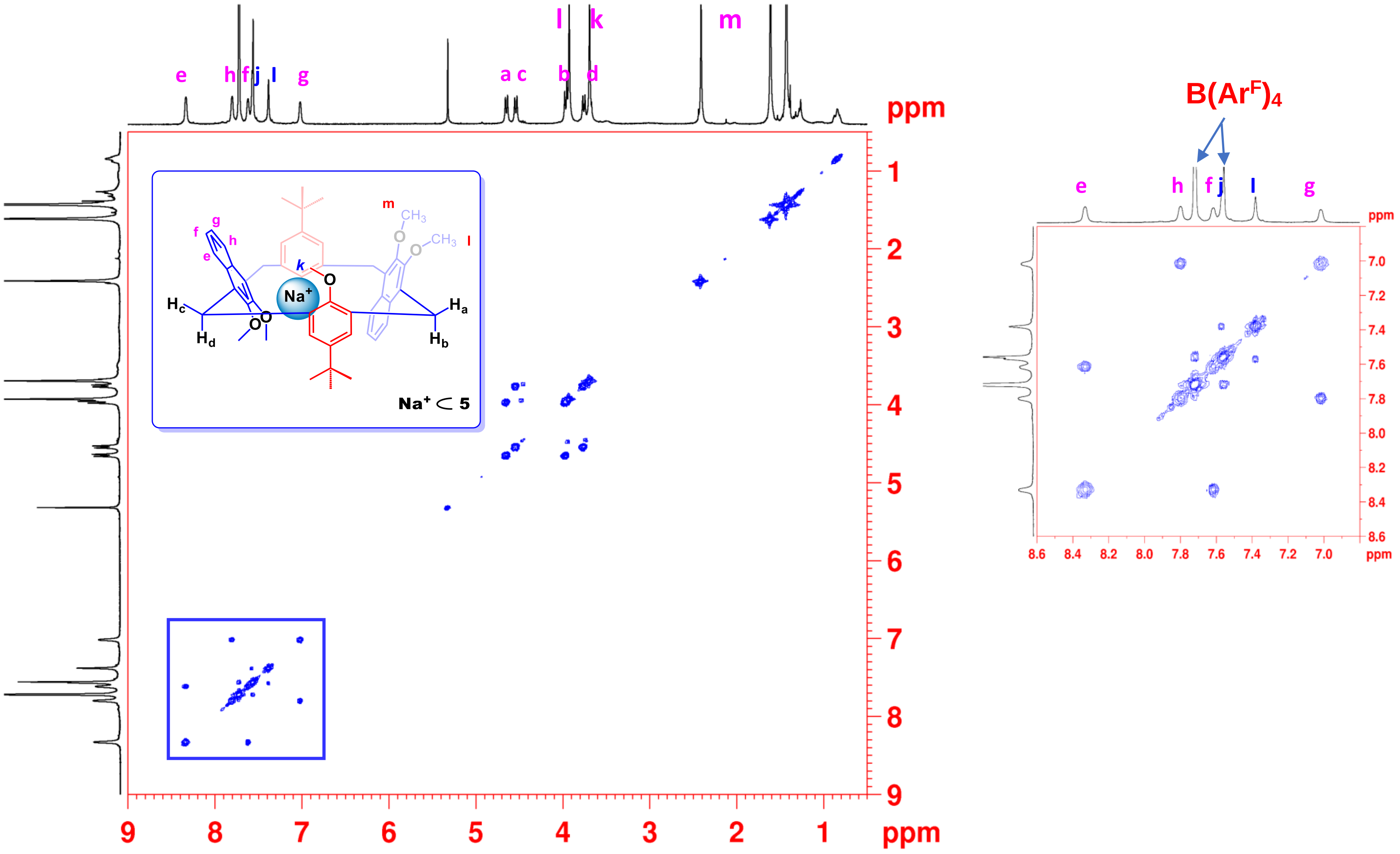

Figure S27: 2D-DQF COSY spectrum of **Na⁺⊂5** (CD_2Cl_2, 600 MHz, 298 K).

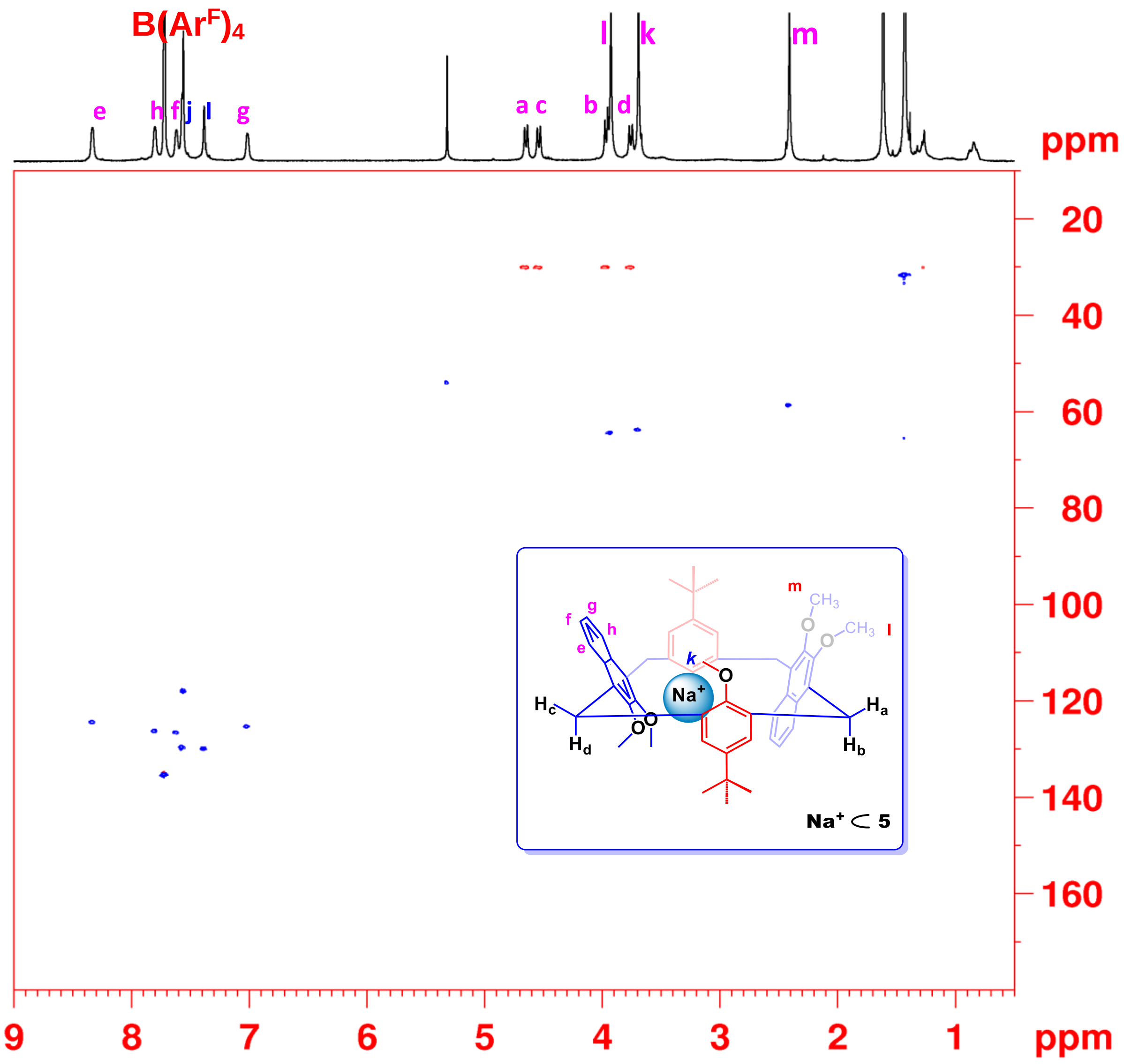

Figure S28: 2D-HSQC spectrum of **$Na^+ \subset$ 5** (CD_2Cl_2, 600 MHz, 298 K).

Copies of NMR Spectra of $K^+ \subset 5$

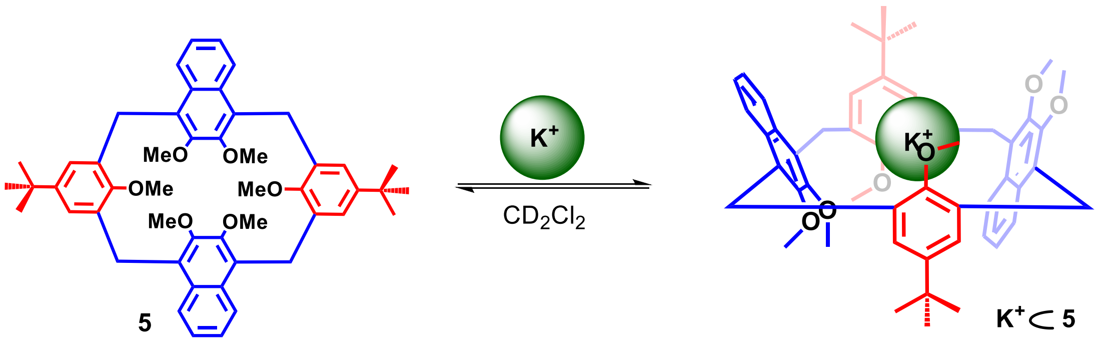

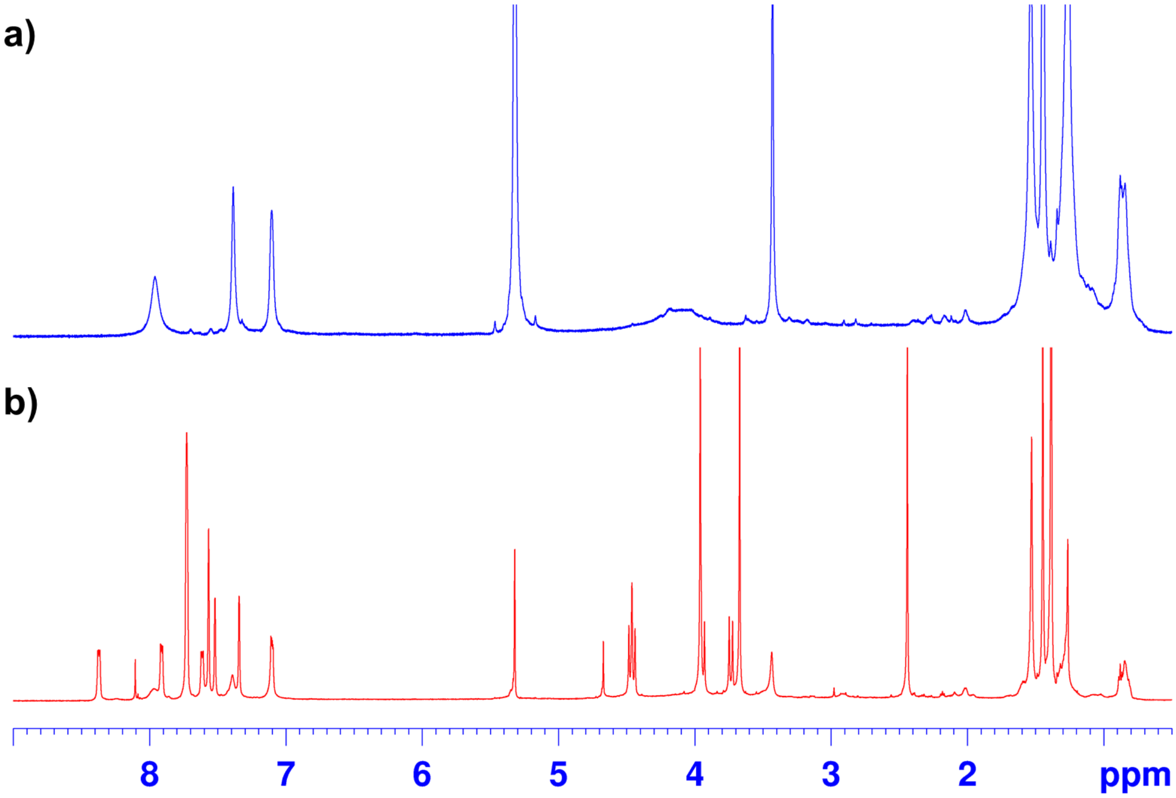

Figure S29: ^{1}H NMR spectra (600 MHz, CD_2Cl_2, 298 K) of: (a) **5;** (b) an equimolar solution (6.2 mM) of **5** and K^+ $[B(Ar^F)_4]^-$ in 0.4 mL of CD_2Cl_2.

Copies of NMR Spectra of $Li^+ \subset 5$

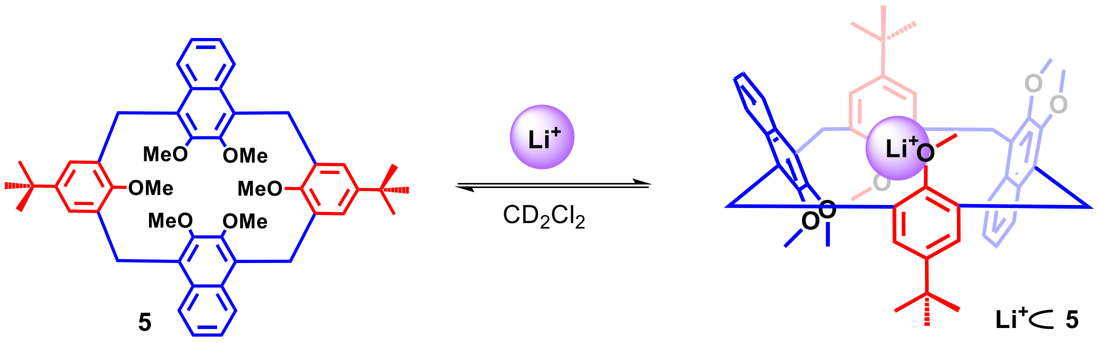

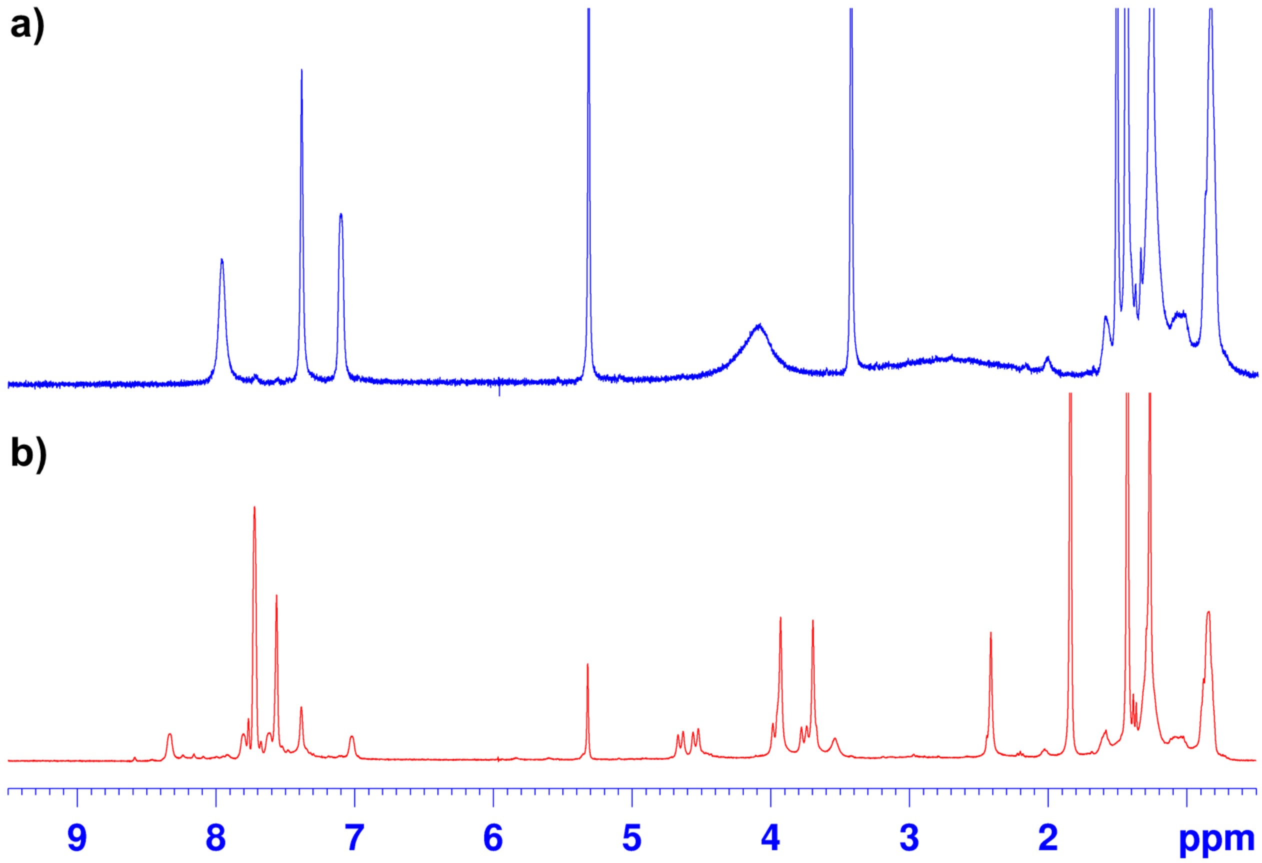

Figure S30: ^1H NMR spectra (400 MHz, CD_2Cl_2, 298 K) of: (a) **5**; (b) an equimolar solution (6.9 mM) of **5** and Li^+ $[B(Ar^F)_4]^-$ in 0.5 mL of CD_2Cl_2.

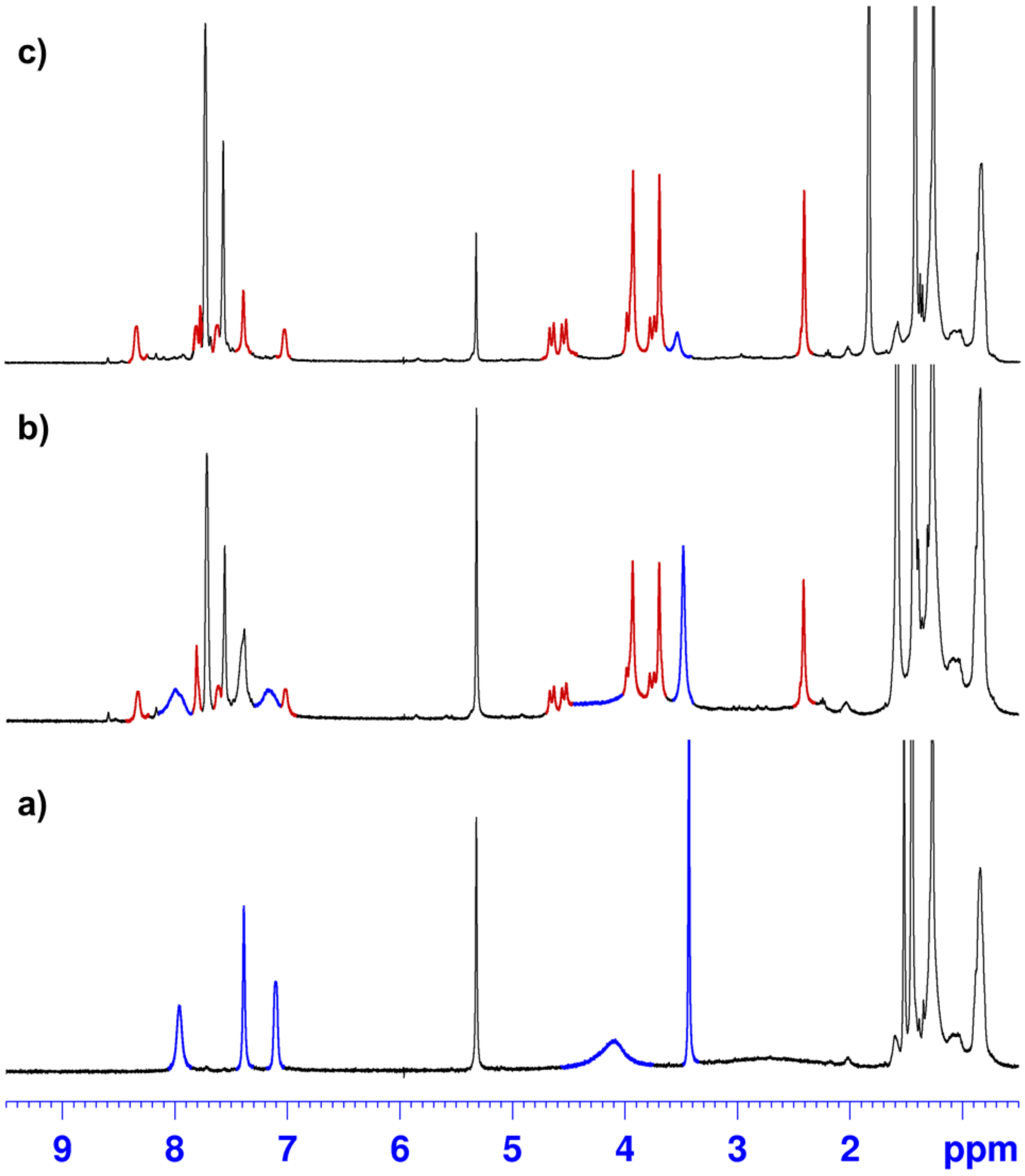

Figure S31: ^{1}H NMR spectrum (400 MHz, CD_2Cl_2, 298 K) of: (a) **5**; (b) **5** and 0.50 equiv of Li[B(Ar^{F})$_4$]; (c) **5** and 1 equiv of Li[B(Ar^{F})$_4$]. In **red** the signals of complex $Li^{+}\subset$**5** and in **blue** the signals of **5**.

Copies of NMR Spectra of $Cs^+ \subset 5$

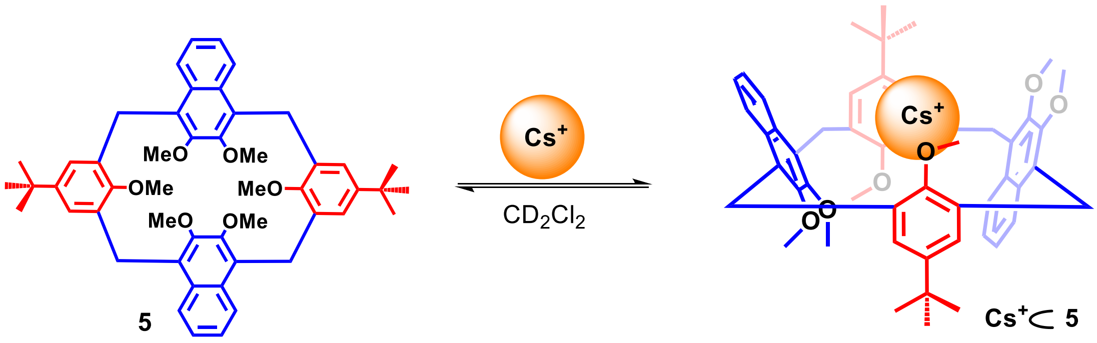

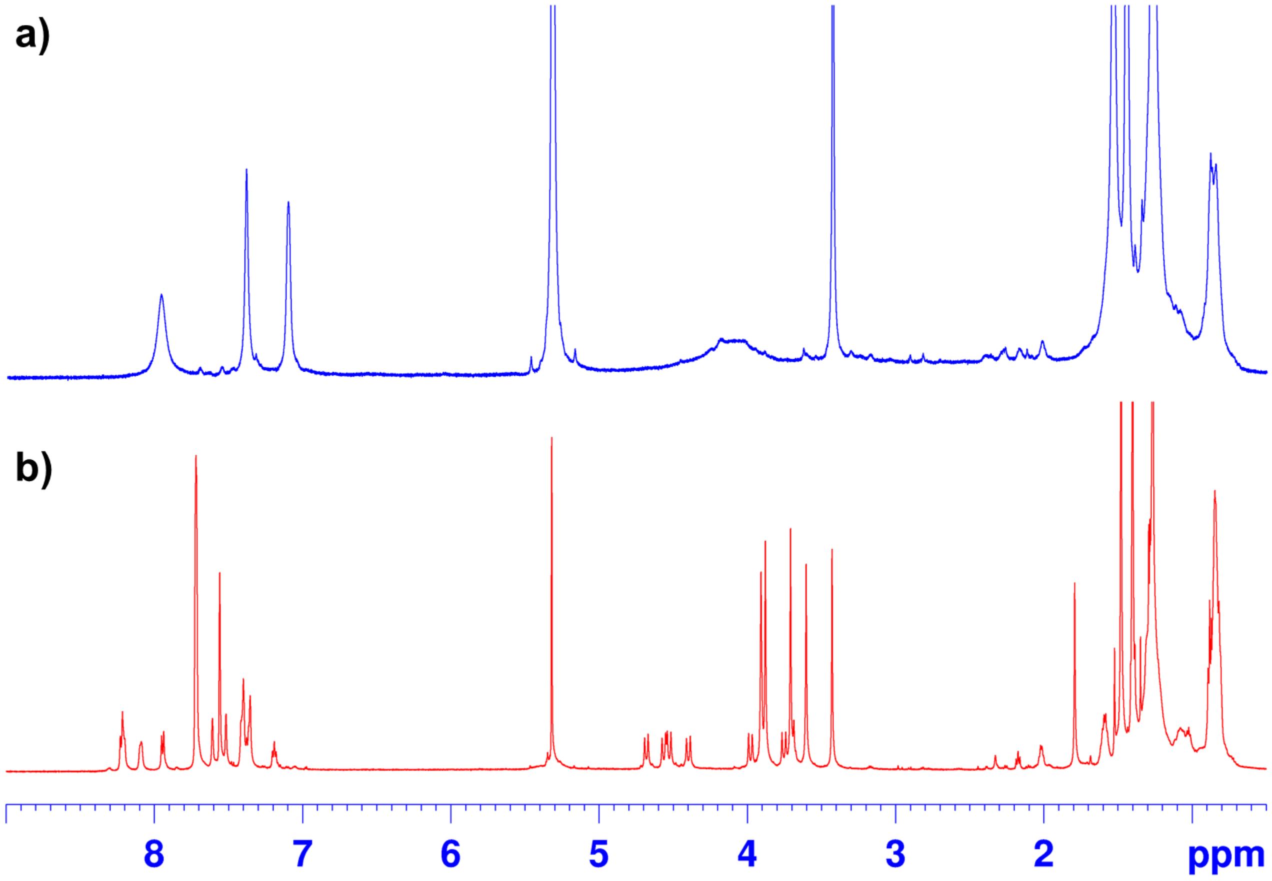

Figure S32: ^{1}H NMR spectra (600 MHz, CD_2Cl_2, 298 K) of: (a) **5**; (b) an equimolar solution (3.9 mM) of **5** and **Cs^+** $[B(Ar^F)_4]^-$ in 0.5 mL of CD_2Cl_2.

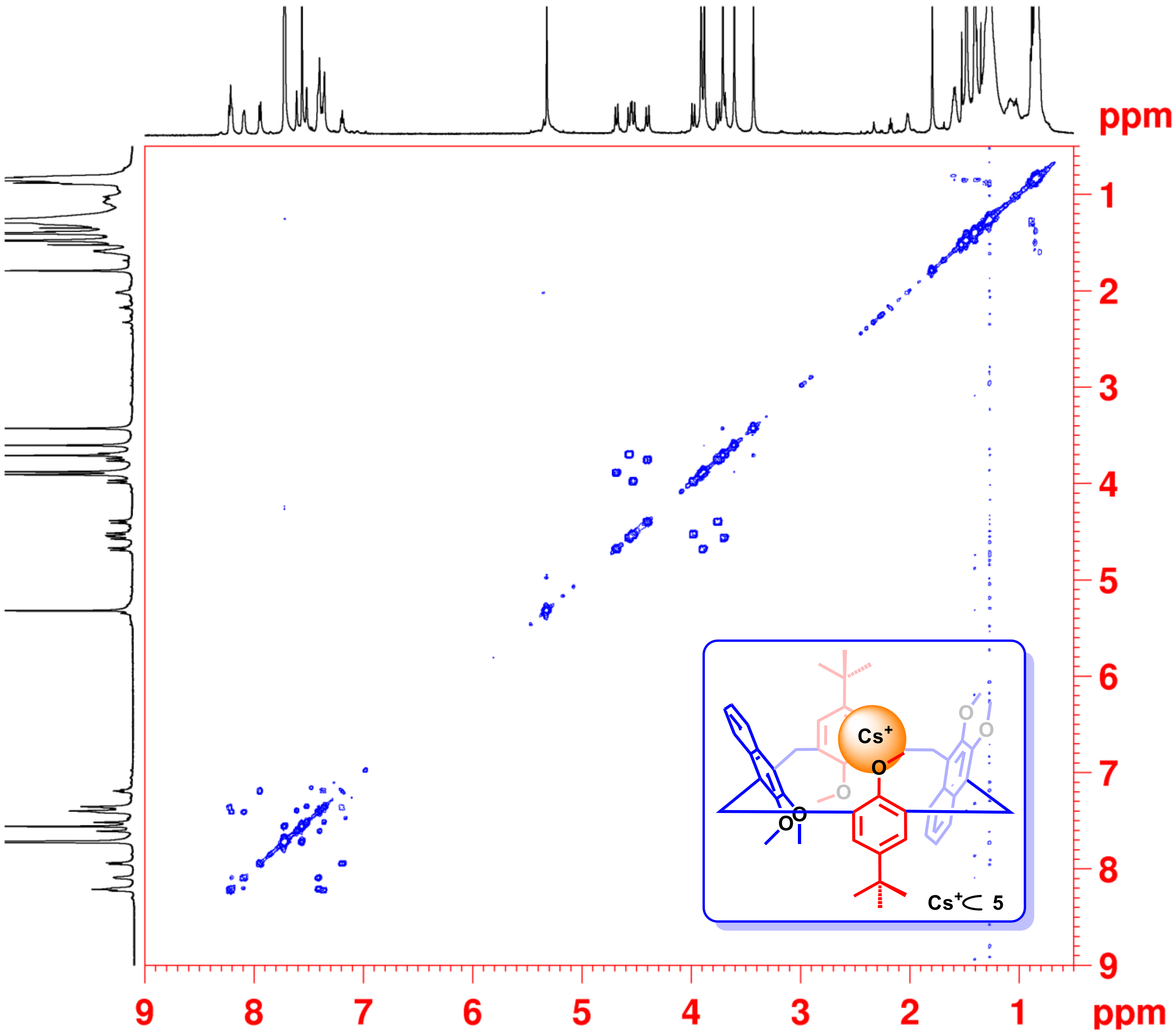

Figure S33: 2D-DQF COSY spectrum of **Cs**$^{+}$$\subset$**5** ($CD_2Cl_2$, 600 MHz, 298 K).

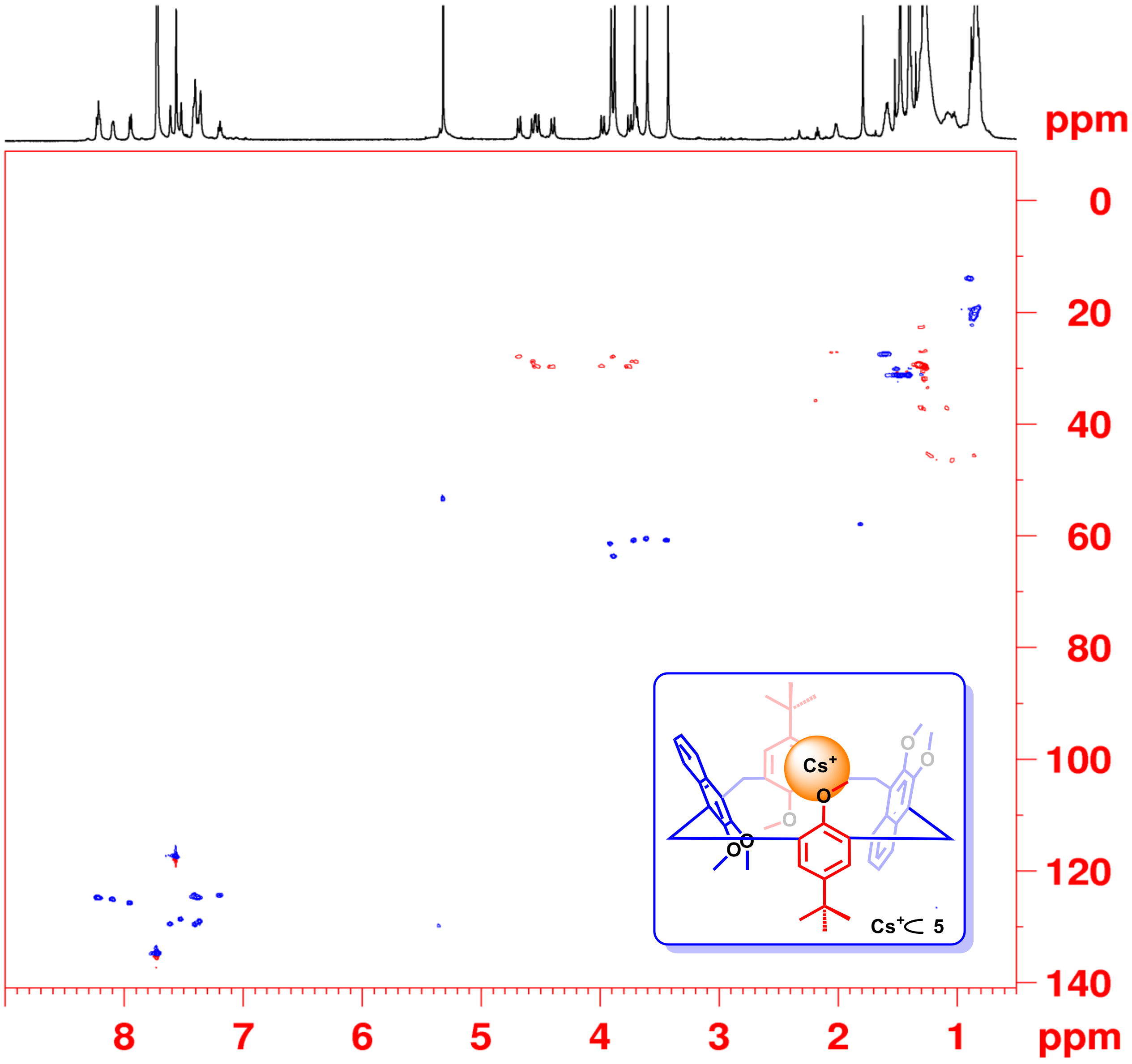

Figure S34: 2D-HSQC spectrum of **$Cs^+ \subset$ 5** (CD_2Cl_2, 600 MHz, 298 K).

Copies of NMR Spectra of $Na^+ \subset 6$

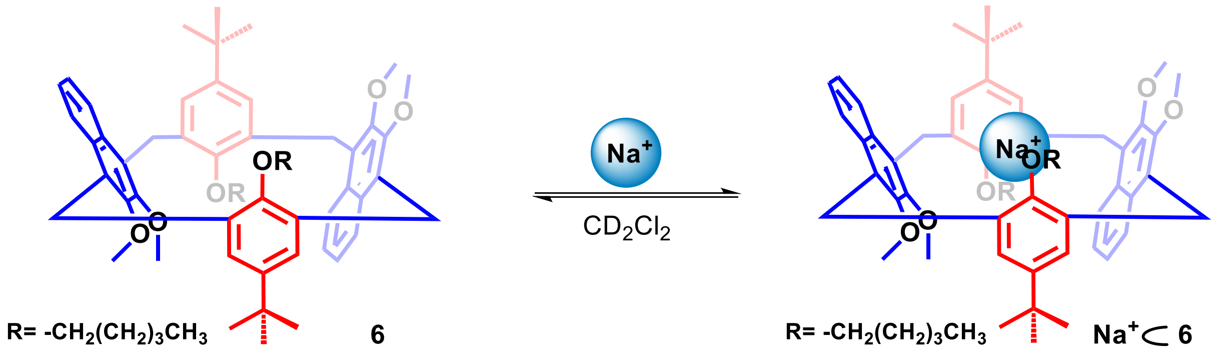

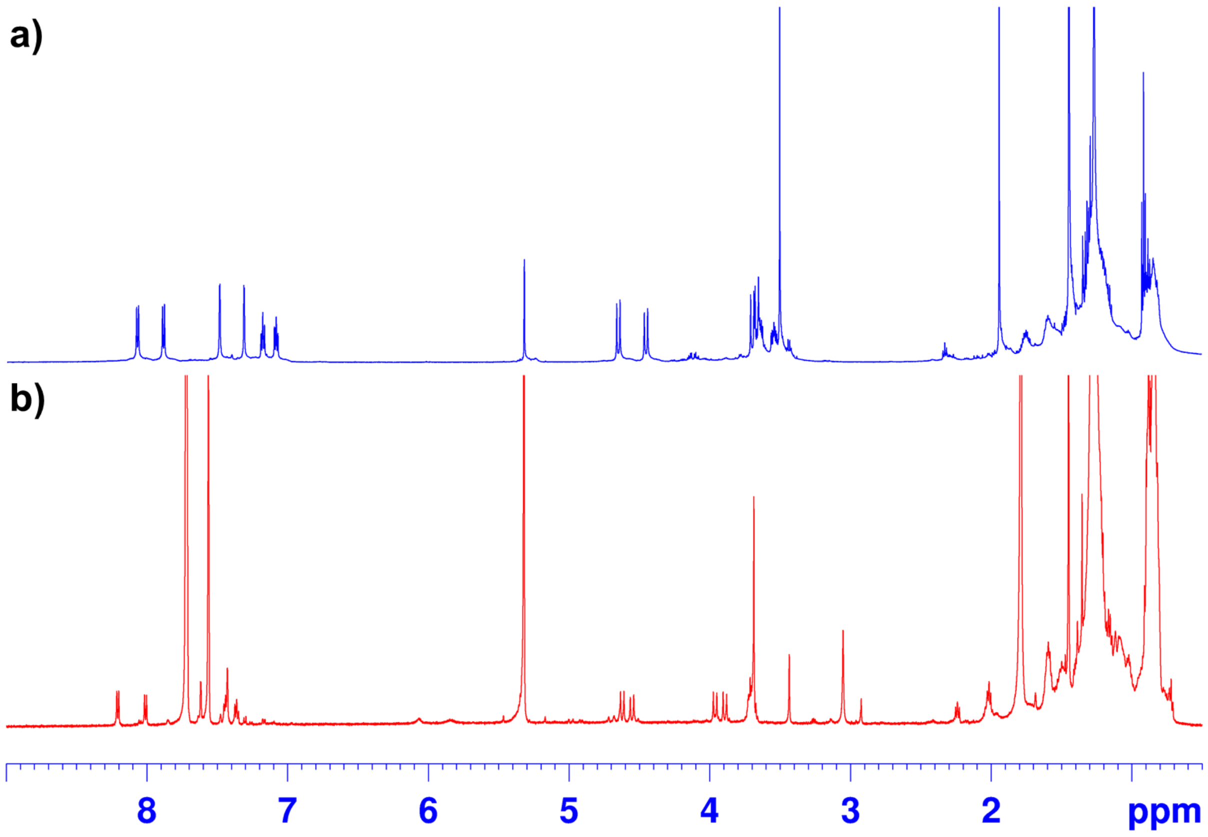

Figure S35: ^{1}H NMR spectra (600 MHz, CD_2Cl_2, 298 K) of: (a) a solution of **6** and (b) an equimolar solution (2.8 mM) of **6** and Na^+ $[B(Ar^F)_4]^-$ in 0.4 mL of CD_2Cl_2.

Copies of NMR Spectra of $K^+ \subset 6$

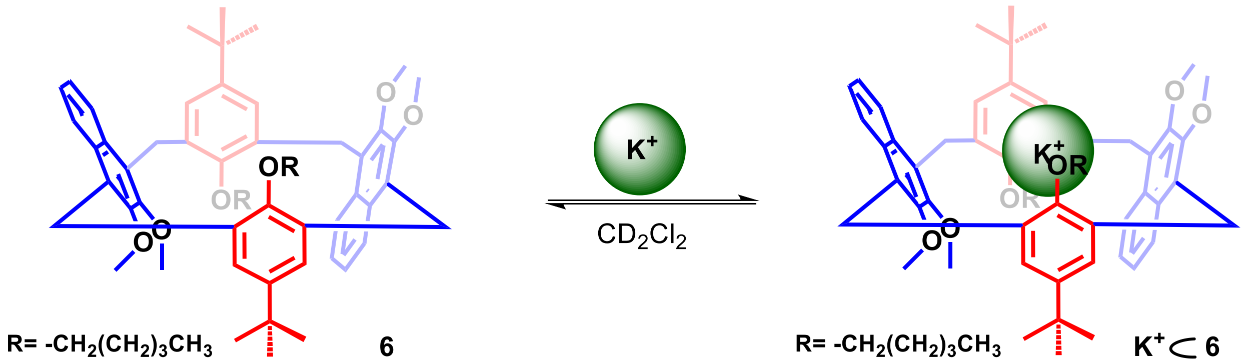

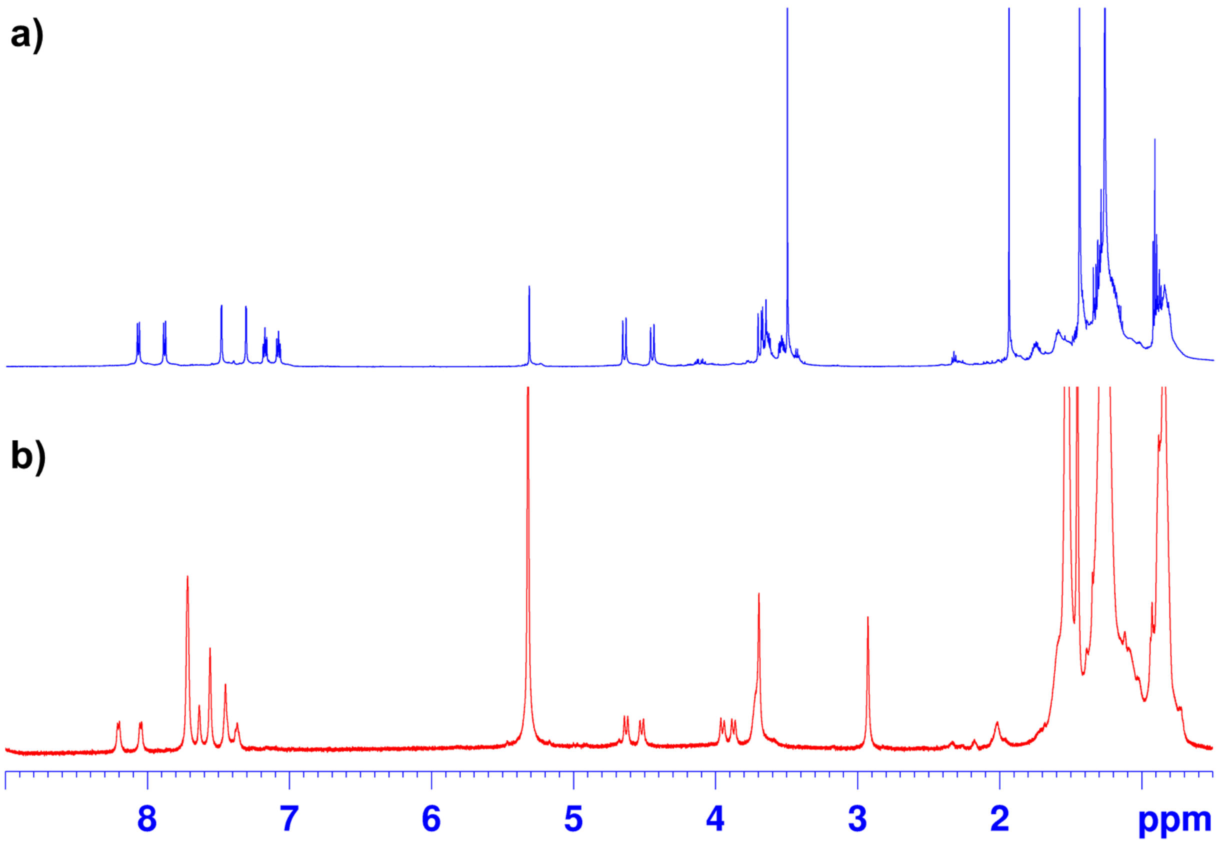

Figure S36: ^{1}H NMR spectra (600 MHz, CD_2Cl_2, 298 K) of: (a) a solution of **6** and (b) an equimolar solution of **6** and K^+ $[B(Ar^F)_4]^-$ (2.8 mM) in 0.4 mL of CD_2Cl_2.

Copies of NMR Spectra of $Li^{+} \subset 6$

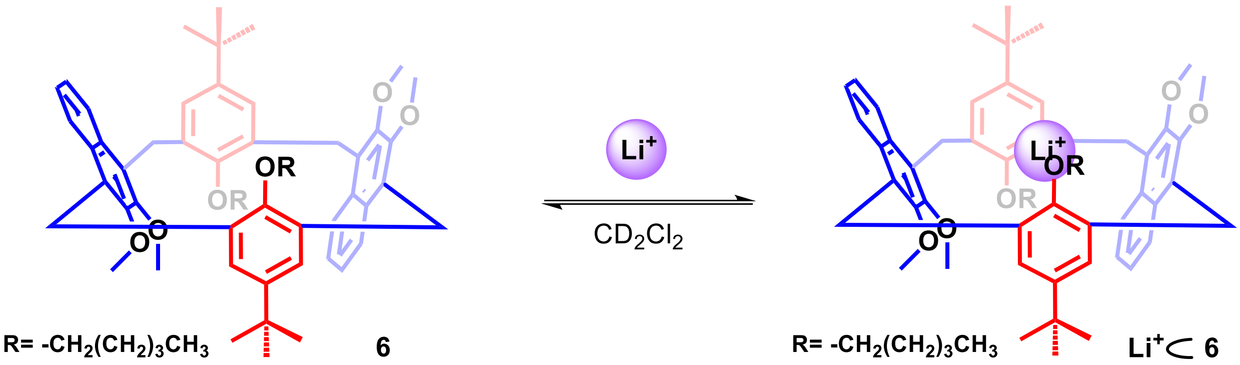

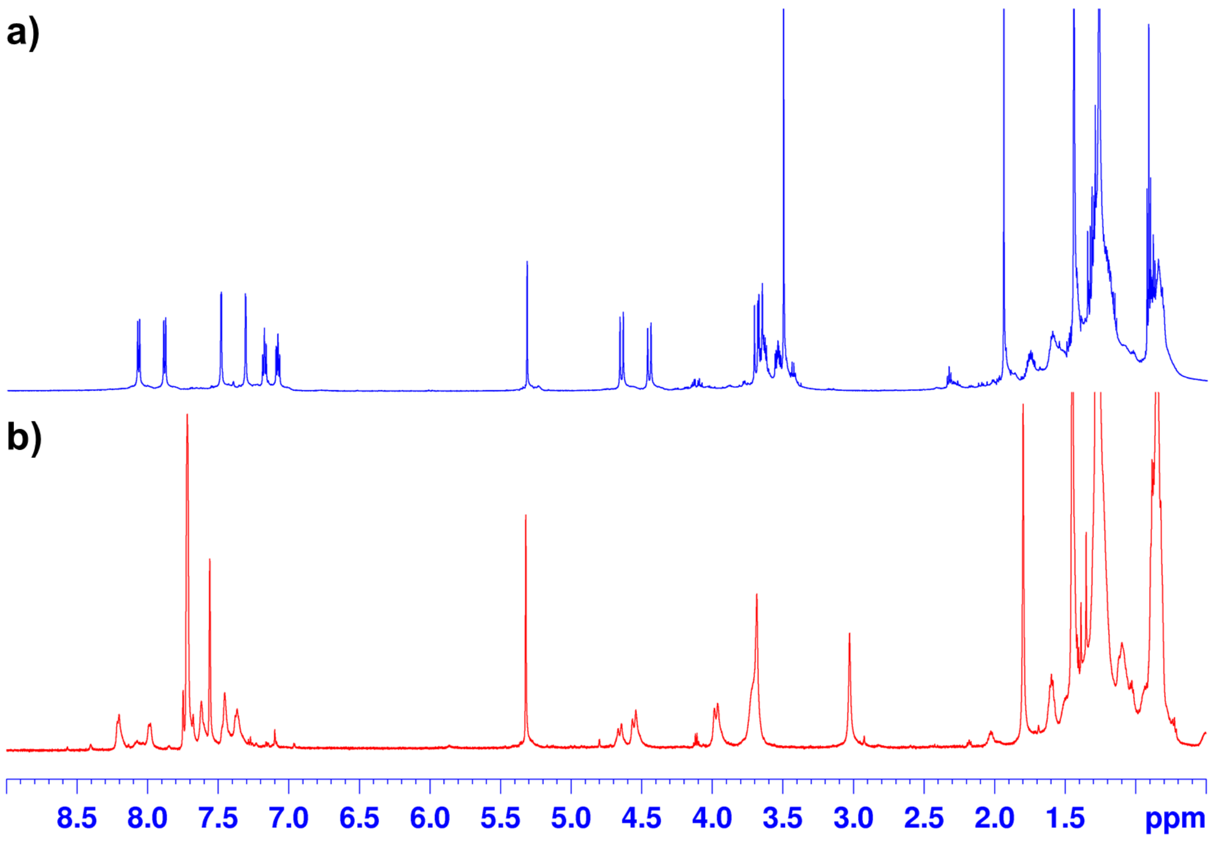

Figure S37: ^{1}H NMR spectra (600 MHz, CD_2Cl_2, 298 K) of: (a) a solution of **6** and (b) an equimolar solution of **6** and Li^{+} $[B(Ar^{F})_4]^{-}$ (6.0 mM) in 0.5 mL of CD_2Cl_2.

Copies of NMR Spectra of Cs$^+$⊂ 6

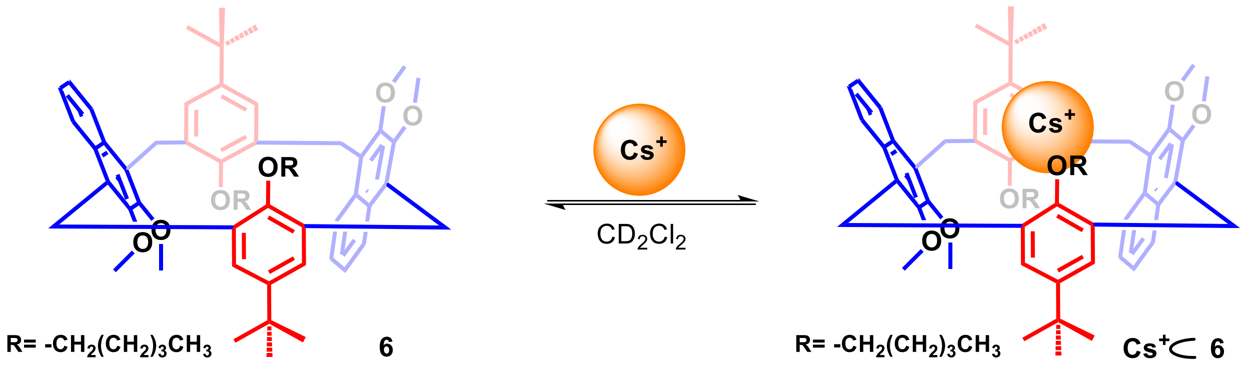

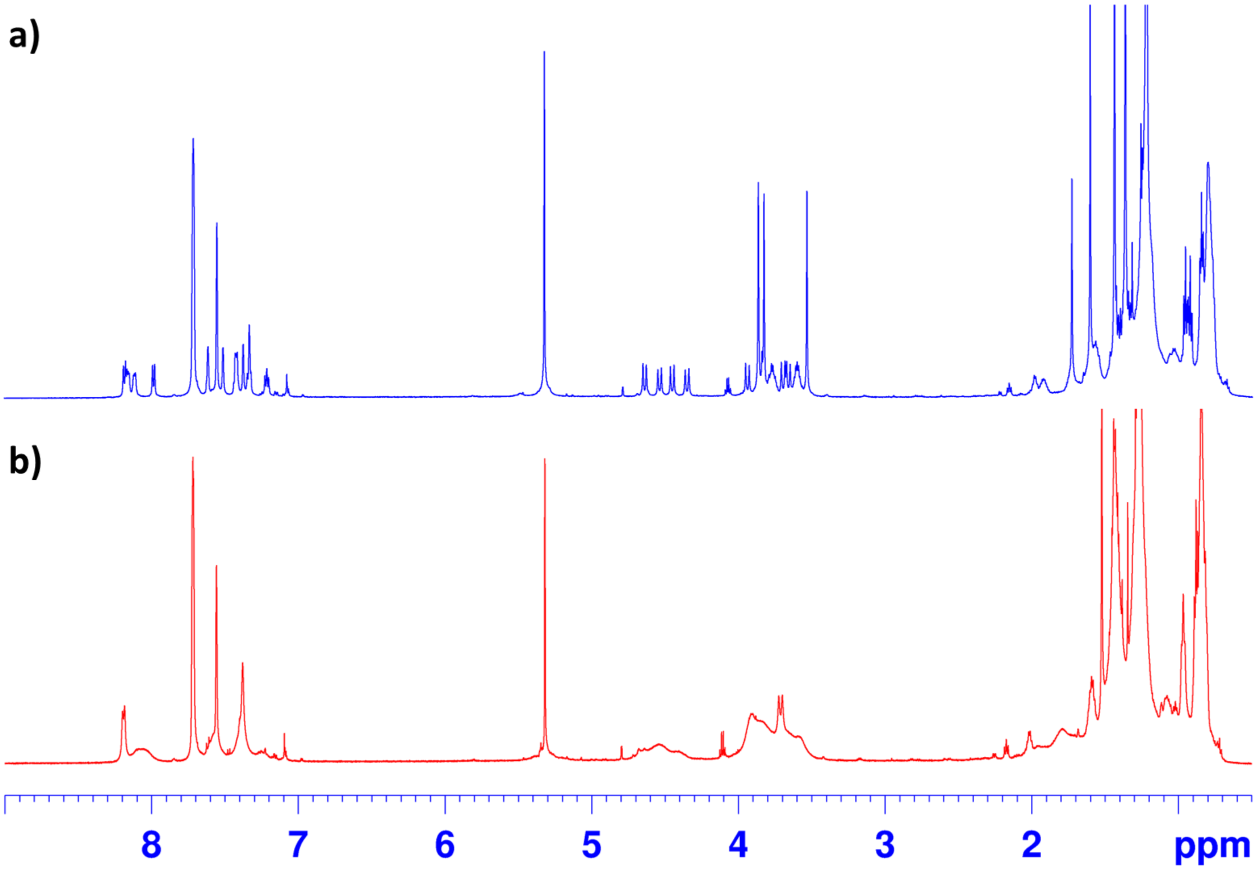

Figure S38: ^{1}H NMR spectra of an equimolar solution (5.3 mM) of **6** and Cs^+ $[B(Ar^F)_4]^-$ (a) at 253 K and (b) at 298 K (600 MHz, CD_2Cl_2).

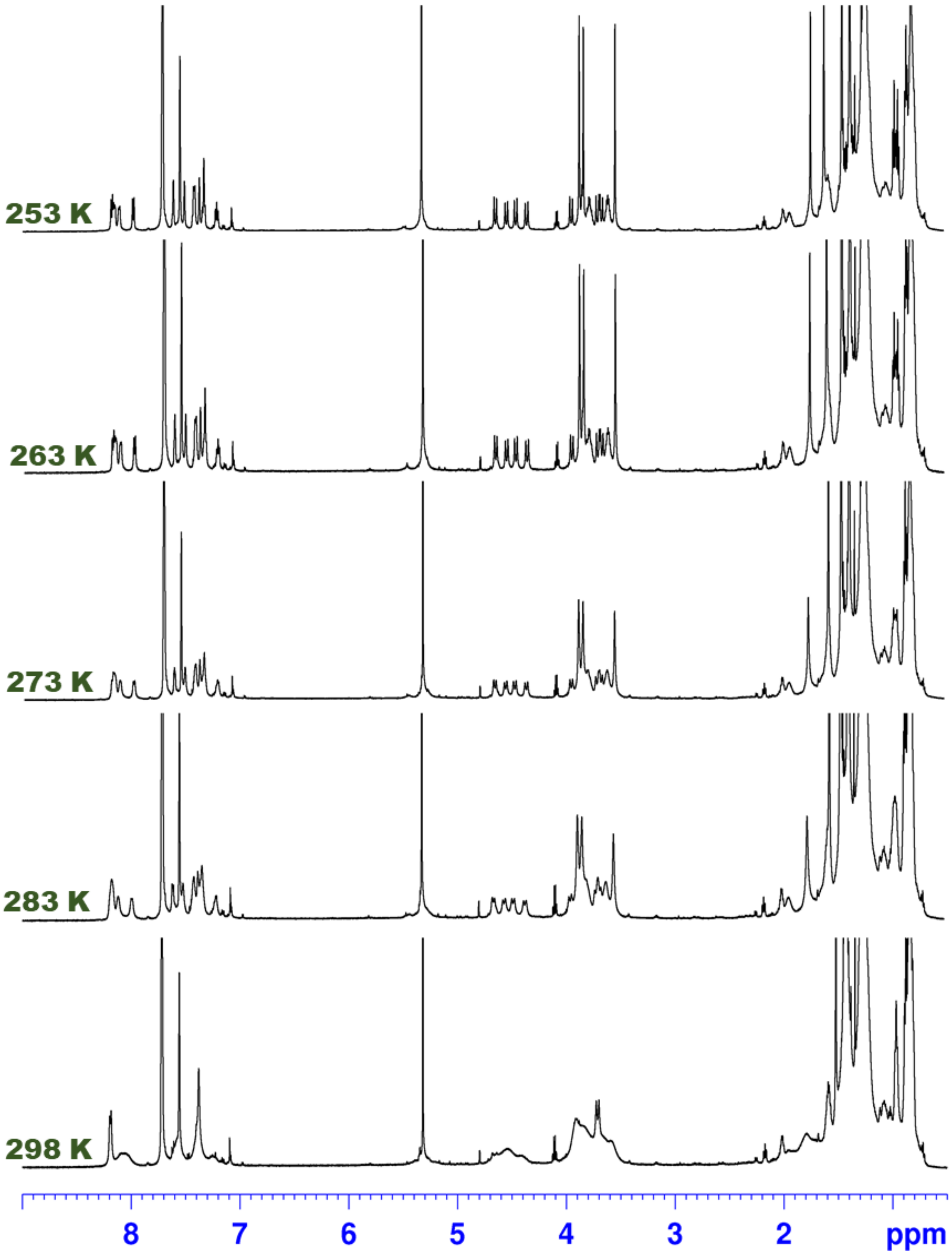

Figure S39: ^{1}H NMR spectra of an equimolar solution (5.3 mM) of **6** and Cs^{+} $[B(Ar^{F})_4]^{-}$ (600 MHz, CD_2Cl_2) at (from bottom to top): 298, 283, 273, 263 and 253 K.

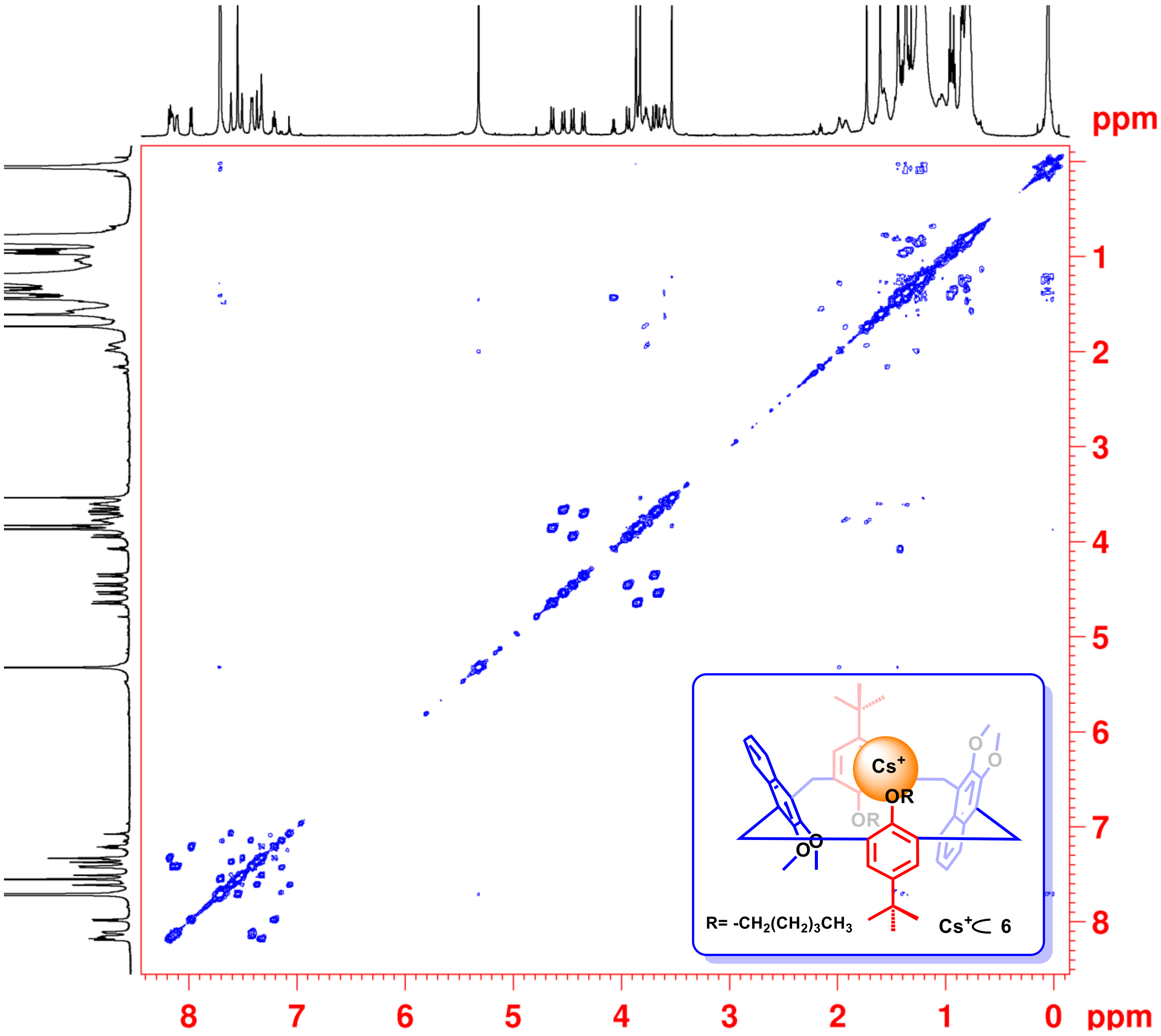

Figure S40: 2D DQF COSY spectrum of $Cs^+ \subset$ **6** (CD_2Cl_2, 600 MHz, 253 K).

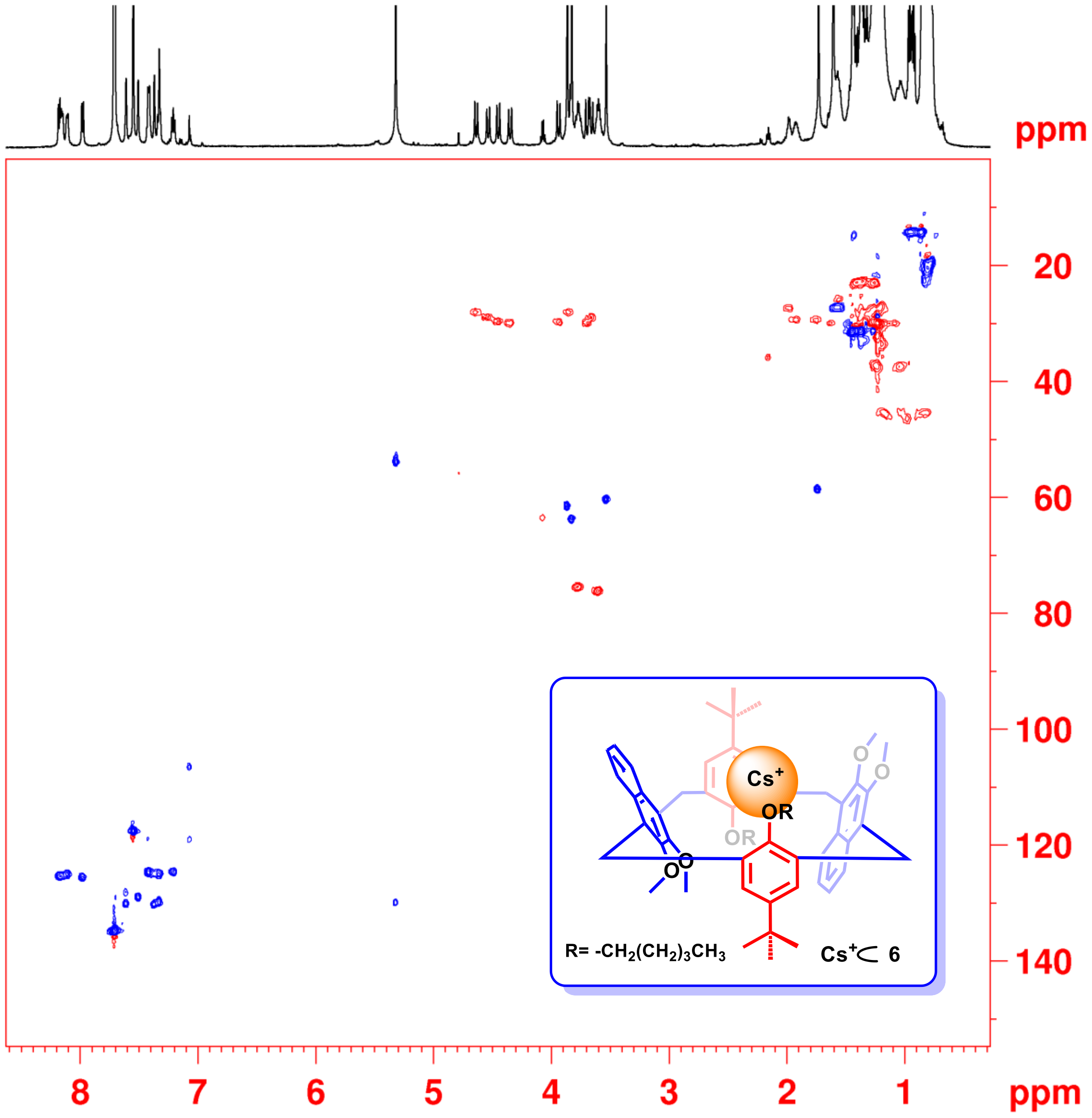

Figure S41: 2D HSQC spectrum of $Cs^+ \subset$ **6** (CD_2Cl_2, 600 MHz, 253 K).

^{1}H NMR determination of K_{ass} values.[4]

The association constant values of the complexes were calculated by means of three methods:

a) ^{1}H NMR competition experiments. In this case, was performed an analysis of a 1:1:1 mixture of host, and two guests in an NMR tube.

b) Integration of free and complexed ^{1}H NMR signals of host. In this case, an equimolar quantity of host and guest was solubilized in CD_2Cl_2.

c) Quantitative ^{1}H NMR experiments using TCE as the internal standard[5]. In this case, ^{1}H NMR experiments were carried out on a 1:1 mixture of host and guest containing a known amount of 1,1,2,2-tetrachloroethane. (d= 1.59 g/mL) as internal standard.

Table S1. Association constant (K_{ass}, M^{-1}) values for the formation of the complexes between the sodium and potassium cations as $[B(Ar^F)_4]^-$ salts and the derivatives **5** and **6**. Determined by ^{1}H NMR experiments in CD_2Cl_2 (400 and 600 MHz).

	**5**	**6**
**Li^+TFPB^-**	$2.0{\pm}0.3{\times}10^3$ [b]	$1.5{\pm}0.3{\times}10^3$ [c]
**Na^+TFPB^-**	$2.2{\pm}0.2{\times}10^3$ [a]	$3.7{\pm}0.3{\times}10^3$ [c]
**K^+TFPB^-**	$2.5{\pm}0.3{\times}10^3$ [b]	$5.1{\pm}0.6{\times}10^3$ [c]
**Cs^+TFPB^-**	$3.0{\pm}0.2{\times}10^3$ [c]	$1.7{\pm}0.2{\times}10^3$ [d]

[a] Calculated by competition experiment at 298 K with K^+ $[B(Ar^F)_4]^-$. [b] Calculated at 298 K by integration of ^{1}H NMR signals of free host and complexed species. [c] Calculated by quantitative ^{1}H NMR spectroscopy at 298 K analysis using TCE as internal standard. [d] Calculated by quantitative ^{1}H NMR study at 253 K analysis using TCE as internal standard.

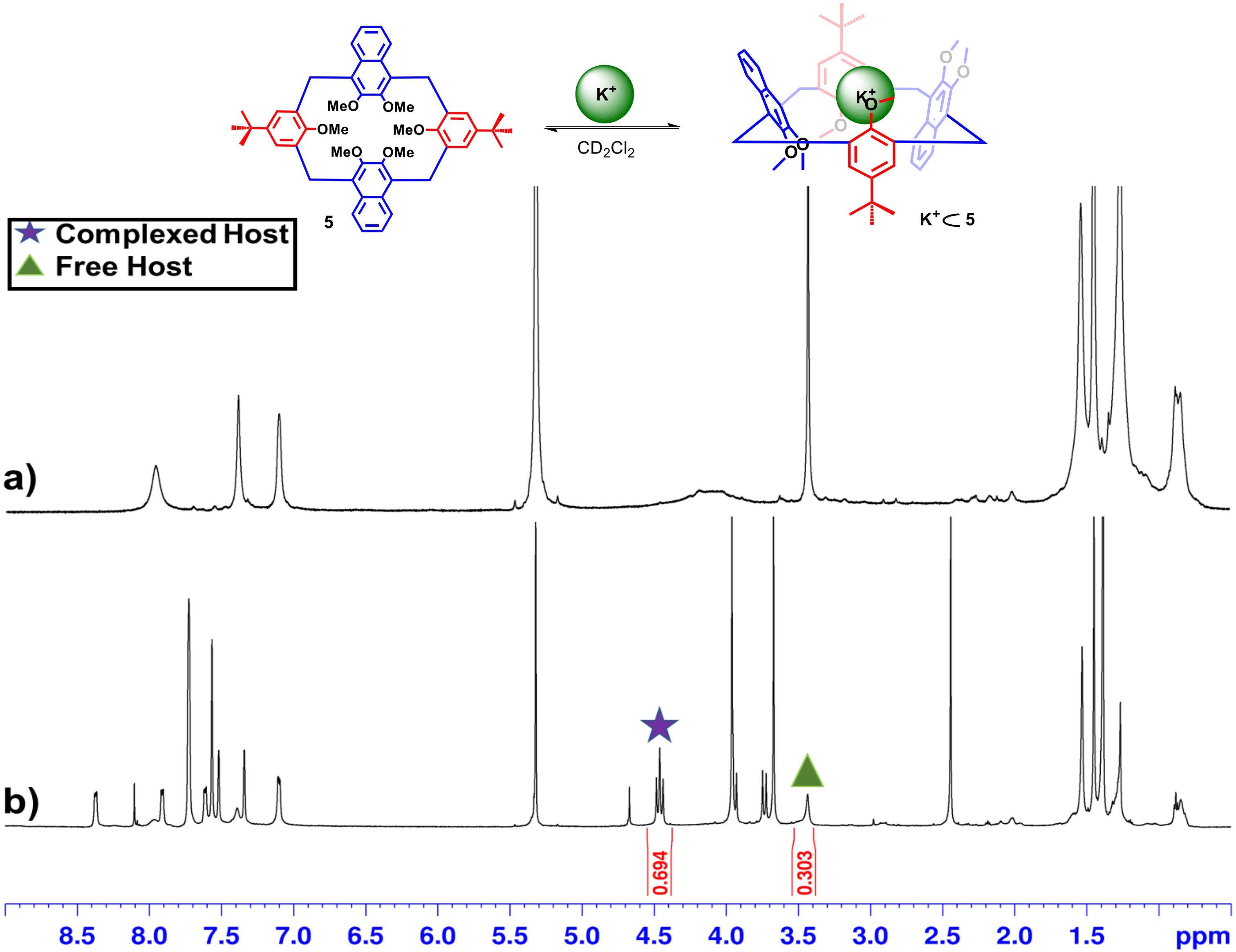

Figure S42: ^{1}H NMR spectra (600 MHz, CD_2Cl_2, 298 K) of: (a) a solution of **5** and (b) an equimolar solution (6.2 mM) of **5** and K^+ $[B(Ar^F)_4]^-$ in 0.4 mL of CD_2Cl_2.

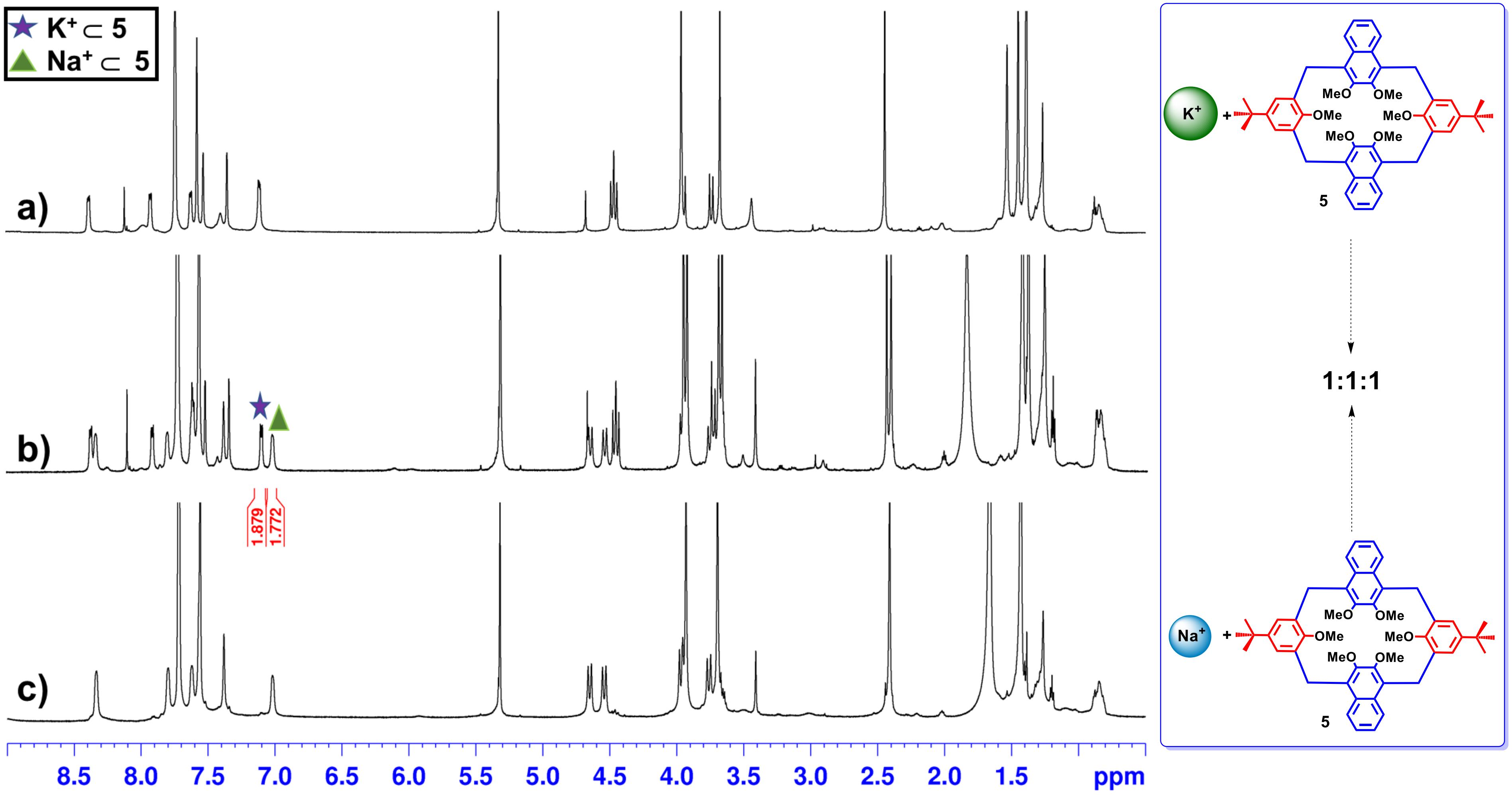

Figure S43: ^{1}H NMR spectra (600 MHz, CD_2Cl_2, 298 K) of: (a) an equimolar solution (6.2 mM) of **5** and K^+ $[B(Ar^F)_4]^-$ in 0.4 mL of CD_2Cl_2 (b) of **5** in the presence of 1 equivalent of Na^+ $[B(Ar^F)_4]^-$ and 1 equivalent of K^+ $[B(Ar^F)_4]^-$ and (c) an equimolar solution (4.3 mM) of **5** and Na^+ $[B(Ar^F)_4]^-$ in 0.4 mL of CD_2Cl_2.

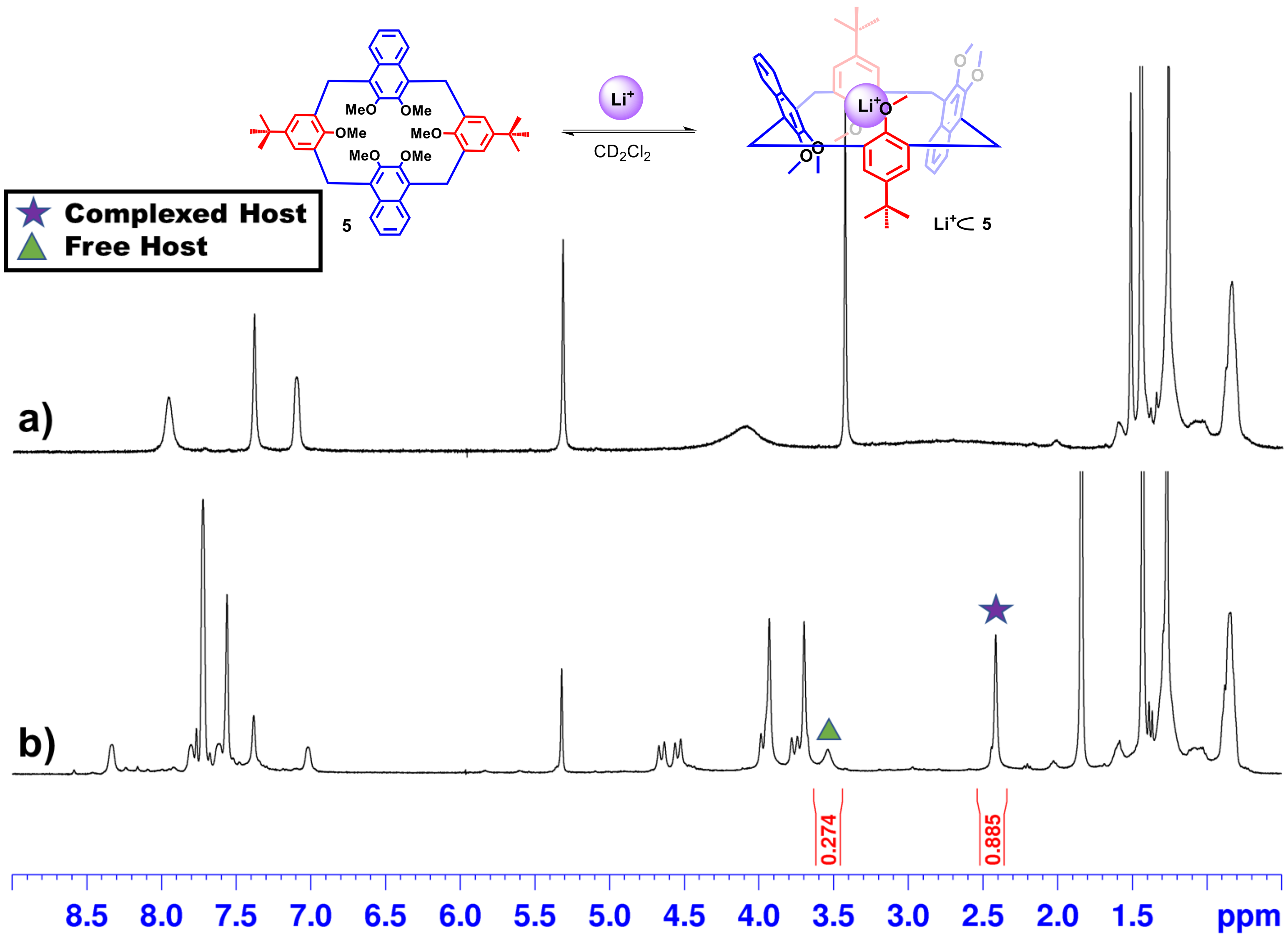

Figure S44: ^{1}H NMR spectra (400 MHz, CD_2Cl_2, 298 K) of: (a) a solution of **5** and (b) an equimolar solution (6.9 mM) of **5** and Li^+ $[B(Ar^F)_4]^-$ in 0.5 mL of CD_2Cl_2.

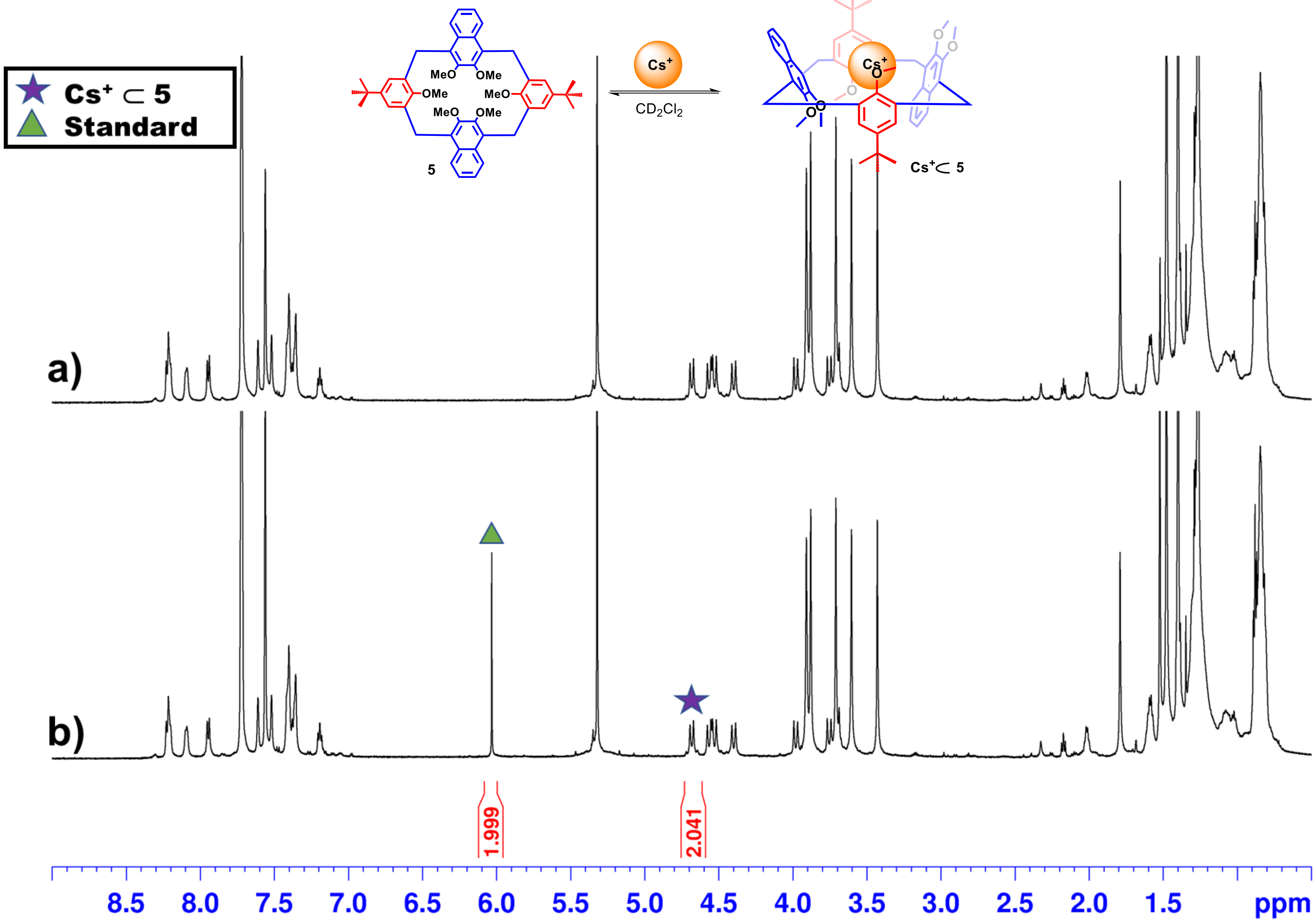

Figure S45: ^{1}H NMR spectra (600 MHz, CD_2Cl_2, 298 K) of: (a) an equimolar solution (3.9 mM) of **5** and Cs^+ $[B(Ar^F)_4]^-$ in 0.5 mL of CD_2Cl_2 (b) of **5** and Cs^+ $[B(Ar^F)_4]^-$ in 0.5 mL of CD_2Cl_2 (after 24 h at 313 K) containing a known amount of 1,1,2,2-tetrachloroethane.

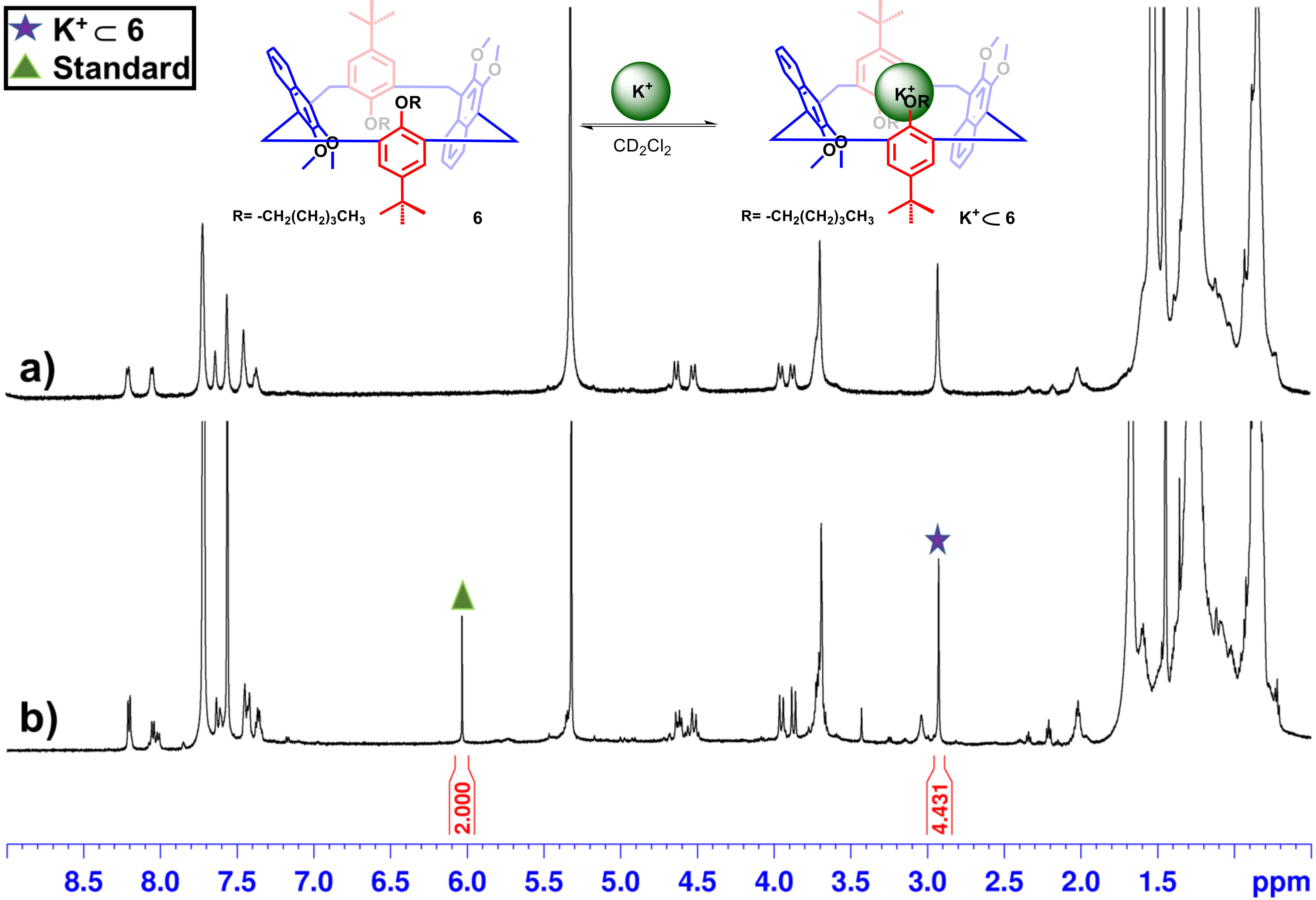

Figure S46: ^{1}H NMR spectrum (600 MHz, CD_2Cl_2, 298 K) of : (a) an equimolar solution (2.8 mM) of **6** and K^+ $[B(Ar^F)_4]^-$ in 0.4 mL of CD_2Cl_2 (b) of **6**, K^+ $[B(Ar^F)_4]^-$ and Na^+ $[B(Ar^F)_4]^-$ in 0.4 mL of CD_2Cl_2 (after 24 h at 313 K) containing 1 equivalent of 1,1,2,2-tetrachloroethane.

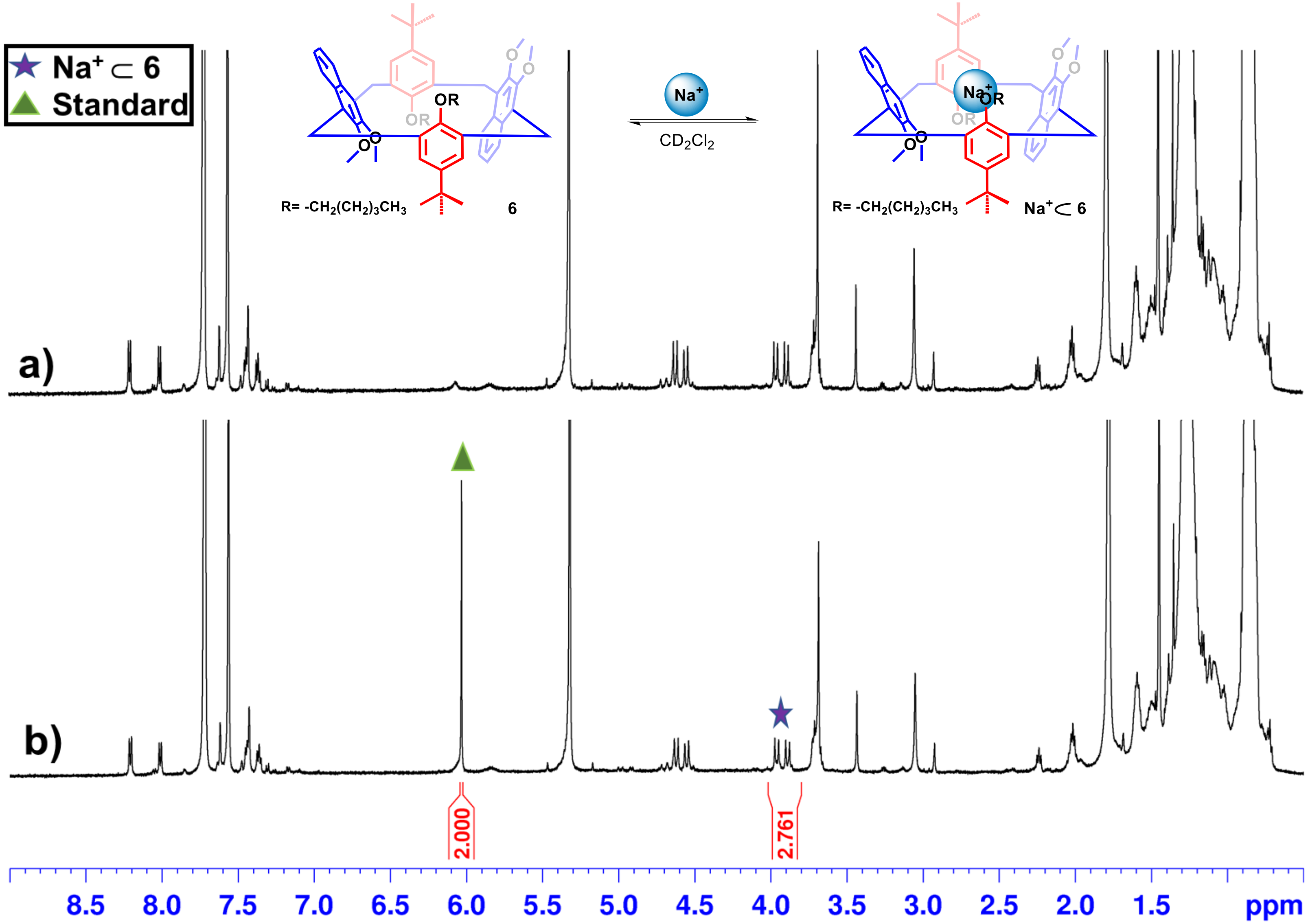

Figure S47: ^{1}H NMR spectrum (600 MHz, CD_2Cl_2, 298 K) of: (a) an equimolar solution (2.8 mM) of **6** and Na^+ $[B(Ar^F)_4]^-$ in 0.4 mL (b) of **6** and Na^+ $[B(Ar^F)_4]^-$ in 0.4 mL of CD_2Cl_2 (after 24 h at 313 K) containing 1 equivalent of 1,1,2,2-tetrachloroethane.

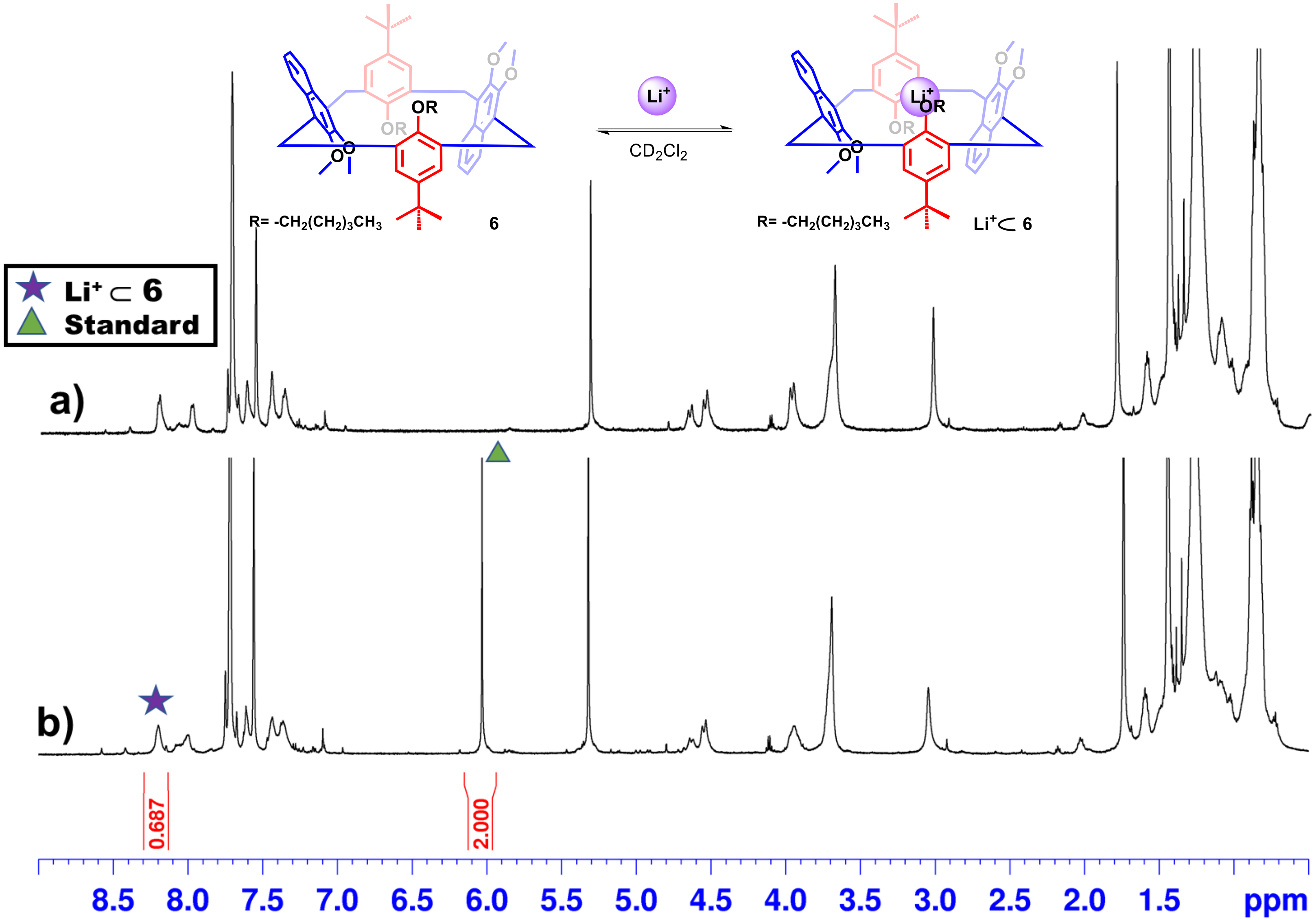

Figure S48: ^{1}H NMR spectrum (600 MHz, CD_2Cl_2, 298 K) of : (a) an equimolar solution (6.0 mM) of **6** and Li^+ $[B(Ar^F)_4]^-$ in 0.5 mL of CD_2Cl_2 (b) of **6** and Li^+ $[B(Ar^F)_4]^-$ in 0.5 mL of CD_2Cl_2 (after 24 h at 313 K) containing a known amount of 1,1,2,2-tetrachloroethane.

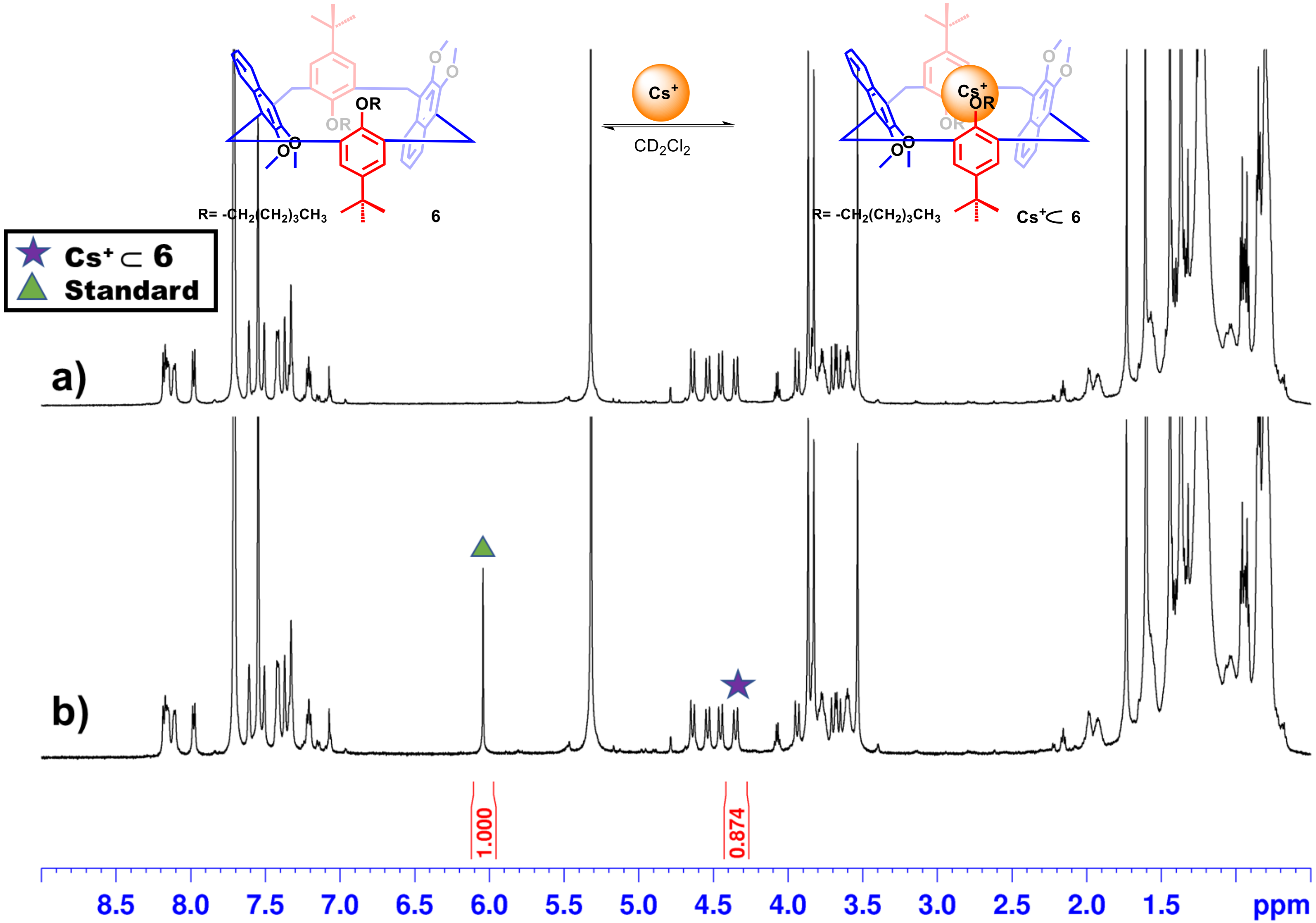

Figure S49: ^{1}H NMR spectrum (600 MHz, CD_2Cl_2, 253 K) of : (a) an equimolar solution (5.3 mM) of **6** and Cs^+ $[B(Ar^F)_4]^-$ in 0.5 mL of CD_2Cl_2 (b) of **6** and Cs^+ $[B(Ar^F)_4]^-$ in 0.5 mL of CD_2Cl_2 (after 24 h at 313 K) containing a known amount of 1,1,2,2-tetrachloroethane.

X-Ray Details of 1

Colorless single crystals suitable for X-ray investigation were obtained by slow evaporation of $CHCl_3$ / Hexane solutions containing **1**. Data collection was carried out at the Macromolecular crystallography XRD1 beamline of the Elettra synchrotron (Trieste, Italy), employing the rotating-crystal method with a Dectris Pilatus 2M area detector. Single crystals investigated were dipped in a PEG 200 cryo-protectant, mounted on a loop and flash-frozen under a liquid nitrogen stream at 100 K. Diffraction data were indexed and integrated using the XDS package,[6] while scaling was carried out with XSCALE.[7] The structures were solved using the SHELXT package;[8] and structure refinement was performed with SHELXL-14,[9] operating through the WinGX GUI,[10] by full-matrix least-squares (FMLS) methods on F^2.

Derivative **1** crystallized in the centrosymmetric triclinic P-1 space group. The asymmetric unit contains a ½ molecule of **1** which lies on a center of inversion and one co-crystallized $CHCl_3$ solvent molecule located outside of the ring. All non-hydrogen atoms of the well-ordered structure were anisotropically refined with hydrogen atoms placed at the geometrically calculated positions using the riding model. Crystal data and final refinement details for the structures are reported in **Table S2.**

X-ray analysis of 1

Small colorless single crystals of **1** suitable for X-ray structure determination were analyzed using synchrotron radiation and cryo-cooling techniques.

The molecule crystallized in the centrosymmetric triclinic P-1 space group. The cyclic molecules lie on crystallographic centers of inversion (C_i molecular point symmetry) and the asymmetric unit contains a ½ molecule of **1**, and one $CHCl_3$ solvent molecules located outside of the macrocycle (**Figure S50a**).

The mean planes of the oppositely oriented naphthalene moieties are almost orthogonal with respect to the mean plane defined by the four bridging methylene groups (dihedral angles of 84°); while the oppositely oriented phenyl ring t-butyl groups of are tilted outwards from the center of the molecule (dihedral angles between phenyl and methylene bridges of 57°). Interestingly, the mean planes of the naphthalene and phenyl moieties are near orthogonal (dihedral angle of 87°) and the aromatic walls define an oblique quadrangular prism (**Figure S50b**). The distances between parallel phenyl rings and parallel naphthalene moieties are 5.3 Å and 5.0 Å respectively. Important intramolecular hydrogen bond interactions are observed between the hydroxy group donors and the adjacent methoxy oxygen acceptors with O•••O distances of 2.782 Å. The prismatic structure is closed above and below by methoxy and methyl groups which protrude towards the center of the macrocycle (**Figure S50c**). The chloroform solvent molecules form interesting symmetric intermolecular C−H···π H-bonds with the arene moieties of **1** (**Figure S50a**). The distance between the H atom of $CHCl_3$ and the barycenter of aromatic ring is 2.3 Å**.**

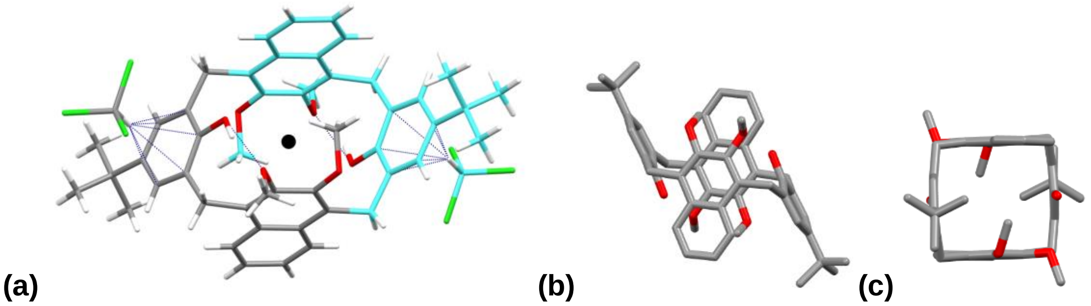

Figure S50: X-ray structure of **1**. (a) Capped stick model of the unit cell which contains one molecule of **1** and two $CHCl_3$ molecules. The center of crystallographic symmetry is indicated as black dot and the symmetry related carbon atoms are shown in grey/cyan colors. H-bonds are shown as dotted lines. (b) View of the **1** molecule evidencing the oblique rectangular prism geometry of the molecule. (c) View of **1** along the direction of the central prism axis.

Table S2. Crystal data and structure refinement for **1.**

Empirical formula	$C_{48}H_{52}O_6$, 2($CHCl_3$)
Formula weight	963.63
Temperature (K)	100(2)
Wavelength (Å)	0.7
Crystal system	Triclinic
Space group	P-1
Unit cell dimensions (Å, °)	a = 9.767(5)<br>b = 10.215(3)<br>c = 13.129(4)<br>α = 77.68(2)<br>β = 76.179(19)<br>γ = 66.86(3)
Volume (Å^3)	1159.1(8)
Z	1
ρ_{calcd} (g/cm^3)	1.38
μ (mm^{-1})	0.415
F(000)	504.0
Reflections collected	18053
Independent reflections	5259
Data / restraints / parameters	5259 / 0 / 286
GooF	1.022
R_1 / wR_2 [I>2σ(I)]	0.069 / 0.189
R_1 / wR_2 all data	0.0886 / 0.2047
Largest. Diff. peak/hole (e Å^{-3})	0.984 / -0.709
CCDC code	1991495

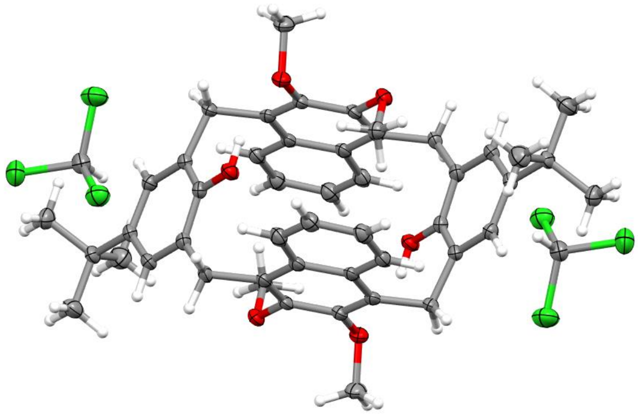

Figure S51. X-ray structure of **1**. The unit cell contains one centrosymmetric molecule of **1** and two $CHCl_3$ molecules. The asymmetric unit is half of the unit cell. Thermal ellipsoids at 50% probability.

Conformational Studies by DFT Calculations

The lowest energy structures for the 5 conformations of **5** in Figures S52 were obtained by molecular mechanics calculations. Successively, the structures were optimized by DFT calculations (Gaussian 16) at B3LYP/6-31G(d,p) level of theory.[11] Finally, single point energies were calculated at B3LYP/6-31G(d,p) level of theory, table S3.

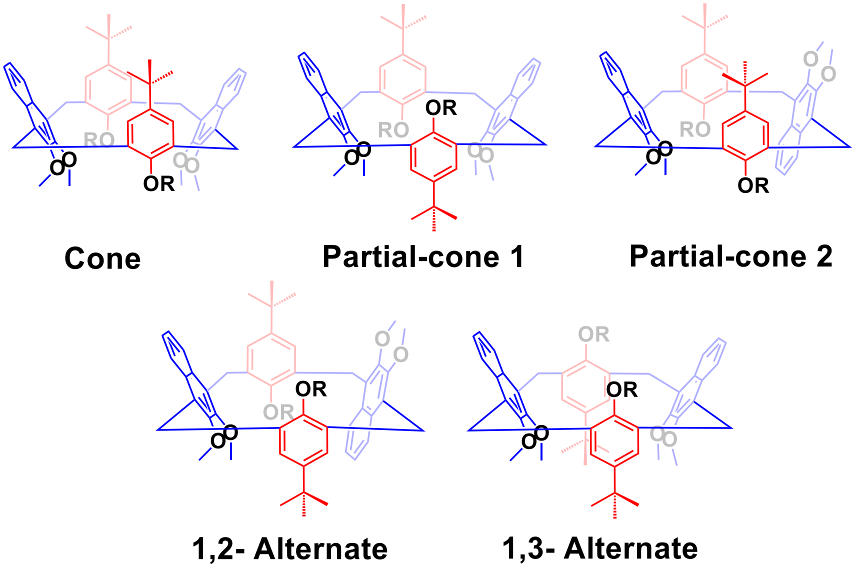

R=H,Me,$CH_2(CH_2)_3CH_3$

Figure S52. Possible conformations of the calix[2]naphtha[2]arene.

Table S3. Single point energies of the five conformations of **5** and Boltzmann populations at 193 K for the conformers of **5.**

	B3LYP/6-31G(d,p) Single point energy (hartree)	ΔE in kcal/mol	Z_i	*Fractional Population*
$5^{1,2\text{-}alt}$	-2390.43096	-	1.0000	99.39
5^{cone}	-2390.42759	2.11	0.0040	0.40
5^{paco2}	-2390.42700	2.48	0.0015	0.15
5^{paco1}	-2390.42632	2.91	0.0005	0.05
$5^{1,3alt}$	-2390.41353	10.94	-	0.01

DFT optimized structures of macrocycle 5

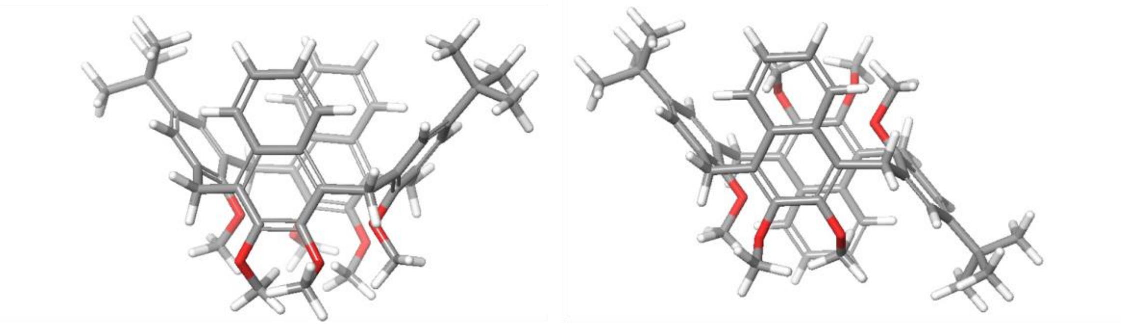

Cone **1,2-Alternate**

Figure S53. DFT-optimized structures of the cone and 1,2-Alternate conformations of **5**.

1) Minimum energy of **cone conformer of 5**

Energy = -2390.42759.
0 imaginary frequency

Atomic coordinates of **cone conformer of 5.**

C	1.363	0.74399	2.67264
C	0.69584	-0.52541	2.74564
C	-0.74031	-0.59232	2.70773
C	-1.51382	0.61387	2.67482
C	-0.84207	1.81588	2.78323
C	0.58581	1.88804	2.74733
C	4.32714	-0.86644	1.35269
C	3.46114	0.23447	1.26774
C	3.14246	0.72276	-0.00726
C	3.53276	0.03177	-1.17006
C	4.38139	-1.06857	-1.02726
C	4.82863	-1.52154	0.22365
C	-1.36864	0.57703	-2.69339
C	-0.70161	-0.69424	-2.68236
C	0.73422	-0.75866	-2.63424
C	1.50853	0.44654	-2.68286
C	0.83711	1.63914	-2.87013
C	-0.5909	1.71374	-2.84066
C	-4.82119	-1.54112	-0.10295
C	-4.32385	-0.95029	-1.27397
C	-3.46597	0.15382	-1.26203
C	-3.14561	0.72198	-0.01643
C	-3.53099	0.10365	1.18372
C	-4.37744	-1.01151	1.11341
C	-1.42411	-1.91809	-2.73757
C	-0.78844	-3.14008	-2.72497
C	0.61853	-3.20199	-2.64604
C	1.3542	-2.03858	-2.6007
C	1.41843	-1.744	2.87255
C	0.78211	-2.96363	2.94498
C	-0.62552	-3.02935	2.88301
C	-1.36119	-1.87087	2.76594
C	2.8803	0.86102	2.5338

O	2.42957	1.90459	-0.12966
O	-2.43533	1.91097	0.02759
O	-1.55663	2.99652	2.87878
O	1.20633	3.11389	2.84005
C	-3.03135	0.60294	2.53788
C	-2.88601	0.70254	-2.5639
C	3.02674	0.44312	-2.55086
O	-1.2129	2.92931	-3.01827
O	1.55205	2.81065	-3.04355
C	5.80649	-2.70909	0.30788
C	7.1053	-2.35889	-0.45682
C	6.1854	-3.05464	1.76038
C	5.15995	-3.96077	-0.33189
C	-5.7891	-2.73644	-0.19397
C	-7.06035	-2.30869	-0.96546
C	-5.10582	-3.90393	-0.94501
C	-6.22045	-3.24741	1.19371
C	-1.56046	3.56374	4.19693
C	0.88147	4.10774	1.85425
C	1.55658	3.28933	-4.3964
C	-0.88717	3.99495	-2.11137
H	4.61162	-1.20635	2.34271
H	4.70336	-1.58231	-1.9295
H	-4.61124	-1.35864	-2.23938
H	-4.69381	-1.46299	2.04766
H	-2.50241	-1.89015	-2.80472
H	-1.37191	-4.05461	-2.77898
H	1.1222	-4.16411	-2.63013
H	2.43339	-2.10022	-2.55154
H	2.49746	-1.71291	2.92812
H	1.36559	-3.87338	3.05312
H	-1.12955	-3.98994	2.93626
H	-2.44082	-1.93491	2.72896
H	3.36836	0.39256	3.39697
H	3.1272	1.9231	2.57744
H	-3.38506	1.61941	2.72155
H	-3.4757	-0.02781	3.31571
H	-3.37391	0.18217	-3.39693
H	-3.13295	1.75996	-2.67184
H	3.4674	-0.23721	-3.28796
H	3.38049	1.44531	-2.80097
H	7.8095	-3.19747	-0.41523
H	6.9102	-2.13968	-1.51073
H	7.59433	-1.48323	-0.01696
H	6.89097	-3.89164	1.76631
H	6.66736	-2.21214	2.26718
H	5.31306	-3.35518	2.34983
H	4.91492	-3.79866	-1.38571
H	5.84641	-4.81328	-0.27748
H	4.23642	-4.23333	0.18924
H	-7.75693	-3.1506	-1.04807
H	-7.57533	-1.49175	-0.44897
H	-6.82622	-1.96962	-1.97896
H	-5.78476	-4.76144	-1.01322
H	-4.82343	-3.62257	-1.96383
H	-4.19955	-4.2276	-0.42282

H	-6.91525	-4.08525	1.0766
H	-5.36787	-3.60584	1.7797
H	-6.73212	-2.47168	1.77279
H	-2.16478	4.47118	4.14092
H	-2.01036	2.8706	4.91746
H	-0.54609	3.81741	4.52109
H	-0.00998	4.67288	2.13304
H	1.74249	4.77803	1.80709
H	0.72942	3.6411	0.87876
H	2.16079	4.19855	-4.40028
H	2.00684	2.55008	-5.06926
H	0.54236	3.52082	-4.73726
H	-1.75646	4.65612	-2.0984
H	-0.7134	3.60285	-1.10728
H	-0.00831	4.55051	-2.44438
C	-3.29614	3.05106	0.13956
H	-3.88378	3.19292	-0.77621
H	-3.98158	2.95301	0.98778
H	-2.65543	3.91698	0.30297
C	3.28898	3.03582	-0.31826
H	3.97585	2.88114	-1.15678
H	2.64742	3.88756	-0.54154
H	3.87491	3.24039	0.58663

2) Minimum energy of **1,2-Alternate conformer of 5**

Energy = -2390.43096.

0 imaginary frequency

Atomic coordinates of **1,2-Alternate conformer of 5.**

C	-1.11262600	-2.71857200	-0.66438100
C	-1.05529900	-2.64707900	0.71597000
C	0.18912300	-2.63974800	1.41622100
C	1.39591700	-2.61661000	0.74377800
C	2.58108500	-2.88360700	-1.42896800
H	3.52867700	-2.82436300	-0.91042400
C	2.57688500	-3.08458900	-2.79122500
H	3.51516300	-3.18122600	-3.32962000
C	1.35024900	-3.17342500	-3.48230100
H	1.34012900	-3.33934200	-4.55564700
C	0.16279900	-3.05508600	-2.79465300
H	-0.76321700	-3.13575400	-3.35003900
C	0.12128800	-2.84914500	-1.38659700
C	1.37492300	-2.76615700	-0.68199100
C	-2.49930600	-1.43963200	2.19359400
H	-1.79043000	-1.33575300	3.01736800
H	-3.51206600	-1.55425000	2.58377600
H	-2.45579200	-0.55375700	1.55404400
C	-0.21554400	-3.84401500	3.41062500
H	-1.22933100	-4.13160300	3.11765100
H	-0.17884400	-3.67697100	4.48878900
H	0.48508800	-4.64389200	3.14282000

C	2.69140500	-2.42392100	1.52360000
H	2.45761600	-2.54176600	2.58334100
H	3.40848400	-3.21228200	1.27031400
C	3.36083400	-1.07378500	1.27638300
C	2.70075800	0.13614800	1.56356800
C	3.24564800	1.36726500	1.17161300
C	4.52677600	1.37880300	0.60277600
H	4.93773900	2.33943200	0.31377200
C	5.26452700	0.20901300	0.39502300
C	4.64100500	-1.00641500	0.71997700
H	5.16314400	-1.94114300	0.53205900
C	6.69075000	0.20956400	-0.18831300
C	7.20054600	1.63217000	-0.48677000
H	6.57921200	2.14083800	-1.23105400
H	8.21862300	1.58123000	-0.88605500
H	7.22918700	2.25203800	0.41525600
C	6.71634300	-0.59728300	-1.50814800
H	6.41301700	-1.63730700	-1.35596400
H	7.72753800	-0.60436200	-1.93050500
H	6.04192300	-0.15617900	-2.24962500
C	7.65847600	-0.44476400	0.82653100
H	7.67339600	0.11350100	1.76857000
H	8.67809600	-0.46064900	0.42520100
H	7.37339300	-1.47646400	1.05299100
C	2.47733900	2.67394600	1.34463900
H	3.09935500	3.47620400	0.93577100
H	2.36997000	2.89010600	2.40887300
O	-2.23502100	-2.63028500	1.42854900
O	0.16943600	-2.60470100	2.79729000
O	1.47359600	0.11376300	2.20116500
C	1.11265000	2.71857800	0.66435100
C	1.05528300	2.64709300	-0.71600000
C	-0.18915900	2.63977500	-1.41621400
C	-1.39593400	2.61662800	-0.74373800
C	-2.58103900	2.88363500	1.42904400
H	-3.52864700	2.82439600	0.91052900
C	-2.57679700	3.08462400	2.79130000
H	-3.51505900	3.18126900	3.32972200
C	-1.35014000	3.17345800	3.48233900
H	-1.33998800	3.33938000	4.55568300
C	-0.16271200	3.05511100	2.79465500
H	0.76332100	3.13577700	3.35001200
C	-0.12124400	2.84915800	1.38660100
C	-1.37490000	2.76617300	0.68203100
C	2.49920400	1.43968300	-2.19372900
H	1.79014500	1.33573200	-3.01733600
H	3.51185800	1.55440600	-2.58415500
H	2.45593500	0.55380800	-1.55416300
C	0.21547100	3.84403900	-3.41063300
H	1.22926300	4.13161900	-3.11767300
H	0.17875800	3.67698500	-4.48879600
H	-0.48515200	4.64392400	-3.14282800
C	-2.69144200	2.42394600	-1.52352900
H	-2.45768500	2.54183100	-2.58327300
H	-3.40852400	3.21229000	-1.27019700
C	-3.36084800	1.07379200	-1.27634500

C	-2.70075400	-0.13612500	-1.56355800
C	-3.24562300	-1.36725800	-1.17162700
C	-4.52674900	-1.37882800	-0.60278600
H	-4.93769700	-2.33947100	-0.31380300
C	-5.26452100	-0.20905500	-0.39501400
C	-4.64101900	1.00638900	-0.71994300
H	-5.16317300	1.94110600	-0.53200600
C	-6.69074800	-0.20964200	0.18831300
C	-7.20051800	-1.63226200	0.48674800
H	-6.57917400	-2.14093000	1.23102200
H	-8.21859600	-1.58134800	0.88603500
H	-7.22914900	-2.25211700	-0.41528800
C	-6.71637100	0.59718700	1.50815800
H	-6.41306600	1.63722000	1.35599200
H	-7.72757100	0.60423900	1.93050500
H	-6.04195100	0.15608700	2.24963700
C	-7.65847800	0.44468000	-0.82653200
H	-7.67337700	-0.11357400	-1.76857900
H	-8.67810200	0.46054000	-0.42521300
H	-7.37341000	1.47638700	-1.05297700
C	-2.47730000	-2.67392400	-1.34469700
H	-3.09931700	-3.47620400	-0.93587600
H	-2.36990900	-2.89003500	-2.40893900
O	2.23497800	2.63031400	-1.42862800
O	-0.16951600	2.60473300	-2.79728400
O	-1.47359600	-0.11371900	-2.20116500
C	1.56972500	0.11527600	3.62831900
H	2.11453900	0.99652800	3.98952900
H	0.54818500	0.13979100	4.00831600
H	2.06933200	-0.78640900	3.99870900
C	-1.56973500	-0.11527300	-3.62832000
H	-2.11474200	-0.99642400	-3.98948500
H	-2.06914200	0.78650700	-3.99874600
H	-0.54820300	-0.14003700	-4.00832400

3) Minimum energy of **partial-cone 1 conformer of 5**

Energy = -2390.42632.

0 imaginary frequency

Atomic coordinates of **partial-cone 1 conformer of 5.**

C	1.251600	-2.663000	0.818300
C	-0.023300	-2.711400	1.472600
C	-1.227800	-2.750900	0.684400
C	-1.165100	-2.657700	-0.746300
C	0.082700	-2.721800	-1.345300
C	1.274900	-2.768500	-0.559900
C	4.510100	-1.149000	0.638500
C	3.273400	-1.183000	1.297100
C	2.690000	0.034200	1.677000
C	3.253300	1.263100	1.290000
C	4.490000	1.243100	0.640400
C	5.162200	0.050100	0.330700
C	-1.345400	2.714600	-0.533200

C	-1.319900	2.747200	0.900100
C	-0.064200	2.778200	1.604800
C	1.167800	2.702100	0.871200
C	1.105600	2.750200	-0.510200
C	-0.141100	2.802600	-1.204700
C	-5.238600	-0.122600	-0.488900
C	-4.602800	1.111600	-0.662800
C	-3.313900	1.223700	-1.202300
C	-2.660500	0.049400	-1.607900
C	-3.208800	-1.217900	-1.343700
C	-4.501400	-1.271300	-0.813300
C	-2.523600	2.804500	1.657100
C	-2.515100	2.909800	3.029600
C	-1.286300	2.956000	3.721100
C	-0.101100	2.885300	3.023700
C	-0.145900	-2.786400	2.888700
C	-1.367300	-2.920900	3.509200
C	-2.545300	-2.983800	2.735600
C	-2.469900	-2.894900	1.364000
C	2.578200	-2.517600	1.558000
O	1.514100	0.026500	2.408800
O	-1.455000	0.141000	-2.285100
O	0.164300	-2.796800	-2.716800
O	2.495700	-2.869500	-1.201900
C	-2.417600	-2.497700	-1.606400
C	-2.643900	2.591400	-1.321800
C	2.535200	2.586700	1.540400
O	-0.124900	2.893200	-2.584200
O	2.282300	2.801700	-1.227000
C	6.550800	0.098900	-0.336500
C	6.462300	0.843400	-1.689300
C	7.534200	0.850600	0.592400
C	7.122700	-1.306700	-0.601700
C	-6.673400	-0.256800	0.056800
C	-6.658000	-1.079700	1.366900
C	-7.315400	1.110600	0.358400
C	-7.553800	-0.980800	-0.989500
C	0.980000	-1.829400	-3.398800
C	2.768000	-4.180000	-1.719700
C	2.544000	1.690400	-2.102000
C	0.243100	4.188600	-3.080900
H	4.955600	-2.097200	0.362000
H	4.927700	2.196500	0.358500
H	-5.114000	2.026800	-0.384100
H	-4.945100	-2.250600	-0.654700
H	-3.473000	2.773500	1.139000
H	-3.451600	2.962700	3.577000
H	-1.273200	3.048600	4.803200
H	0.827000	2.928800	3.579600
H	0.745600	-2.759400	3.502200
H	-1.421200	-2.989000	4.591900
H	-3.510000	-3.106300	3.219400
H	-3.381100	-2.953000	0.785600
H	3.256400	-3.313500	1.237100
H	2.430800	-2.658600	2.629600
H	-3.089100	-3.350400	-1.451000

H	-2.101000	-2.538400	-2.649900
H	-2.414500	2.809700	-2.366500
H	-3.360600	3.351600	-0.992700
H	3.179400	3.386700	1.163700
H	2.440200	2.755300	2.614200
H	7.449400	0.890400	-2.163200
H	5.781800	0.327900	-2.375000
H	6.104200	1.869900	-1.567000
H	8.528500	0.902000	0.134400
H	7.203600	1.874900	0.788600
H	7.630600	0.339000	1.555900
H	8.112000	-1.220000	-1.062400
H	7.237400	-1.880800	0.323500
H	6.489300	-1.882100	-1.284700
H	-7.674000	-1.179000	1.765400
H	-6.263400	-2.088000	1.209900
H	-6.039800	-0.592200	2.127800
H	-8.334300	0.963300	0.730800
H	-6.760800	1.661600	1.124900
H	-7.377900	1.738000	-0.536700
H	-8.577200	-1.092900	-0.614000
H	-7.596200	-0.413500	-1.925300
H	-7.172600	-1.980000	-1.220400
H	0.573400	-1.753700	-4.409500
H	0.914600	-0.857600	-2.905300
H	2.023800	-2.145400	-3.446000
H	2.036200	-4.465000	-2.482400
H	3.763400	-4.134900	-2.165400
H	2.762300	-4.925600	-0.915900
H	3.527900	1.875900	-2.535600
H	2.572000	0.755100	-1.535600
H	1.794200	1.629200	-2.893400
H	-0.467300	4.951200	-2.740200
H	1.253600	4.461200	-2.763000
H	0.207000	4.121400	-4.169900
C	-1.643200	0.313100	-3.695200
H	-0.662000	0.511100	-4.124600
H	-2.071000	-0.589800	-4.148600
H	-2.301300	1.161700	-3.909800
C	1.725000	0.031500	3.822400
H	2.275300	0.923900	4.145100
H	0.738200	0.028500	4.286800
H	2.284300	-0.854000	4.149700

4) Minimum energy of **partial-cone 2 conformer of 5**
Energy = -2390.42700.
0 imaginary frequency

Atomic coordinates of **partial-cone 2 conformer of 5.**

C	-1.362400	0.342700	2.748300
C	-0.596900	-0.864400	2.645000
C	0.840300	-0.807600	2.655900
C	1.515600	0.459800	2.675400

C	0.748100	1.598300	2.862000
C	-0.678400	1.526300	2.943600
C	-4.285100	-1.120100	1.147000
C	-3.436700	-0.012700	1.289500
C	-3.098400	0.715700	0.138500
C	-3.488400	0.274100	-1.138100
C	-4.318300	-0.845300	-1.226600
C	-4.762200	-1.547800	-0.096900
C	1.379900	0.938300	-2.646200
C	0.698900	2.201100	-2.681200
C	-0.740900	2.246800	-2.600900
C	-1.495300	1.027900	-2.559900
C	-0.808500	-0.165300	-2.684600
C	0.616700	-0.215600	-2.672100
C	4.718200	-1.593200	-0.210300
C	4.221300	-0.973900	-1.361900
C	3.443300	0.190300	-1.309200
C	3.173700	0.752700	-0.053600
C	3.531500	0.091200	1.134400
C	4.330600	-1.051000	1.025400
C	1.400800	3.436000	-2.785200
C	0.751100	4.649200	-2.807000
C	-0.656100	4.694000	-2.714200
C	-1.372500	3.523400	-2.610700
C	-1.225300	-2.139400	2.591600
C	-0.496100	-3.307900	2.580100
C	0.912800	-3.255600	2.623800
C	1.555900	-2.037300	2.657000
C	-2.883500	0.353400	2.664700
O	-2.332000	1.863700	0.244600
O	2.537000	1.982400	0.012600
O	1.377200	2.810500	3.024400
O	-1.382000	2.693600	3.177400
C	3.029200	0.575800	2.493100
C	2.899600	0.806300	-2.594900
C	-3.011400	0.976900	-2.405700
O	1.254300	-1.435400	-2.735900
O	-1.524700	-1.343400	-2.762500
C	-5.708700	-2.752300	-0.262500
C	-7.009200	-2.290600	-0.962400
C	-5.022700	-3.836800	-1.126800
C	-6.092200	-3.387100	1.087500
C	5.627700	-2.836300	-0.254200
C	4.943100	-4.013300	0.480300
C	5.935000	-3.285400	-1.695300
C	6.969600	-2.512000	0.444700
C	0.984800	3.895900	2.165500
C	-1.326400	3.136400	4.541100
C	-1.463700	-1.990400	-4.042300
C	1.049800	-2.313800	-1.612200
H	-4.567400	-1.655500	2.047400
H	-4.616900	-1.176200	-2.217100
H	4.431500	-1.392500	-2.339500
H	4.657500	-1.532200	1.943400
H	2.480600	3.429600	-2.863300
H	1.321700	5.568800	-2.898900

H	-1.174500	5.648300	-2.731000
H	-2.451900	3.583600	-2.552600
H	-2.305800	-2.193200	2.570700
H	-1.005900	-4.266500	2.549600
H	1.492300	-4.174200	2.637700
H	2.635400	-2.017600	2.702400
H	-3.221500	1.350000	2.955300
H	-3.305200	-0.345500	3.395600
H	3.544700	0.002600	3.272800
H	3.295500	1.622400	2.647800
H	3.365400	1.779100	-2.760300
H	3.224500	0.171600	-3.424900
H	-3.428300	1.984600	-2.442500
H	-3.439600	0.443100	-3.259900
H	-7.691300	-3.137700	-1.096700
H	-7.525600	-1.530600	-0.366500
H	-6.809600	-1.863600	-1.949700
H	-5.688100	-4.698000	-1.256100
H	-4.766400	-3.461400	-2.121900
H	-4.099800	-4.187500	-0.653300
H	-6.774600	-4.226300	0.918200
H	-5.217500	-3.775800	1.619000
H	-6.601700	-2.673300	1.743200
H	5.581900	-4.903200	0.451400
H	4.747500	-3.779800	1.531000
H	3.987400	-4.265800	0.009200
H	6.594000	-4.159300	-1.675000
H	5.026900	-3.569600	-2.236900
H	6.442200	-2.501200	-2.266800
H	7.628000	-3.387900	0.430700
H	7.486700	-1.690900	-0.063100
H	6.824100	-2.221200	1.489300
H	1.854100	4.553200	2.098900
H	0.727200	3.526000	1.170700
H	0.140200	4.448700	2.580500
H	-0.297700	3.357300	4.843600
H	-1.927200	4.046300	4.595100
H	-1.749000	2.380500	5.213400
H	-2.096200	-2.876900	-3.966500
H	-1.852400	-1.332900	-4.829200
H	-0.440400	-2.287900	-4.288000
H	1.363400	-1.828800	-0.683700
H	0.004500	-2.619200	-1.536000
H	1.681300	-3.184700	-1.796200
C	3.468900	3.069700	0.003800
H	2.885500	3.986800	-0.076600
H	4.063900	3.092800	0.925200
H	4.152300	3.002800	-0.851100
C	-3.101800	3.050200	0.457400
H	-3.842900	3.193000	-0.339100
H	-3.618300	3.026900	1.422900
H	-2.397700	3.882800	0.448400

5) Minimum energy of **1,3-alternate conformer of 5**

Energy = -2390.41353.

0 imaginary frequency

Atomic coordinates of **1,3-alternate conformer of 5.**

C	1.334800	2.601300	-1.327400
C	0.553900	3.690200	-0.805700
C	-0.884000	3.598300	-0.775300
C	-1.536400	2.471400	-1.378100
C	-0.758600	1.603600	-2.120500
C	0.669800	1.635600	-2.065700
C	4.140000	0.386300	-1.493900
C	3.350300	1.123800	-0.603600
C	3.118100	0.571200	0.677200
C	3.524900	-0.747700	0.962800
C	4.301300	-1.440300	0.028600
C	4.665300	-0.878500	-1.199700
C	-1.244500	-1.489500	2.497500
C	-0.516600	-0.795600	3.521900
C	0.926500	-0.806400	3.514700
C	1.633000	-1.517500	2.489300
C	0.895400	-2.315600	1.632200
C	-0.530900	-2.289300	1.620200
C	-4.647700	-1.261000	-0.977800
C	-4.133500	-1.682400	0.252400
C	-3.289700	-0.878700	1.031600
C	-2.978300	0.408600	0.563800
C	-3.420200	0.849600	-0.698500
C	-4.252400	0.004200	-1.435800
C	-1.171900	-0.083200	4.567700
C	-0.473300	0.567200	5.559600
C	0.938000	0.548900	5.555200
C	1.610900	-0.114700	4.555000
C	1.162100	4.867400	-0.283600
C	0.419200	5.894600	0.255900
C	-0.986100	5.790200	0.314600
C	-1.612600	4.670400	-0.186400
C	2.838500	2.494500	-1.047800
O	2.634000	1.279600	1.754600
O	-2.183700	1.239900	1.338800
O	-1.383200	0.627700	-2.872800
O	1.400900	0.723100	-2.785800
C	-3.034200	2.211800	-1.274100
C	-2.762100	-1.418400	2.360200
C	3.141800	-1.420400	2.279900
O	-1.228600	-3.115200	0.766500
O	1.559500	-3.112500	0.718600
C	5.598100	-1.584600	-2.203500
C	6.076800	-2.955800	-1.689800
C	4.870100	-1.805800	-3.549700
C	6.845300	-0.699900	-2.443200
C	-5.592600	-2.127400	-1.832800
C	-4.940800	-2.409500	-3.207300
C	-5.911200	-3.479400	-1.167100
C	-6.925800	-1.371700	-2.047600
C	-1.319500	0.853800	-4.289300
C	1.212400	-0.673600	-2.491200
C	1.532600	-4.512400	1.036300
C	-1.076100	-2.895000	-0.646100

H	4.342800	0.834900	-2.461900
H	4.622200	-2.442900	0.287400
H	-4.372800	-2.669300	0.631500
H	-4.593000	0.353400	-2.406400
H	-2.253900	-0.065200	4.605600
H	-1.009400	1.087700	6.347800
H	1.492700	1.055200	6.339700
H	2.693400	-0.113400	4.568700
H	2.239300	4.976700	-0.335200
H	0.915900	6.785600	0.628200
H	-1.575600	6.596400	0.740700
H	-2.694700	4.627800	-0.156700
H	3.097800	3.218900	-0.272800
H	3.405900	2.787000	-1.939000
H	-3.485900	2.285900	-2.269000
H	-3.503900	2.995100	-0.676600
H	-3.178700	-0.822100	3.173000
H	-3.173600	-2.425000	2.486900
H	3.605300	-0.885100	3.109300
H	3.573100	-2.425900	2.278000
H	6.745300	-3.412100	-2.427200
H	6.631200	-2.867400	-0.749900
H	5.241500	-3.645400	-1.530000
H	5.544600	-2.274900	-4.275100
H	4.004500	-2.464400	-3.422000
H	4.518100	-0.865100	-3.983400
H	7.526000	-1.183200	-3.153400
H	6.575500	0.278400	-2.852100
H	7.391600	-0.533400	-1.508800
H	-5.607800	-3.019500	-3.827000
H	-4.729300	-1.486400	-3.755000
H	-3.997200	-2.952500	-3.088600
H	-6.592200	-4.051700	-1.805300
H	-5.010500	-4.084300	-1.019700
H	-6.398400	-3.350200	-0.195200
H	-7.608700	-1.970100	-2.661200
H	-7.418700	-1.169000	-1.090900
H	-6.773200	-0.414700	-2.555400
H	-1.878900	0.040300	-4.754500
H	-1.783700	1.811900	-4.550600
H	-0.285300	0.841200	-4.646300
H	0.201900	-0.997800	-2.741500
H	1.940100	-1.203500	-3.105300
H	1.420400	-0.873000	-1.437000
H	2.108500	-5.015300	0.257200
H	1.999100	-4.698500	2.011000
H	0.508300	-4.896600	1.043500
H	-1.374900	-1.877300	-0.911200
H	-0.049300	-3.080800	-0.966400
H	-1.750800	-3.602800	-1.130900
C	-2.918300	2.101000	2.212600
H	-2.182900	2.671900	2.781300
H	-3.558100	2.793500	1.653000
H	-3.546200	1.529100	2.906000
C	1.333800	1.881200	1.764400
H	0.626200	1.338500	1.135300

H	0.988900	1.841600	2.798400
H	1.385000	2.922700	1.447200

DFT optimized structures of $Na^+ \subset$ 5 complex

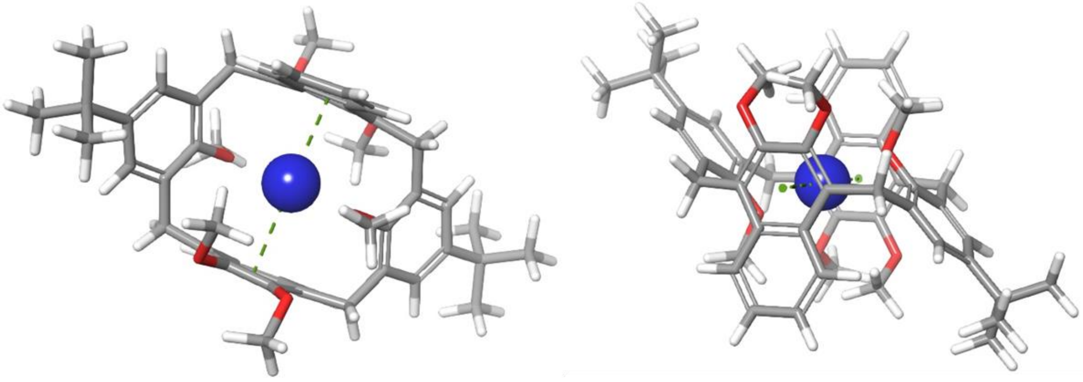

Figure S54. DFT-optimized structures (B3LYP/6-31G/(d,p)) of the $Na^+ \subset$ **5** complex.

1) Atomic coordinates of $Na^+ \subset$ **5 complex.**

C	-1.25151	-2.75459	-0.69411
C	-1.15464	-2.74788	0.6898
C	0.11851	-2.77384	1.34701
C	1.30134	-2.69212	0.62782
C	2.4148	-2.8573	-1.59678
H	3.37972	-2.7753	-1.11449
C	2.3599	-3.05611	-2.95738
H	3.27739	-3.12966	-3.53282
C	1.11065	-3.1934	-3.59901
H	1.06686	-3.38247	-4.66707
C	-0.05343	-3.1	-2.87046
H	-0.99768	-3.22531	-3.38556
C	-0.04418	-2.88423	-1.46337
C	1.23304	-2.79018	-0.80362
C	-2.58885	-1.68864	2.30494
H	-3.539	-1.92115	2.78706
H	-2.70575	-0.76148	1.73145
H	-1.80822	-1.57627	3.06045
C	-0.20239	-4.07867	3.29921
H	-0.13807	-3.94886	4.38022
H	0.50519	-4.85124	2.97948
H	-1.21876	-4.36633	3.01836
C	2.62646	-2.52212	1.35923
H	2.43728	-2.66382	2.42534
H	3.31786	-3.31747	1.06247
C	3.32512	-1.18191	1.12704
C	2.69607	0.04559	1.38132
C	3.32027	1.27411	1.09798
C	4.6294	1.23997	0.61277
H	5.1125	2.18763	0.39579

C	5.32849	0.04184	0.39969
C	4.64414	-1.15239	0.65234
H	5.13976	-2.10207	0.48341
C	6.78414	0.08099	-0.1024
C	7.3828	-1.32778	-0.27352
H	7.39903	-1.88333	0.66971
H	8.41635	-1.24639	-0.62222
H	6.83491	-1.92025	-1.01401
C	7.65286	0.85466	0.9184
H	7.30894	1.88445	1.05178
H	8.69203	0.89429	0.57596
H	7.63785	0.36611	1.89783
C	6.83921	0.79992	-1.47152
H	6.23931	0.26952	-2.21887
H	7.87096	0.8401	-1.83562
H	6.47142	1.82846	-1.41005
C	2.63087	2.61964	1.32498
H	3.28349	3.39522	0.9154
H	2.57175	2.81	2.39829
O	-2.31311	-2.79181	1.42664
O	0.1506	-2.8041	2.72295
O	1.39077	0.05551	1.88831
C	1.25156	2.75456	0.69424
C	1.15462	2.74802	-0.68967
C	-0.11856	2.77397	-1.3468
C	-1.30135	2.69207	-0.62757
C	-2.41471	2.85703	1.5971
H	-3.37965	2.77504	1.11485
C	-2.35974	3.05571	2.95771
H	-3.27721	3.12919	3.5332
C	-1.11047	3.19297	3.5993
H	-1.06664	3.38196	4.66738
C	0.05358	3.09969	2.87069
H	0.99784	3.225	3.38574
C	0.04426	2.88405	1.46358
C	-1.23299	2.79	0.80387
C	2.58884	1.68933	-2.30516
H	1.8081	1.577	-3.06056
H	3.53886	1.92211	-2.78739
H	2.70599	0.76207	-1.73189
C	0.20221	4.07911	-3.29883
H	1.21857	4.36677	-3.01795
H	0.1379	3.94944	-4.37986
H	-0.5054	4.85161	-2.979
C	-2.62649	2.52214	-1.35896
H	-2.43734	2.664	-2.42505
H	-3.3179	3.31744	-1.06208
C	-3.32514	1.18189	-1.12696
C	-2.69599	-0.04558	-1.38111
C	-3.32022	-1.27414	-1.09798
C	-4.62946	-1.24005	-0.61308
H	-5.11259	-2.18773	-0.39626
C	-5.32865	-0.04195	-0.40013
C	-4.64428	1.15231	-0.65259
H	-5.13999	2.10196	-0.48377
C	-6.78442	-0.08116	0.10161

C	-6.8398	-0.80024	1.47064
H	-6.24009	-0.26991	2.2182
H	-7.87164	-0.84049	1.83449
H	-6.47197	-1.82877	1.40915
C	-7.38312	1.32758	0.27275
H	-7.39905	1.88327	-0.67041
H	-8.41679	1.24614	0.6211
H	-6.83548	1.91994	1.0135
C	-7.6529	-0.85473	-0.91947
H	-7.30895	-1.88451	-1.05287
H	-8.69215	-0.89438	-0.57729
H	-7.63764	-0.36608	-1.89885
C	-2.63079	-2.61966	-1.32492
H	-3.28343	-3.39521	-0.91533
H	-2.57163	-2.81007	-2.39822
O	2.31305	2.79225	-1.42657
O	-0.15072	2.80445	-2.72274
O	-1.39053	-0.05545	-1.88772
Na	0.00017	0.00006	0.00024
C	-1.32021	-0.02899	-3.32903
H	-0.27699	-0.19499	-3.59451
H	-1.93695	-0.82668	-3.75204
H	-1.64657	0.93959	-3.7114
C	1.32092	0.02913	3.32964
H	0.27778	0.19512	3.59545
H	1.64742	-0.93942	3.71195
H	1.93779	0.82685	3.75242

Energy = -2552.67746
0 imaginary frequency

DFT optimized structures of $K^+ \subset$ 5 complex

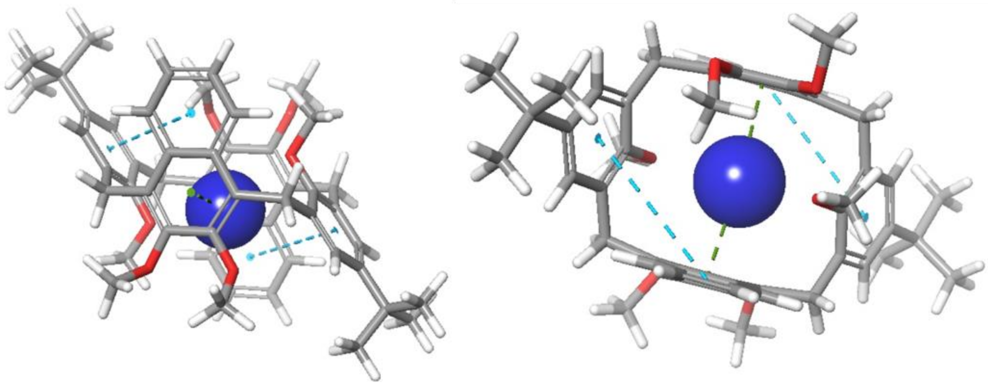

Figure S55. DFT-optimized structures (B3LYP/6-31G/(d,p)) of the $K^+ \subset$ **5** complex.

2) Atomic coordinates of $K^+ \subset$ **5** complex**.**

C	-1.17165800	2.65672000	1.05918100
C	-1.15421500	2.92173300	-0.30295000
C	0.07750100	3.01432800	-1.03768900
C	1.30044800	2.79203800	-0.42063800

C	2.54485500	2.69515800	1.73614100
H	3.47616800	2.76049600	1.19154000
C	2.57650400	2.64766900	3.11143700
H	3.52803200	2.66898400	3.63360800
C	1.36992700	2.59240900	3.84033400
H	1.39067100	2.56863300	4.92550600
C	0.16343200	2.58452900	3.17754700
H	-0.74684300	2.56242200	3.76260400
C	0.08495600	2.64160300	1.75728200
C	1.31841400	2.70054700	1.01428800
C	-2.80110900	4.06820300	-1.64911000
H	-3.88194900	3.96263200	-1.75800200
H	-2.34486800	4.15587700	-2.63684200
H	-2.58417800	4.96215900	-1.05514100
C	0.34820200	4.48110400	-2.89450500
H	0.19396900	4.45278100	-3.97388800
H	1.39754700	4.70701000	-2.68246900
H	-0.28072400	5.26133100	-2.45457600
C	2.56863200	2.62020100	-1.26178000
H	2.30004600	2.78741600	-2.30469300
H	3.29699600	3.39580600	-1.00151500
C	3.26102500	1.25975400	-1.13737900
C	2.65583200	0.07296800	-1.57937800
C	3.22968500	-1.18762800	-1.33064100
C	4.50127000	-1.21842500	-0.75273000
H	4.95013100	-2.19053600	-0.57352700
C	5.20307300	-0.05447300	-0.40360500
C	4.54602000	1.16771200	-0.58348700
H	5.03787800	2.08972300	-0.29458100
C	6.63113300	-0.15854800	0.16544500
C	7.23759700	1.22127700	0.48315300
H	7.30126300	1.85441600	-0.40781200
H	8.25333400	1.09345600	0.86994500
H	6.66084600	1.75632900	1.24481700
C	7.54087300	-0.85754300	-0.87320600
H	7.18777800	-1.86520300	-1.11110700
H	8.56120400	-0.94470500	-0.48431600
H	7.58107800	-0.28583800	-1.80626900
C	6.61433000	-0.98950300	1.47018600
H	5.98631600	-0.51258500	2.23015100
H	7.62821500	-1.07650100	1.87568300
H	6.23573700	-2.00247200	1.30496200
C	2.52064500	-2.49730100	-1.68061300
H	3.19782200	-3.31443300	-1.41593900
H	2.39048300	-2.55291100	-2.76179100
O	-2.35390000	2.88307900	-0.97198900
O	-0.02379800	3.17927200	-2.40326300
O	1.42930700	0.13857100	-2.23907600
C	1.17956700	-2.73295300	-0.98574500
C	1.14989000	-2.84593000	0.39733900
C	-0.08485200	-2.96283100	1.11490100
C	-1.30766800	-2.88881800	0.46656900
C	-2.52464900	-2.98938100	-1.70662400
H	-3.46277300	-3.03432200	-1.17005800
C	-2.53674600	-3.03754000	-3.08236500
H	-3.47947300	-3.11826200	-3.61449800

C	-1.32113700	-2.99680600	-3.79809100
H	-1.32720000	-3.04515800	-4.88257100
C	-0.12444300	-2.90591900	-3.12322900
H	0.79227200	-2.89195800	-3.69868300
C	-0.06303000	-2.85954600	-1.70067300
C	-1.30798800	-2.90847500	-0.97038800
C	2.59030300	-1.82704100	2.03194100
H	1.91367200	-1.89891900	2.88455700
H	3.62179600	-1.96169900	2.35766800
H	2.49013000	-0.84716800	1.55504700
C	0.37021500	-4.36536800	2.96508200
H	1.38092100	-4.60501100	2.62393200
H	0.35273800	-4.30397900	4.05387600
H	-0.32816900	-5.13976600	2.62963700
C	-2.59116100	-2.75030500	1.28331700
H	-2.34233500	-2.96536400	2.32349200
H	-3.31844900	-3.50763800	0.97492400
C	-3.26550900	-1.37700200	1.17272900
C	-2.63929400	-0.19074600	1.59746200
C	-3.21280500	1.07492100	1.36307300
C	-4.48108100	1.11525900	0.77744100
H	-4.92433900	2.09038200	0.60172700
C	-5.18665700	-0.04229700	0.41677900
C	-4.54633000	-1.27163000	0.61150800
H	-5.04544300	-2.18893300	0.32056500
C	-6.59986800	0.07628200	-0.18528700
C	-6.53707800	0.89889400	-1.49429600
H	-5.89162300	0.41118300	-2.23263400
H	-7.53798200	0.99370600	-1.92916900
H	-6.15190800	1.90863500	-1.32442800
C	-7.21595000	-1.29754600	-0.51045400
H	-7.30947900	-1.92475900	0.38205100
H	-8.22018100	-1.15893400	-0.92263900
H	-6.62779100	-1.84419000	-1.25492400
C	-7.52684000	0.79283000	0.82563700
H	-7.16774400	1.79739400	1.06763500
H	-8.53542400	0.89068800	0.40963700
H	-7.59888900	0.22689300	1.76025300
C	-2.51216800	2.38547400	1.74224600
H	-3.19809800	3.19866100	1.49102000
H	-2.38830500	2.41957200	2.82536200
O	2.34209200	-2.87661200	1.07572800
O	-0.04345700	-3.07129400	2.48569600
O	-1.37646200	-0.25058700	2.17685100
K	-0.46549600	-0.00305700	-0.45581700
C	-1.38498800	-0.44674000	3.60239900
H	-0.34793000	-0.38564700	3.92941000
H	-1.97422900	0.33345500	4.09515300
H	-1.79068200	-1.42819100	3.85969900
C	1.53308700	0.29758800	-3.66568800
H	0.51381400	0.31058600	-4.05189100
H	2.03373500	1.23645600	-3.91923200
H	2.08542600	-0.53677500	-4.10950800

Energy = - 2990.28010
0 imaginary frequency

DFT optimized structures of $Cs^+ \subset$ 5 complex

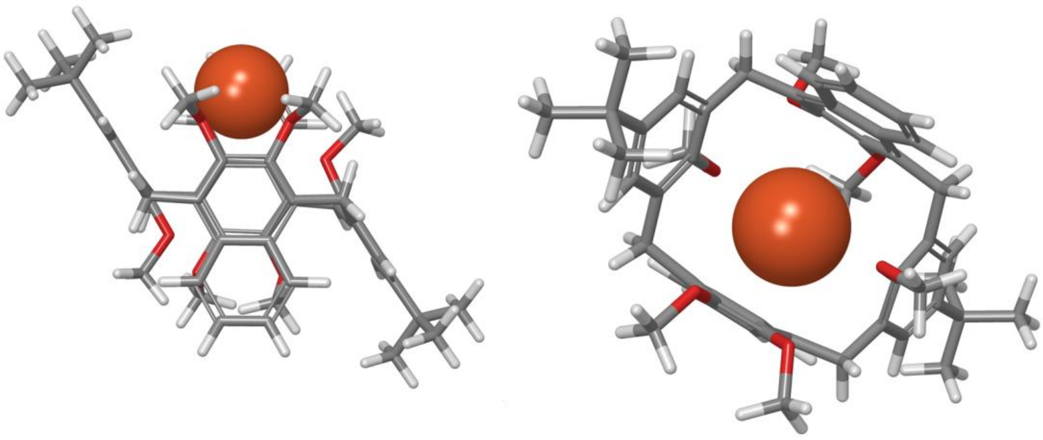

Figure S56. DFT-optimized structures (B3LYP/SDD) of the $Cs^+ \subset$ **5** complex.

3) Atomic coordinates of $Cs^+ \subset$ **5** complex**.**

C	-0.90406700	2.48693900	1.57817900
C	-1.00147100	2.80349500	0.22578900
C	0.15749700	2.92694400	-0.61549500
C	1.44167200	2.74425100	-0.11106100
C	2.87281300	2.66067300	1.94163300
H	3.75189500	2.79637500	1.32602600
C	3.02483900	2.56724800	3.31822300
H	4.01458200	2.62260800	3.76363000
C	1.87862600	2.40978100	4.14764400
H	1.99436700	2.33614700	5.22573900
C	0.61314100	2.36253100	3.57898000
H	-0.24328800	2.25830300	4.23449700
C	0.41534700	2.48122200	2.16518400
C	1.58274500	2.62205000	1.32084700
C	-2.94660700	4.18214400	-0.35511400
H	-3.95553900	4.01334300	-0.73763500
H	-2.42271600	4.90238600	-0.99313300
H	-2.99438000	4.56767700	0.67003600
C	0.13124800	4.46975100	-2.52268600
H	-0.08155600	4.41550900	-3.59253500
H	1.16496800	4.79753600	-2.36255200
H	-0.55239400	5.17656800	-2.03903700
C	2.63830800	2.65926300	-1.06729900
H	2.26895300	2.85055300	-2.07753700
H	3.36565800	3.44855600	-0.83685500
C	3.37022500	1.31112800	-1.04138800
C	2.76512200	0.12149300	-1.50054300
C	3.39126600	-1.13646000	-1.33422400
C	4.70234400	-1.16112500	-0.82551900
H	5.18118200	-2.12954000	-0.70829200
C	5.39710800	0.01529100	-0.46382100
C	4.69471900	1.23326900	-0.55663100
H	5.18054700	2.15676500	-0.25836400
C	6.86151300	-0.07292800	0.02637600

C	7.45817700	1.31605900	0.36571300
H	7.45870200	1.98380200	-0.50573100
H	8.49849100	1.19586700	0.69358800
H	6.90905500	1.80878700	1.17884600
C	7.73815600	-0.71723300	-1.08865800
H	7.38795300	-1.72331200	-1.34914100
H	8.77947200	-0.80018600	-0.74963900
H	7.72223800	-0.10561200	-2.00042600
C	6.93351000	-0.95698600	1.30648600
H	6.34070100	-0.51638500	2.11891400
H	7.97385100	-1.04085900	1.64819900
H	6.55777400	-1.97058500	1.12212800
C	2.68853600	-2.44773800	-1.69709600
H	3.39377700	-3.26333300	-1.49842800
H	2.48924900	-2.46722400	-2.77145700
O	-2.27391200	2.87209800	-0.39053000
O	-0.07209000	3.10495100	-2.00115500
O	1.51379900	0.17988500	-2.18427900
C	1.39464500	-2.71308100	-0.92234200
C	1.45279600	-2.85544700	0.46287900
C	0.27089800	-3.04564400	1.25499500
C	-0.99762400	-2.99201500	0.68845500
C	-2.35339200	-3.12385800	-1.41132000
H	-3.25088800	-3.23697000	-0.81578100
C	-2.45361500	-3.17648500	-2.79672100
H	-3.42026500	-3.32682100	-3.27008700
C	-1.27985900	-3.04973000	-3.59488400
H	-1.35201500	-3.09674500	-4.67823500
C	-0.04050800	-2.88501800	-2.98639700
H	0.83646400	-2.80751000	-3.61845000
C	0.10672800	-2.85233300	-1.55993000
C	-1.08994000	-2.96994800	-0.75007600
C	2.97705500	-1.79057600	2.07255600
H	2.27973100	-1.86306100	2.91114400
H	4.00422400	-1.95264100	2.40422600
H	2.89035600	-0.81238900	1.58746200
C	0.93825000	-4.54859300	3.04243700
H	1.93853400	-4.70248400	2.62410000
H	0.98226400	-4.53393400	4.13361200
H	0.25983300	-5.34226800	2.70450500
C	-2.22946800	-2.93792700	1.59208100
H	-1.90244800	-3.18540300	2.60527900
H	-2.96290900	-3.69889500	1.29870900
C	-2.92490100	-1.56846900	1.57884900
C	-2.28163600	-0.39554300	2.03796600
C	-2.89457800	0.87552300	1.92125500
C	-4.20520000	0.93769500	1.41311500
H	-4.67479400	1.91506300	1.33641500
C	-4.91719900	-0.21541000	1.00945900
C	-4.24478900	-1.45236300	1.08933500
H	-4.74982700	-2.36016200	0.77454000
C	-6.37279300	-0.08375400	0.50143300
C	-6.40949200	0.84507900	-0.74833700
H	-5.80735500	0.42414200	-1.56553100
H	-7.44154900	0.95570900	-1.10706900
H	-6.02384400	1.84643700	-0.52132000

C	-6.98949300	-1.44879900	0.10525800
H	-7.02010100	-2.14402600	0.95404700
H	-8.02071200	-1.29744700	-0.23806000
H	-6.43399100	-1.92715200	-0.71248100
C	-7.25615900	0.53813100	1.62382900
H	-6.89261500	1.52809300	1.92468700
H	-8.29042900	0.65115400	1.27220400
H	-7.26502900	-0.10457500	2.51378200
C	-2.17843300	2.15871300	2.36482200
H	-2.89398700	2.98325200	2.26895500
H	-1.94556100	2.08697700	3.42966700
O	2.70977200	-2.86492300	1.09021800
O	0.40969900	-3.22704000	2.64526300
O	-0.98318700	-0.46615300	2.59232700
Cs	-1.85488600	0.48608000	-2.25416500
C	-0.94690300	-0.77041900	4.03239800
H	0.10192800	-0.70936400	4.32511500
H	-1.54100600	-0.03849800	4.59674400
H	-1.32098300	-1.78151200	4.22429100
C	1.70457300	0.37686000	-3.63522400
H	0.72581100	0.26784500	-4.10728400
H	2.10307000	1.37774600	-3.83926000
H	2.38825500	-0.37916900	-4.04092100

Energy = -2410.10223
0 imaginary frequency

DFT optimized structures of $Li^+ \subset$ 5 complex

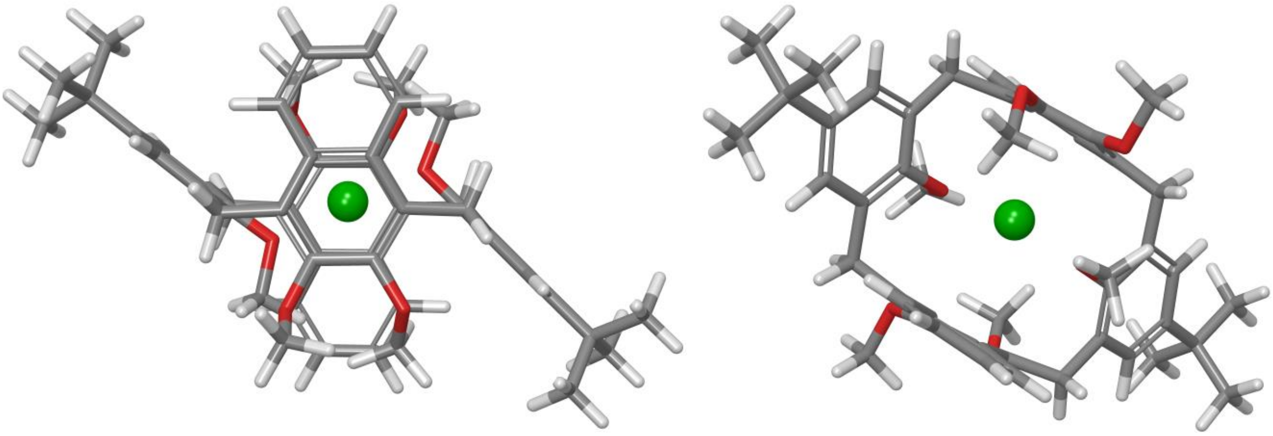

Figure S57. DFT-optimized structures (B3LYP/6-31G/(d,p)) of the $Li^+ \subset$ **5** complex.

C	1.27576000	-2.85097200	0.64188900
C	1.14828100	-2.86294800	-0.73688000
C	-0.13844800	-2.87484200	-1.36042200
C	-1.29828100	-2.76013000	-0.61364600
C	-2.36658800	-2.97158400	1.62640800
H	-3.34186600	-2.87441600	1.16544000
C	-2.28218800	-3.19848000	2.98259800
H	-3.18684900	-3.27706000	3.57769700

C	-1.01873900	-3.35328200	3.59240300
H	-0.95097600	-3.56079900	4.65592200
C	0.13054000	-3.24742800	2.83963600
H	1.08640000	-3.38458100	3.33099900
C	0.08939100	-3.00121600	1.43860600
C	-1.20197200	-2.89350300	0.81109200
C	2.54566200	-1.83531900	-2.41505600
H	3.51591900	-2.05002900	-2.86506800
H	2.60777600	-0.88054300	-1.88215700
H	1.77963500	-1.78398200	-3.19040300
C	0.08507900	-4.18969800	-3.31731800
H	-0.00801000	-4.06538900	-4.39705500
H	-0.63492300	-4.93847000	-2.96992900
H	1.10065900	-4.50925300	-3.06793500
C	-2.62328400	-2.49791500	-1.30642000
H	-2.45923000	-2.59519800	-2.38261700
H	-3.34500600	-3.27771200	-1.04170600
C	-3.29055900	-1.14672300	-1.02558400
C	-2.63598600	0.09203800	-1.13018400
C	-3.31324000	1.31103000	-0.93416000
C	-4.67245100	1.25460100	-0.61402100
H	-5.19188900	2.19464400	-0.45388200
C	-5.37526400	0.04717000	-0.49666700
C	-4.65778900	-1.13389400	-0.71049600
H	-5.16165200	-2.09097800	-0.63630100
C	-6.87366300	0.06195800	-0.13979500
C	-7.47706700	-1.35419300	-0.08546400
H	-7.39638400	-1.86961000	-1.04808200
H	-8.54028200	-1.28858200	0.16537000
H	-6.99667400	-1.97482500	0.67794400
C	-7.64851100	0.87550400	-1.20386900
H	-7.30092700	1.91113200	-1.26055600
H	-8.71593100	0.89622200	-0.95893800
H	-7.53715700	0.42792000	-2.19695700
C	-7.06205400	0.72217500	1.24700700
H	-6.53202700	0.16022700	2.02334300
H	-8.12443400	0.74733500	1.51284800
H	-6.69047000	1.75101800	1.26255700
C	-2.66954400	2.68889600	-1.12850700
H	-3.34589400	3.42363500	-0.68356700
H	-2.65640400	2.90520800	-2.19920200
O	2.29412600	-2.91201600	-1.49622500
O	-0.20604700	-2.90367400	-2.73897000
O	-1.25907700	0.12838600	-1.39955700
C	-1.28087600	2.87488000	-0.54288300
C	-1.13200100	2.81028600	0.83229800
C	0.16295100	2.79954200	1.44028800
C	1.31570900	2.75246000	0.67092800
C	2.34212000	3.08907200	-1.57402400
H	3.32627400	2.97677600	-1.13712600
C	2.23172000	3.38724100	-2.91373700
H	3.12556500	3.50481000	-3.51848400
C	0.95734000	3.56255700	-3.49498600
H	0.87073900	3.82533000	-4.54463000
C	-0.17765600	3.40375500	-2.73150400
H	-1.14366100	3.55322700	-3.19886800

C	-0.10919500	3.08441700	-1.34615300
C	1.19228600	2.95702200	-0.74378900
C	-2.47597700	1.62905100	2.43982400
H	-1.77611100	1.60597000	3.27729700
H	-3.49873800	1.70686700	2.81021700
H	-2.38124600	0.71256600	1.84753400
C	-0.09138800	3.98535200	3.47567600
H	-1.12483900	4.27114000	3.26320000
H	0.02862300	3.79722000	4.54318200
H	0.58793800	4.78610800	3.16483800
C	2.66107800	2.50991300	1.33617100
H	2.51850200	2.60367500	2.41509200
H	3.36397200	3.29939200	1.05277700
C	3.32168500	1.16449300	1.02727300
C	2.67961400	-0.06452600	1.24269400
C	3.31198100	-1.29460500	0.97977000
C	4.62644400	-1.25323400	0.50626800
H	5.11629600	-2.19981300	0.30021100
C	5.32202400	-0.05423200	0.29027400
C	4.64207300	1.13878100	0.55617000
H	5.14048900	2.08979500	0.40585100
C	6.77344200	-0.09079200	-0.22448200
C	6.81104800	-0.78992700	-1.60434300
H	6.20243600	-0.24703900	-2.33522300
H	7.83907300	-0.82773400	-1.98070300
H	6.43788400	-1.81692200	-1.55137300
C	7.37470100	1.31862400	-0.38173600
H	7.40181800	1.85951600	0.56984700
H	8.40400300	1.23810700	-0.74451800
H	6.81739700	1.92320300	-1.10481100
C	7.65256400	-0.88142800	0.77377900
H	7.30623700	-1.91173300	0.89640700
H	8.68758400	-0.91858800	0.41718700
H	7.65044000	-0.40559400	1.75993500
C	2.66071100	-2.65692500	1.23859400
H	3.33396500	-3.41629600	0.83200600
H	2.63260600	-2.82757100	2.31695600
O	-2.26604100	2.78397800	1.60916000
O	0.24869700	2.75111500	2.81316700
O	1.35825100	-0.06205700	1.71130200
Li	0.00024500	0.22254300	0.22659300
C	1.26023200	-0.11486700	3.15513400
H	0.20668700	-0.23611500	3.39807000
H	1.82319100	-0.96866000	3.53646300
H	1.63438300	0.81108400	3.59216700
C	-0.93016600	0.14913600	-2.80869300
H	0.14396200	0.31890200	-2.87529800
H	-1.18024200	-0.80582900	-3.27065200
H	-1.45793300	0.96504500	-3.30586500

Energy = -2397.91076
0 imaginary frequency

NBO and NCI analysis

Natural bond orbital (NBO) analyses were performed with NBO 3.1 version implemented in Gaussian 16 and second-order perturbation theory analysis was performed via single point energy calculations using the b3lyp/6-31G(d,p) level of theory and dichloromethane as solvent. The non-covalent interaction (NCI) analysis was performed with the Multiwfn program [12] and its plot was graphed with VMD program.[13]

Plots (Figures S57 and S58) of the RDG versus the electron density multiplied by the sign of the second Hessian eigenvalue (s = 0.5 a.u.; left) and gradient isosurfaces (s = 0.4 a.u.; right) for the complexes. The coloring scheme was chosen to assist in distinguishing the amplitude of the electron density corresponding to different types of interactions. Marked in green color represent medium-strong (cation···π and Van der Waals) interactions.

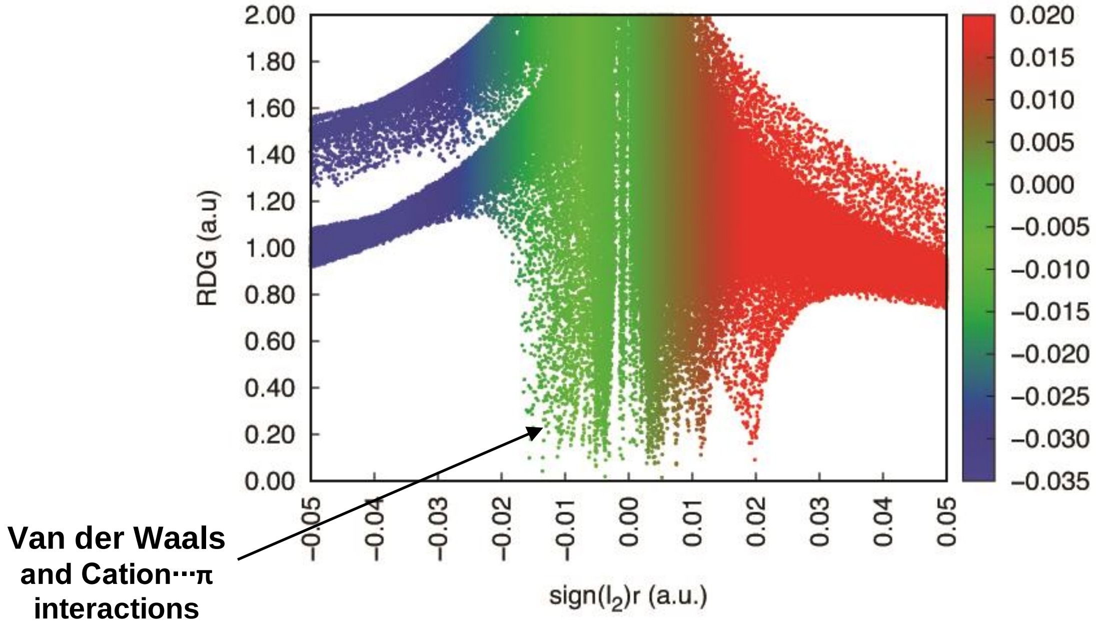

Figure S58. Plot of RDG versus sign(I_2)r for $Na^+ \subset$ **5** complex (NCI-RDG isosurfaces with S = 0.5).

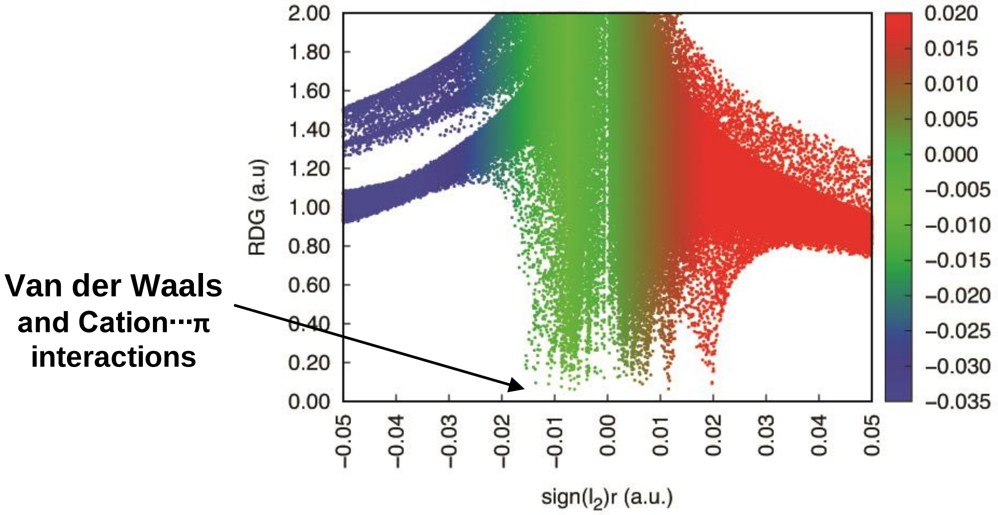

Figure S59. Plot of RDG versus sign(I_2)r for $K^+ \subset$ **5** complex (NCI-RDG isosurfaces with S = 0.5).

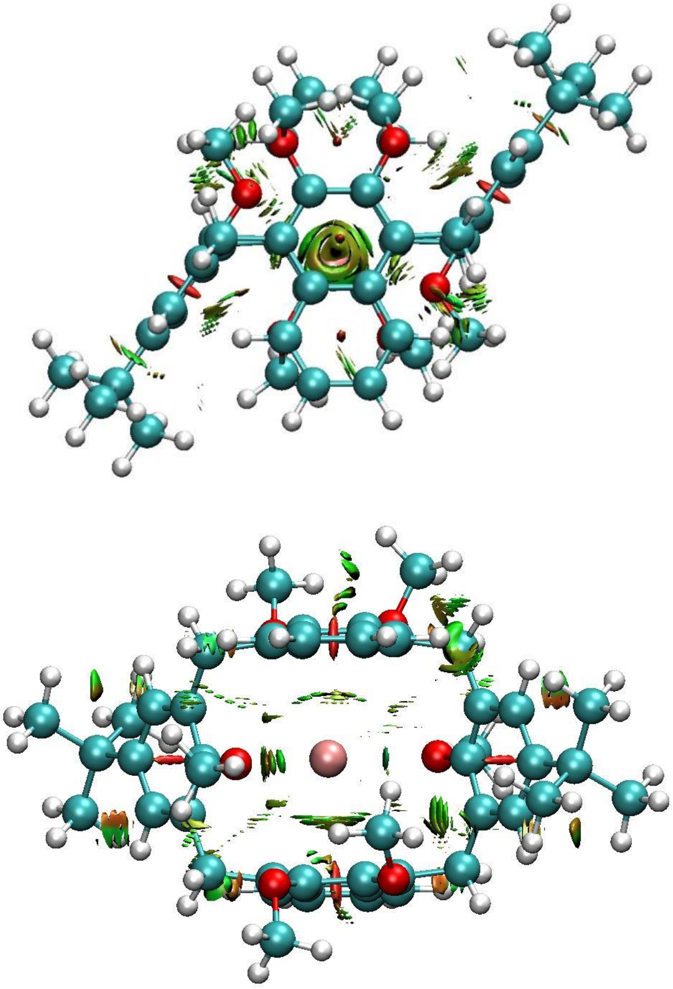

Figure S60. Gradient RDG isosurfaces (0.5) for the noncovalent interaction (NCI) regions in: $K^+ \subset$ **5** complexes.

References:

1) Williams, D. B. G.; Lawton, M. Drying of Organic Solvents: Quantitative Evaluation of the Efficiency of Several Desiccants. *J. Org. Chem*. **2010**, *75*, 8351.
2) Fulmer, G. R.; Miller, A. J. M.; Sherden, N. H.; Gottlieb, H. E.; Nudelman, A.; Stoltz, B.M.; Bercaw, J.E.; Goldberg, K.I. Chemical Shifts of Trace Impurities: Common Laboratory Solvents, Organics, and Gases in Deuterated Solvents Relevant to the Organometallic Chemist. *Organometallics* **2010**, *29*, 2176–2179.
3) Tran, A. H.; O. Miller, D.; E. Georghiou, P. Synthesis and Complexation Properties of “Zorbarene”: A New Naphthalene Ring-Based Molecular Receptor *J. Org. Chem*. **2005,** *70,* 1115-1121.
4) Gaeta, C.; Troisi, F.; Neri, P. endo-Cavity Complexation and Through-the-Annulus Threading of Large Calixarenes Induced by Very Loose Alkylammonium Ion Pairs. *Org. Lett.* **2010**, *12*, 2092-2095.
5) Bakić, M. T.; Iuliano, V.; Talotta, C.; Geremia, S.; Hickey, N.; Spinella, A.; De Rosa, M.; Soriente, A.; Gaeta, C.; Neri, P. Threading of Conformationally Stable Calix[6]arene Wheels Substituted at the Methylene Bridges. *J. Org. Chem.* **2019**, *84*, 11922-11927.
6) Kabsch, W. *Acta Crystallogr.* **2010**, *D66*, 125-132.
7) Kabsch, W. *Acta Crystallogr.* **2010**, *D66*, 133-144.
8) Sheldrick, G. M. *Acta Crystallogr*. **2015**, *A71*, 3-8.
9) Sheldrick, G. M. *Acta Crystallogr*. **2008**, *A64*, 112-122.
10) Farrugia, L. J. *J. Appl. Cryst.* **2012**, *45*, 849−854.
11) Gaussian 16, Revision C.01, Frisch, M. J.; Trucks, G. W.; Schlegel, H. B.; Scuseria, G. E.; Robb, M. A.; Cheeseman, J. R.; Scalmani, G.; Barone, V.; Petersson, G. A.; Nakatsuji, H.; Li, M.; Caricato, M.; Marenich, A. V.; Bloino, J.; Janesko, B. G.; Gomperts, R.; Menucci, B.; Hratchian, H. P.; Ortiz, J. V.; Izmaylov, A. F.; Sonnenberg, J. L.; Williams-Young, D.; Ding, F.;Lipparini, F.; Egidi, F.; Goings, J.; Peng, B.; Petrone, A.; Henderson, T.; Ranasinghe, D.; Zakrzewski, V. G.; Gao, J.; Rega, N.; Zheng, G.; Liang, W.; Hada, M.; Ehara, M.; Toyota, K.; Fukuda, R.; Hasegawa, J.; Ishida, M.; Nakajima, T.; Honda, Y.; Kitao, O.; Nakaj, H.; Vreven, T.; Throssell, K.; Montgomery, J. A.; Peralta, Jr. J. E.; Ogliaro, F.; Bearpark, M. J.; Heyd, J. J.; Brothers, E. N.; Kudin, K. N.; Staroveroy, V. N.; Keith, T. A.; Kobayashi, R.; Normand, J.; Raghavachari, K.; Rendell, A. P.; Burant, J. C.; Iyengar, S. S.; Tomasi, J.; Cossi, M.; Millam, J. M.; Klene, M.; Adamo, C.; Cammi, R.; Ochterski, J. W.; Martin, R. L.; Morokuma, K.; Farkas, O.; Foresman, J. B.; Fox, D. J. Gaussian, Inc., Wallingford CT, 2019.
12) Lu, T.; Chen, F. W. Multiwfn: A Multifunctional Wavefunction Analyzer *J. Comput. Chem.* **2012**, *33*, 580-592.
13) Humphrey, W.; Dalke, A.; Schulten, K. VMD: Visual Molecular Dynamics *J. Mol. Graph.* **1996**, *14*, 33-38.
